# A Carotenoid Health Index Based on Plasma Carotenoids and Health Outcomes

**DOI:** 10.3390/nu3121003

**Published:** 2011-12-08

**Authors:** Michael S. Donaldson

**Keywords:** β-carotene, α-carotene, lycopene, zeaxanthin, β-cryptoxanthin, carotenoid, cancer, diabetes, cardiovascular disease

## Abstract

While there have been many studies on health outcomes that have included measurements of plasma carotenoids, this data has not been reviewed and assembled into a useful form. In this review sixty-two studies of plasma carotenoids and health outcomes, mostly prospective cohort studies or population-based case-control studies, are analyzed together to establish a carotenoid health index. Five cutoff points are established across the percentiles of carotenoid concentrations in populations, from the tenth to ninetieth percentile. The cutoff points (mean ± standard error of the mean) are 1.11 ± 0.08, 1.47 ± 0.08, 1.89 ± 0.08, 2.52 ± 0.13, and 3.07 ± 0.20 µM. For all cause mortality there seems to be a low threshold effect with protection above every cutoff point but the lowest. But for metabolic syndrome and cancer outcomes there tends to be significant positive health outcomes only above the higher cutoff points, perhaps as a triage effect. Based on this data a carotenoid health index is proposed with risk categories as follows: very high risk: <1 µM, high risk: 1-1.5 µM, moderate risk: 1.5-2.5 µM, low risk: 2.5-4 µM, and very low risk: >4 µM. Over 95 percent of the USA population falls into the moderate or high risk category of the carotenoid health index.

## 1. Background

In the scientific research community there has been a strong interest in the relationship between diet and health outcomes. Some initial results from case control studies, ecological studies, and population studies indicated that fruits and vegetables were strongly protective for cancer [1,2,3]. Later prospective observational studies did not generally confirm the protective link between the intakes of fruits and vegetables and chronic diseases [4]. A significant portion of the differences in these two results might be the lack of accuracy in capturing dietary intakes by the use of the food frequency questionnaires (FFQ) in these very large studies. In fact, the FFQs have been called into question for their validity for vitamin C [5], dietary fat [6,7], and dietary fiber [8]. 

A second problem may have been the selection of populations that had very few truly healthy individuals in them, that is, individuals in a state of complete physical, mental, and social well-being and not merely without disease or infirmity (WHO definition). In a few large, prospective cohorts the healthiest 3 percent, based on diet and lifestyle factors and some biochemical measures, were examined for risk of chronic diseases relative to the rest of the study population. In the Nurses’ Health Study there was a 91% reduced risk of diabetes in women [9], and an 82% reduced risk of coronary events [10]. In the men’s Health Professionals Follow-up Study there was a 71% reduced risk of colon cancer in this 3 percent healthy group [11]. In the Cardiovascular Health Study the healthiest 3 percent had an 89% reduction in risk of diabetes among men and women [12]. These decreased incidences of chronic disease are much greater than found in the reports that looked at quartiles or quintiles of fruits and vegetable intakes of the same cohorts [13,14]. Only in the small fraction of healthy people are great reductions of chronic disease found, so that looking at quartiles of intakes of fruits and vegetables is only comparing sick people with sicker people, not sick people versus healthy people. This comparison is an ineffective way to reveal what is required for healthy living. 

A third issue, one that is more fundamental, is that dietary intakes assume that all people absorb, assimilate, and metabolize the eaten food to the same degree and require the same amount of each nutrient to be healthy. The concept of biochemical individuality was brought to light by Roger Williams in 1956 and has been more completely explored with modern techniques since then [15]. However, biochemical and genetic individuality are completely neglected in large cohort studies. 

So, it can be stated that part of the failure of large cohort studies to confirm a positive link between the intake of fruits and vegetables and chronic disease is that largely unhealthy populations were queried with inaccurate FFQs and were assumed to be metabolically all the same.

Perhaps a more fruitful method to search for an association between dietary intakes and chronic diseases is to look at plasma levels of bioactive substances. Such objective measurements are not subject to participant recall on a FFQ, or to pressure to conform to “healthy” expectations while filling out a dietary record. These measurements also account for some of the biochemical individuality of the subjects in a study. Measurement of a bioactive substance takes into account differences in absorption, assimilation, and metabolism of the substance, as well as varying amounts of the substances in the foods themselves. There are fewer metabolic estimates and no subjective recall in using plasma concentrations of bioactive substances.

One of the most measured bioactive substances measured in a large number of population studies is total and individual carotenoids. While carotenoids have been widely shown to correlate with intakes of fruits and vegetables, the correlation coefficients between plasma carotenoids and fruit and vegetable intake were not very high, usually less than 0.5 [16,17,18,19], making carotenoids a less than stellar biomarker of fruit and vegetable intake. As mentioned above, differences in interindividual absorption, assimilation, and metabolism of carotenoids contribute to this problem.

Is there a benefit to measuring plasma carotenoids apart from their use as a biomarker? In a recent study, Del Rio and coworkers found that by changing the total antioxidant capacity of the diet, while holding dietary intake of β-carotene constant, the concentration of plasma β-carotene could be doubled going from a low to high antioxidant diet [20]. Metabolic factors are involved, and the total antioxidant intake can influence the concentration of plasma carotenoids. It appears that there is information about the health of a person found in his or her plasma carotenoid concentration that goes beyond just a biomarker for fruit and vegetable intake. 

At this point there is enough evidence on health outcomes and plasma concentrations of carotenoids that guidelines can be proposed assigning health risk to various concentrations. It is important to note that the population studies of health outcomes do not support the use of dietary supplements to raise plasma carotenoids, but rather indicate that antioxidant-rich and carotenoid-rich foods should be eaten.

## 2. Search Methodology and Analysis

This review gathers together the data from many studies to establish a carotenoid health index based on blood levels. The studies are mainly prospective cohort studies, case-control studies nested within prospective cohorts, other case-control studies, and population-based cross-sectional studies that have measured plasma levels of carotenoids, especially total carotenoids, and health outcomes. This review is limited to human studies that include measurements of serum or plasma levels of carotenoids. 

This review will not go into the details of the health benefits that have been associated with upper concentrations of carotenoids in populations around the world. A spreadsheet summary of that information can be seen in additional file 1. A bibliography of all of the references is available in additional file 2. This html file contains the bibliographic metadata to import the reference data directly into bibliography software.

Articles were searched for in PubMed under “carotenoids” AND “serum OR plasma” while limiting the search results to human studies. From identified studies and reviews, references were checked for any relevant studies that were missed, of which there were several. Searches were also performed for “carotenoids” and “diabetes” or “heart disease” or “cancer” to get specific studies, again limiting the results to human studies.

One hundred and eleven studies were identified that associated plasma or serum carotenoids with health outcomes in people. Of these studies, sixty-two studies had published total carotenoid concentrations and/or concentrations of all six of the most prevalent individual carotenoids present in human blood. Concentration levels were published as means ± standard deviations, as geometric means with 25 and 75 percent levels or 5 and 95 percent levels, or as medians or cutoffs of tertiles, quartiles, or quintiles.

In studies that only reported individual carotenoids, it is not possible to simply add medians of tertiles or quartiles to get the median for the total carotenoids. This method underestimates the lower end of the carotenoid range and overestimates the upper end. Even though the individual carotenoids are highly correlated, individuals that are low in one or two carotenoids are not necessarily low in all of the carotenoids. Likewise, individuals that are high in one carotenoid are not necessarily high in all of the carotenoids. To compensate for this, an estimate of the necessary adjustment was made from 11 studies that reported total carotenoids as well as all of the individual carotenoids. To calculate the adjustment first the medians or means, as given, of the tertiles, quartiles, or quintiles for the individual carotenoids were summed together. Then these sums were compared to the reported medians or means, as given, of the total carotenoids. This percent difference was applied as an adjustment to similar sums of individual carotenoids in the twenty-three studies that only reported individual carotenoids and not total carotenoids. In Table 3 under the “Partitions Reported” column these studies are listed as reporting individual carotenoids with adjusted values. Studies that reported three partitions (means of tertiles or cutoff values for quartiles) were grouped together for the adjustment calculation. Likewise, studies with four values (means of quartiles or cutoff values for quintiles) were grouped together, and studies that reported means and standard deviations were grouped together. Table 1 gives the adjustment calculation for the seven studies that reported three partition values. The other calculations were done in similar manner. Table 2 demonstrates the adjustment applied to one study [21].

**Table 1:** Adjustment Calculation for Reporting Total Carotenoids from Individual Carotenoids. Percent difference between the sum of the individual carotenoids and reported total carotenoid values are given here for studies that gave three partition values.

Reference	Partition Measure	% Diff Cut 1-2	% Diff Cut 2-3	% Diff Cut 3-4
Akbaraly 2007 [22]	quartile cutoffs	23.5%	8.8%	−2.4%
Epplein 2009 [23]	quartile cutoffs	13.5%	5.2%	−4.2%
Epplein 2009 [24]	median tertiles	51.5%	26.6%	0.5%
Goodman 1998 [25]	quartile cutoffs	5.6%	−1.9%	−11.2%
Jenab 2006 [26]	quartile cutoffs	25.3%	12.9%	1.4%
Shardell 2011 [27]	quartile cutoffs	14.8%	3.1%	−2.8%
Yuan 2001 [28]	quartile cutoffs	17.0%	5.40%	1.1%
Average Adjustment		+21.6%	+8.6%	−2.5%

**Table 2:** Total Carotenoid Adjustment Applied to One Study, Dwyer 2004 [21].

	Min	cut 1-2	cut 2-3	cut 3-4	cut 4-5	Max
α-carotene	0.01	0.07	0.11	0.16	0.27	1.06
β-carotene	0.02	0.24	0.42	0.64	1.05	8.00
β-cryptoxanthin	0.01	0.05	0.06	0.08	0.12	0.60
lutein	0.02	0.18	0.23	0.29	0.36	0.81
zeaxanthin	0.01	0.04	0.05	0.06	0.09	0.28
lycopene	0.03	0.31	0.47	0.67	1.03	6.47
TOTAL, SUM	0.10	0.89	1.34	1.90	2.92	17.22
Adjustment		+23%	+14%	+6%	−2%	
Adjusted SUM		1.095	1.53	2.01	2.86	

**Table 3:** Summary Data for the Carotenoid Health Index.

First Author, Year	Partition of Carotenoid Concentrations ^†^	Cutoff 1	Cutoff 2	Cutoff 3	Cutoff 4	Cutoff 5	Benefit, Fraction ^‡^	Which carotenoids? ^§^	Which sex?	What outcome?
Akbaraly 2009 [29]	qnt cutoffs, men		1.61	2.3	2.9		yes, Qn2-5	total	men	all cause mortality
	qnt cutoffs, women		2.3	3.25	4.04		no	total	women	all cause mortality
Bates 2011 [30]	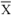 ± SD, men, calc. SD	0.636		1.15		1.664	yes	α-car, lut/zea	men	all cause mortality
	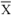 ± SD, women, calc. SD	0.687		1.299		1.911	no	all	women	all cause mortality
deWaart 2001 [31]	90% range, men			0.99			yes	β-cryp, lut/zea	both	all cause mortality
	90% range, women			1.16			yes	β-cryp, lut/zea	both	all cause mortality
Lauretani 2008 [32]	trt cutoffs, 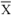 ± SD	1.11	1.466	1.8	1.971	2.49	yes, Qn2-5	total		all cause mortality
	qrt cutoffs, indiv, adj									
Li 2010 [33]	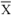 ± SD		0.883	1.297	1.649		yes, Qr2-4	α-car	both	all cause mortality
Mayne 2004 [34]	qrt median, min, max	0.58		1.29		2	yes	lyc, α-car, total		all cause, CVD
Ray 2006 [35]	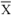 ± SD, men		1.038	1.452	1.995		yes, Qr2-4	total	women	all cause mortality
Sahyoun 1996 [36]	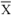 ± SD, women	1.48	1.7	2.39	3.08	3.3	yes, T2-3	total	both	all cause mortality
	qrt cutoffs, indiv carot, total	1.56	1.7	2.53	3.08	3.5				
Shardell 2011 [27]	qrt cutoffs		1.01	1.33	1.75		yes, Qr2-4	total, α-car, lyc	both	all cause mortality
Akbaraly 2007 [22]	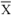 ± SD		1.16	1.65	2.35		yes	lyc, lut/zea	both	cognition
Alipanah 2009 [37]	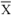 ± SD, women, 5-95%	1.13		2.09		3.03	yes	total	women	walking speed
Yang 2008 [38]	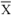 ± SD, men, indiv carot, adj	1.87		4.22		6.57	no		women	osteoporosis
D’Odorico 2000 [39]	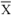 ± SD, women, indiv carot, adj	1.17		1.98		2.79	yes, Qn5	α-car, β-car	both	atheroscl. lesions
	qnt cutoffs, indiv carot, adj	1.46		2.78		4.1				
Dwyer 2004 [21]	qnt medians, indiv carot, adj		1.313	2.014	2.86		yes	lut, zea, β-crypt, α-car	both	intima-media thick.
Hak 2003 [40]	qrt cutoffs, total-lyc	1.117	1.44	1.694	2.051	2.856	no		men	2nd myo. infarction
Hozawa 2009 [41]	qrt medians, cutoffs		1.08	1.29	1.57		yes, Qr4	total w/o lyc	both	hypertension
Hozawa 2007 [42]	qrt cutoffs, ranges, total	0.829	1.056	1.357	1.59	1.99	yes	total w/o lyc	both	inflamm. measures
Beydoun 2011 [43]	trt cutoffs, ranges, indiv, adj		0.863	1.183	1.622		yes, Qr3-4	total	both	metabolic syndrome
Sugiura 2008 [44]	g.mean, 25, 75% indiv, adj men		2.99		4.85		yes, T3	β-car, β-crypt	both	metabolic syndrome
Suzuki 2011 [45]	g.mean, 25, 75% indiv, adj women		2.054	2.728	3.42		yes, T3	β-crypt, β-car	men	metabolic syndrome
	qrt cutoffs, indiv, adj		3.078	4.081	5.16		yes	β-crypt, β-car, α-car	women	metabolic syndrome
Ford 2003 [46]	median, 25, 75%, indiv & total		0.883	1.297	1.649		yes, Qr2-4	all 5 indiv.	both	high CRP
Hughes 2009 [47]	qrt cutoffs, total		0.63	0.92	1.51		no		both	isoprostanes
Akbaraly 2008 [48]	qnt medians, indiv, adjusted		1.82	2.55	3.43		yes, Q4	total	both	dysglycemia
Coyne 2005 [49]	qrt cutoffs, ranges	0.8	1.39	1.93	2.53	4.09	yes	all 5 indiv.	both	fast glucose, OGTT
Hozawa 2006 [50]	trt cutoffs, indiv, adj		0.98	1.29	1.66		yes	total	both	diabetes
Suzuki 2002 [51]	qrt medians, indiv, adj		3.14		5.03		yes	all 5 indiv.	both	high Hb1Ac
Wang 2006 [52]	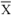 ± SD, total, β-car, cases	0.619	1.32	1.77		2.602	no	all 5 indiv.	women	diabetes
Connett 1989 [53]	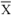 ± SD, total, β-car, controls	0.925		1.59		2.25	yes, Qn4-5	total, β-car	both	lung cancer
	trt medians, total & indiv	1.05		1.81		2.57				
Epplein 2009 [23]	g.mean, 5-95th%, cases	1.67		2.53		3.72	yes, T2-3	total, all 5 indiv.	men	lung cancer
Ito 2003 [54]	g.mean, 5-95th%, controls			1.74			yes, Qr2-4	total, α-car, β-car, lyc, crypt	both	lung cancer
	qrt cutoffs, men			1.87						
Ito 2005 [55]	qrt cutoffs, women		1.22	1.69	2.53		yes, Qr4	α-car, β-car, lyc, crypt	men	lung cancer
	qrt cutoffs, total & indiv.		1.87	2.76	3.93		no		women	lung cancer
Yuan 2001 [28]	qrt cutoffs, cases		0.743	0.941	1.222		yes, Qr3-4	β-crypt, total (smokers)	men	lung cancer
Dorjgochoo [56]	qrt cutoffs, controls		1.89	2.43	2.99		no	none	women	breast cancer
	qrt cutoffs, total & indiv.		1.86	2.21	2.86				women	
Epplein 2009 [23]	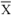 ± SD, cases		2.072	2.771	3.583		no	none	women	breast cancer
Ito 1999 [57]	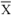 ± SD, controls	0.513		0.847		1.181	yes	all 5 indiv.& total	women	breast cancer
	trt cutoffs, indiv, adj	0.655		1.181		1.707			women	
Kabat 2009 [58]	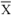 ± SD, controls		1.65		2.4		yes, T3	α-car	women	breast cancer
Maillard 2010 [59]	qrt cutoffs, medians, total	1.39		2.19		2.99	no	none	women	breast cancer
Rock 2005 [60]	trt cutoffs, total	1.038	1.537	2.182	2.867	4.189	yes, Qr4	total	women	breast cancer
Rock 2009 [61]	qnt cutoffs, total & indiv		1.656		2.452		yes, T2-3	total	women	breast cancer
Sato 2002 [62]	qnt medians, total & indiv		1.131	1.683	2.231		yes, Qn5	β-car, lyc	women	breast cancer
Tamimi 2005 [63]	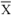 ± SD, cases	1.01	1.48	1.85	2.27	3.05	yes, Qn5	α-car, β-car, lut/zea, total	women	breast cancer
Toniolo 2001 [64]	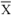 ± SD, controls	1.438		2.306		3.174	yes, Qr2-4	β-car, lut, cryp, total	women	breast cancer
	qrt cutoffs, total, cases	1.527		2.593		3.659			women	
Chang 2005 [65]	qrt cutoffs, total, control		0.82	1.18	1.45		yes	α-car, β-car, β-cryp, lut/zea,	men	prostate cancer
	qrt medians, total & indiv		0.88	1.14	1.63				men	
Gill 2009 [66]	qrt cutoffs, indiv, adj	1.842	2.552	3.31		4.712	no	none	men	prostate cancer
Goodman 2003 [67]	qnt cutoffs, indiv, adj		1.076	1.466	1.856		yes, Qr3-4	lut, zea, β-crypt	both	lung/prostate cancer
Huang 2003 [68]	qnt cutoffs, lyc & total		1.03	1.476	1.91		no	none	men	prostate cancer
Key 2007 [69]	qrt cutoffs, indiv, adj		1.27	1.82	2.32		yes, Qr4	lyc, total	men	prostate cancer, esp. advanced
Lu 2001 [70]	qnt medians, indiv, adj		0.602	0.832	1.222		yes, Q3-4	lyc, zea, lut, β-crypt	men	prostate cancer
Peters 2007 [71]	qrt cutoffs, min, max, indiv, adj	1.233	1.777	2.225	2.708	3.843	no	lyc, β-car	men	prostate cancer
Vogt 2002 [72]	qrt medians, indiv, adj		0.879	1.285	1.745		yes, Q4	lyc	men	prostate cancer, esp. advanced
Zhang 2007 [73]	qrt cutoffs, men	0.673	1.064	1.444		2.218	yes, Q4	lyc	men	prostate cancer
Jiang 2005 [74]	qrt cutoffs, women		1.36	1.92	2.5		yes, Q4	α-car, β-car, total	men	colorectal cancer
	qrt medians, total & indiv		1.91	2.64	3.06		no	all	women	colorectal cancer
Steck-Scott 2004 [75]	trt medians, men, total & indiv	0.972	1.376	1.78		2.495	yes, Q4	α-car, β-car, total	both	polyps in colon
Wakai 2005 [76]	trt medians, women, total & indiv,		1.48	1.59	2.21		yes, T2-3	total	men	colorectal cancer
	qrt cutoffs, total & indiv		2.06	2.55	2.96		no	α-car, total	women	colorectal cancer
Jenab 2006 [26]	qrt cutoffs, medians, indiv, adj		1.294	1.811	2.494		yes, Qr4	β-cryp, zea, total	both	gastric cancer
Persson 2008 [77]	qrt cutoffs, indiv, adj	0.668	1.109	1.723	2.274	2.932	yes, Qr3-4	α-car, β-car	men	gastric cancer
Yuan 2004 [78]	qrt cutoffs, total & indiv.		0.571	0.818	1.138		yes, Qr4	α-car, β-car, lyc	men	gastric cancer
Goodman 1998 [25]	trt medians, indiv, adj		1.71	2.183	2.826		yes, Qr3-4	crypt, total	women	cervical dysplasia
Nagata 1999 [79]	trt cutoffs, indiv, adj	2.357		3.518		5.707	yes, T2-3	α-car, lyc	women	cervical dysplasia
Schiff 2001 [80]	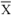 ± SD, cases		2.141		2.728		yes, T3	α-car, β-crypt, lut/zea	women	cervical dysplasia
Nomura 1997 [81]	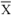 ± SD, controls	0.713		1.521		2.329	yes, T2-3	α-car, β-car, β-crypt, total	men	upper aerodigestive tract cancer
	trt cutoffs, indiv, adj	0.955		1.774		2.593			men	
Delcourt 2006 [82]	trt cutoffs, indiv, adj		1.119		3.085		yes, T2-3	zea, lut, α-car	both	age-related maculopathy, cataract

^†^ qnt = quintile, qrt = quartile, trt = tertile, 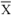
= arithmetic mean, SD = standard deviation, adj = individual carotenoids summed and adjusted to better fit total carotenoid approximations; ^‡^ Qn = quintile, Qr = quartile, T = tertile. The quartiles with statistically different outcomes compared to the lowest partition are listed; ^§^ α-car = α-carotene, β-car = β-carotene, lut = lutein, zea = zeaxanthin, lyc = lycopene.

## 3. Summary of Studies

Table 3 lists the sixty-two studies that reported complete information on carotenoid concentrations and health outcomes. The studies are listed alphabetically within categories of health outcomes. The divisions of the partitions are listed. To aid in establishing cutoff points, the percentiles of the carotenoid concentrations within study populations are grouped together in five levels from percentiles 10-18, 20-40, 50-62.5, 66-80, and 84-90. Any values below the tenth percentile or above the ninetieth percentile were not included in the calculations for establishing cutoff points. These values are descriptive, but were not used in assigning risk of disease. Only eleven studies included this information.

The minimum and maximum concentrations for cutoff point 1 are 0.513 and 2.36 µM, respectively. In the upper level, cutoff point 5, the minimum and maximum are 1.181 and 6.57 µM.

There are nine studies that reported all cause mortality as the principal endpoint, five with cardiovascular endpoints, three with metabolic syndrome endpoints, two with inflammation or lipid peroxidation endpoints, five with diabetic endpoints, five with lung cancer, ten with breast cancer, nine with prostate cancer, three with colorectal endpoints, three with gastric cancer, three with cervical dysplasia, one with upper aerodigestive tract cancer, and one study with age-related maculopathy and cataract endpoints.

Table 4 lists the cutoff points across the percentiles of carotenoid concentrations. For all sixty-two studies the cutoff points (± standard error of the mean) are 1.11 ± 0.08, 1.47 ± 0.08, 1.89 ± 0.08, 2.52 ± 0.13, and 3.07 ± 0.20 µM. There are ten case-control studies included in the analysis, with health outcomes measured across the range of carotenoids within the participants. The averages across the levels did not change significantly, only in the third significant figure, when these case-control studies were excluded. There were 21 measurements of just men and 28 measures of just women. Across all partitions the women have higher concentrations of plasma carotenoids than men, ranging from about 0.15 µM higher at the lower end to more than 0.6 µM higher at cutoff points 3 and 4. There appears to be an international difference in carotenoid concentrations in men and women, most likely due to different gender-based dietary intakes around the world.

**Table 4:** Summary of Data for the Carotenoid Health Index.

Averages across Percentiles, µM	Cutoff 1	Cutoff 2	Cutoff 3	Cutoff 4	Cutoff 5
	10-18%	20-40%	50-62.5%	66-80%	84-90%
All Studies, *N* = 62	1.114	1.468	1.893	2.522	3.069
SEM ^‡^	0.078	0.079	0.085	0.129	0.204
Men Only, *N* = 21	1.091	1.359	1.735	2.263	2.923
Women Only, *N* = 28	1.237	1.800	2.336	3.025	3.411
No Benefit studies, *N* = 10	1.251	1.747	2.357	2.873	3.641
Benefit Studies, *N* = 52	1.123	1.463	1.874	3.012	3.679
Carotenoid Health Index, µM	<1	1 to <1.5	1.5 to <2.5	2.5 to <4	≥4
	Very High Risk	High Risk	Moderate Risk	Low Risk	Very Low Risk
	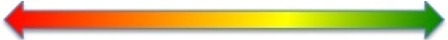				

^‡^ SEM = Standard Error of the Mean. Number of data points for the cutoff points are 32, 56, 72, 52, and 32 for cutoffs 1 to 5, respectively.

Within the sixty-two studies there are 52 that reported a benefit. Some studies had benefits for total carotenoids and three or four individual carotenoids, while others only found a significant effect from one individual carotenoid and even only in a sub-group of the study (men, or just non-smokers, or just advanced prostate cancer), as shown in Table 3. A comparison of the average carotenoid concentration at each cutoff point is given in Table 4. The studies with benefits had a slightly lower bottom cutoff, but the upper cutoff was about the same. Cutoff points 2 and 3 were substantially higher on average in the studies with no benefits, indicating that the populations in the no-benefit studies probably had concentrations that were skewed further to the right, though the highs and lows were not all that different. This might be part of the reason that some benefit was found in some studies but not in others. There may be publication bias towards positive studies in the literature, but an attempt were made within this review to ensure that studies were not excluded or included based on whether or not a benefit was found.

## 4. Threshold, Dose-Response, or Triage Mechanism

One major question regarding carotenoids is whether there is a threshold effect, or whether there is a continuous dose-response effect up through at least the concentrations commonly seen in populations. Within these sixty-two studies there were 40 that had multivariate regression models that gave odds ratios across the range of carotenoids within the study populations (see Table 3, Benefit, Fraction column). From these models it is apparent which fractions of the population are significantly different from the reference fraction, usually the lowest fraction. So, of these 40 studies there were 24 studies in which only the top tertile or top one or two quartiles or quintiles had a statistically different positive health outcome from the lowest reference fraction. There were 16 studies in which only the lowest fraction had a statistically different health outcome from the rest of the population.

Whether there is a threshold or dose-response may depend on the particular outcome. In all of the studies that reported all-cause mortality only the lowest partition of the population seemed to be at risk due to low levels of carotenoids. There seemed to be a threshold for this particular outcome. However, for metabolic syndrome or dysglycemia only the upper tertile or top two quartiles had a significant benefit compared to the lowest tertile or quartile. For cancer outcomes the results are mixed, but overall favor a significant benefit only in the upper concentrations of carotenoids.

Perhaps there is a triage effect for carotenoids, as proposed for other nutrients by Bruce Ames. McCann and Ames demonstrated such an effect for vitamin K [83]. The triage concept is that nutrients will go to the systems of the body that are most critical for short-term survival first. Then if there is a sufficient quantity of the micronutrient all of the other systems of the body will also receive a beneficial amount to prevent subtler dysfunction and age-related disease. The amounts of carotenoids needed to avoid mortality from any cause may be less than the amount to help prevent dysglycemia, diabetes, and some forms of cancer.

The mechanism or mechanisms by which the triage effect might take place with carotenoids is not certain. However, it is known that carotenoids have functions beyond their antioxidant properties. *In vitro* studies with AGS gastric cells showed that β-carotene and lutein both showed anti-inflammatory action. Not only did β-carotene and lutein cause a reduction in the levels of reactive oxygen species when the gastric cells were exposed to H_2_O_2_, indicating antioxidant activity, but there was also an inhibition of the activation of NF-κB and the subsequent expression of IL-8, indicating anti-inflammatory activity [84]. In a study of preterm infants supplemented with β-carotene, lutein, and lycopene compared to unsupplemented preterm infants and full-term infants fed human milk, Rubin and coworkers [85] found that the supplemented infants had decreased concentrations of C-reactive protein, a marker of systemic inflammation. The supplemented group also had greater rod photoreceptor sensitivity, indicating that lutein had protective effects for the retina of pre-term infants as well. More beneficial effects of lutein for retinal health were found in a mouse model of endotoxin-induced uveitis. Along with a reduction in reactive oxygen species in the retina neural cells, the negative effects of inflammation (a decrease in rhodopsin expression, shortening of outer segments of outer photoreceptor cells, levels of STAT3 activation and downstream inflammatory cytokines), were all prevented by lutein treatment given before and concurrently with the endotoxin to induce inflammation in the mice [86]. Together these studies indicate that carotenoids can suppress the inflammatory cascade and normalize cellular function.

Carotenoids have been shown to have specific anti-cancer properties as well. Lycopene induced apoptosis in LNCaP human prostate cancer cells at 0.3 to 3.0 µmol/L [87] or at 1 and 5 µmol/L [88]. Kotake-Nara and coworkers [89] studied the effect of 15 different carotenoids on the growth of three different prostate cancer cell lines. At 20 µmol/L neoxanthin from spinach and fucoxanthin from brown algae decreased cell viability at least 85% in all three cell lines via apoptotic mechanisms. Significant effects were also seen for phytofluene, ζ-carotene, and lycopene. Lycopene, and to a smaller extent β-carotene, enhanced gap junction communication in KB-1 cells derived from human oral cancer [90]. In 10T1/2 cells Zhang and coworkers [91] found that β-carotene, canthaxanthin, lutein, lycopene, and α-carotene all increased gap junctional communication, which was not correlated with the carotenoids’ pro-vitamin A activity or ability to quench lipid peroxidation. Physiological concentrations of β-carotene inhibited cell growth in several human colon adenocarcinoma cell lines by inducing cell cycle arrest in G_2_/M phase and apoptosis [92]. Palozza and coworkers [93] also found that tomatoes digested *in vitro* were able to stop the growth of colon adenocarcinoma cell lines HT-29 and HCT-116 and induce apoptosis. Similar results were also seen in tomatoes genetically modified to express large amounts of β-carotene [94]. Induction of apoptosis by β-carotene via the caveolin-1 pathway, an intracellular signaling pathway usually deregulated in cancer cells, showed yet another mechanism for carotenoids to act [95]. Carotenoids also have antimetastatic activity. Lycopene inhibited adhesion, migration, and invasion of SK-Hep1 human hepatoma cells [96]. *In vivo* inhibition of metastasis was shown in a follow-up study by injecting SK-Hep1 cells into athymic nude mice that had been treated for two weeks with placebo or lycopene or β-carotene. Mice treated with lycopene or β-carotene had significantly reduced numbers of metastasized tumors in the lungs, and smaller cross-sectional area of the tumors [97]. Lycopene, but not β-carotene, also decreased the positive rate of proliferating cellular nuclear antigen (PCNA), the concentration of vascular endothelial growth factor (VEGF), and protein expressions of PCNA, and MMP-9 (matrix metalloproteinase-9) in lung tissue.

Nutragenomic research using microarrays found that lycopene modulated the expression of 391 genes in estrogen-positive breast cancer cells, but not in estrogen-negative breast cancer cells or fibrocystic breast cells [98]. Genetic pathways affected included apoptosis, cell communication, MAPK and cell cycle, xenobiotic metabolism, fatty acid biosynthesis and gap junctional communication.

There are so many pathways that respond to carotenoids that it is quite possible that a triage mechanism is present. Some molecular switches may be inhibited or activated at low concentrations of certain carotenoids while other pathways are abnormally active until the concentrations are much higher. It is unlikely that all cellular pathways are equally affected at the same concentration of carotenoids. So, while there may seem to be a threshold above which some outcomes are not affected, it is much more likely that there are molecular pathways and systems in the body that would benefit from higher plasma levels of carotenoids. 

## 5. Protective Concentration of Total Carotenoids

In Table 4 the proposed Carotenoid Health Index is given. This index is based on the cutoff points of carotenoid concentrations associated with the health outcomes in the sixty-two studies reviewed here. The category most strongly associated with negative health outcomes is less than 1 µM. This category is associated with the lowest quartile or quintile in most population-based studies. In the intermediate range there is some protection, with increasing amounts as the concentration is increased. Category 4, from 2.5 to 4 µM is associated with positive health outcomes in many studies. This amount is greater than the 90th percentile of the USA population in a recent survey [99].

There is a higher category, greater than 4 µM, which may be associated with even greater health benefits. Five of the sixty-two studies had upper partitions of the study population in this range (see Table 3, Cutoff 4 and 5 columns). It isn’t clear exactly where the cutoff should be for this upper range to indicate where the greatest health benefits begin. It could be 5 µM or 3.5 µM rather than 4 µM. There is not a lot of evidence at these higher levels of plasma carotenoids because these high levels are not frequently found in the general population. However, among sub-groups that consume very large amounts of fruits, vegetables, especially leafy greens, and vegetable juice, there may be health benefits associated with high levels of carotenoids. Self-reported health outcomes from people following programs like the Hallelujah Diet, though anecdotal in nature, seem to indicate some benefits from such high intakes of colorful plant foods.

One limitation of The Carotenoid Health Index is that it is limited to people who do not have a chronic thyroid, liver, or kidney disease which causes carotenemia. Though high plasma carotenoid levels are usually due to high dietary intakes and a high overall total antioxidant intake [20], it is possible that a disease process itself might cause abnormally high levels of carotenoids in the plasma as well as in the skin [100]. 

It is an assumption here in this review that when concentrations of total carotenoids are at a protective level, the individual levels will also be at protective levels. In the intermediate range there may be some protection from just one carotenoid or another, but at a sufficiently high range of total carotenoids there should be protective amounts of all of the individual carotenoids. So, if the total carotenoid level is targeted then all of the individual carotenoids will follow. (The only exception to this may be lycopene and astaxanthin that occur in only a few foods.) From a public health standpoint, the main message is to eat a variety of antioxidant-rich and carotenoid-rich fruit and vegetables (not just tomatoes or carrots) to increase the total carotenoids to a concentration that will include protective concentrations of the individual carotenoids.

## 6. Implications of the Carotenoid Health Index

If a low risk category of total plasma carotenoids is greater than 2.5 µM, as the evidence from these 62 studies seems to indicate, and a very low risk category above 4 µM, what are the implications for public health policy? First, having an absolute standard for carotenoid concentrations will help guide future research and interpretation of measurements of carotenoids in populations. Knowing how a study population compares to other populations is critical to understanding why health goals have or haven’t been achieved. The carotenoid health index assists in this interpretation.

A second implication is that the carotenoids health index gives a reference for intervention studies to aim for achieving. If a dietary pattern or a food raises the total carotenoid concentration up to a protective level, then it is more likely to result in positive health outcomes, though this has not yet been proven in randomized controlled trials. If a proposed dietary pattern does not raise the total plasma carotenoid concentration to a protective level, it gives some insights into perhaps why the study did not yield very positive results. The carotenoid health index provides an objective standard to help researchers and scientists design successful trials. The carotenoid health index also gives researchers sufficient scientific justification for applying for research funds for trials that may seem extreme, but will actually raise plasma carotenoid concentrations to protective concentrations, hopefully leading to more positive study results. 

Third, if this review is correct in its assessment of the scientific literature, the carotenoid health index indicates that essentially the entire population of the USA and most of the populations of other countries do not have protective concentrations of plasma carotenoids. More research should be done to demonstrate the most effective ways to improve this measurement. It will most likely include more carotenoid-rich foods, but it may also require replacing junk food and sugary foods and beverages with highly nutritious foods, even foods not high in carotenoids. The total antioxidant capacity of the entire diet needs to be raised to fully utilize the carotenoids that are being eaten [20]. This should be a much higher national priority for all countries that wish to improve the health and productivity of their citizens and to overcome the burden of rising health care costs associated with chronic diseases. 

There are dietary supplements containing mixed carotenoids that will boost a person’s plasma carotenoid concentration. However, the studies summarized here did not use such dietary supplements, so there is very little scientific support for the use of dietary supplements of carotenoids to improve health outcomes. It is true that carotenoids are bioactive compounds, but there are many other compounds in fruits and vegetables that are also beneficial and should be included in the diet. Though unscrupulous marketers may use the carotenoid health index for selling dietary supplements, responsible scientists and food producers need to emphasize the use of foods and whole food products to improve plasma carotenoid concentrations. We have painfully learned that just adding a β-carotene supplement to one’s diet will increase the risk of lung cancer in smokers [101]. Such mistakes should not be repeated.

## 7. Conclusions

The Carotenoid Health Index gives researchers and scientists an objective tool that encompasses the vast literature on plasma carotenoids and health outcomes. From planning clinical trials to evaluating study results, this index gives them a tool for understanding one of their objectives. Rather than just looking at increasing servings of fruits and vegetables, scientists can aim for a biological response that can vary considerably between different individuals even when eating identical diets. However, while more research is being conducted, public health policy has clear evidence that the lack of vegetables in the modern diet is the most important flaw on the dining table, and the carotenoid health index points out this flaw with unwavering clarity. If the intake, absorption, assimilation, and metabolism of fruits and vegetables was such that the tenth percentile of plasma total carotenoids, rather than the ninety-fifth percentile, was near 2.5 µM in the USA and every other nation, then there would be a vast improvement in health and productivity.

## Supplementary Files

Supplementary File 1: PDF-Document (PDF, 1065 KB)

## References

[1] Steinmetz K.A., Potter J.D. (1991). Vegetables, fruit, and cancer. I. Epidemiology.. Cancer Causes Control.

[2] Block G., Patterson B., Subar A. (1992). Fruit, vegetables, and cancer prevention: A review of the epidemiological evidence.. Nutr. Cancer.

[3] Doll R., Peto R. (1981). The causes of cancer: Quantitative estimates of avoidable risks of cancer in the United States today.. J. Natl. Cancer Inst..

[4] Willett W.C. (2001). Diet and cancer: One view at the start of the millennium.. Cancer Epidemiol. Biomark. Prev..

[5] Bingham S., Luben R., Welch A., Low Y.L., Khaw K.T., Wareham N., Day N. (2008). Associations between dietary methods and biomarkers, and between fruits and vegetables and risk of ischaemic heart disease, in the EPIC Norfolk Cohort Study.. Int. J. Epidemiol..

[6] Bingham S.A., Luben R., Welch A., Wareham N., Khaw K.-T., Day N. (2003). Are imprecise methods obscuring a relation between fat and breast cancer?. Lancet.

[7] Freedman L.S., Potischman N., Kipnis V., Midthune D., Schatzkin A., Thompson F.E., Troiano R.P., Prentice R., Patterson R., Carroll R., Subar A.F. (2006). A comparison of two dietary instruments for evaluating the fat-breast cancer relationship.. Int. J. Epidemiol..

[8] Dahm C.C., Keogh R.H., Spencer E.A., Greenwood D.C., Key T.J., Fentiman I.S., Shipley M.J., Brunner E.J., Cade J.E., Burley V.J. (2010). Dietary fiber and colorectal cancer risk: A nested case-control study using food diaries.. J. Natl. Cancer Inst..

[9] Hu F.B., Manson J.E., Stampfer M.J., Colditz G., Liu S., Solomon C.G., Willett W.C. (2001). Diet, lifestyle, and the risk of type 2 diabetes mellitus in women.. N. Engl. J. Med..

[10] Stampfer M.J., Hu F.B., Manson J.E., Rimm E.B., Willett W.C. (2000). Primary prevention of coronary heart disease in women through diet and lifestyle.. N. Engl. J. Med..

[11] Platz E.A., Willett W.C., Colditz G.A., Rimm E.B., Spiegelman D., Giovannucci E. (2000). Proportion of colon cancer risk that might be preventable in a cohort of middle-aged US men.. Cancer Causes Control.

[12] Mozaffarian D., Kamineni A., Carnethon M., Djousse L., Mukamal K.J., Siscovick D. (2009). Lifestyle risk factors and new-onset diabetes mellitus in older adults: The cardiovascular health study.. Arch. Intern. Med..

[13] Bazzano L.A., Li T.Y., Joshipura K.J., Hu F.B. (2008). Intake of fruit, vegetables, and fruit juices and risk of diabetes in women.. Diabetes Care.

[14] Giovannucci E., Rimm E.B., Stampfer M.J., Colditz G.A., Ascherio A., Willett W.C. (1994). Intake of fat, meat, and fiber in relation to risk of colon cancer in men.. Cancer Res..

[15] Williams R. (1998). Biochemical Individuality.

[16] Campbell D.R., Gross M.D., Martini M.C., Grandits G.A., Slavin J.L., Potter J.D. (1994). Plasma carotenoids as biomarkers of vegetable and fruit intake.. Cancer Epidemiol. Biomark. Prev..

[17] Forman M., Lanza E., Yong L., Holden J., Graubard B., Beecher G., Meltiz M., Brown E., Smith J. (1993). The correlation between two dietary assessments of carotenoid intake and plasma carotenoid concentrations: Application of a carotenoid food-composition database.. Am. J. Clin. Nutr..

[18] Jansen M.C.J.F., van Kappel A.L., Ocké M.C., van’t Veer P., Boshuizen H.C., Riboli E., Bueno-de-Mesquita H.B. (2004). Plasma carotenoid levels in Dutch men and women, and the relation with vegetable and fruit consumption. Eur. J. Clin. Nutr..

[19] Al-Delaimy W.K., Ferrari P., Slimani N., Pala V., Johansson I., Nilsson S., Mattisson I., Wirfalt E., Galasso R., Palli D. (2005). Plasma carotenoids as biomarkers of intake of fruits and vegetables: Individual-level correlations in the European Prospective Investigation into Cancer and Nutrition (EPIC).. Eur. J. Clin. Nutr..

[20] Del Rio D., Valtuena S., Pellegrini N., Bianchi M.A., Ardigo D., Franzini L., Scazzina F., Monti L., Zavaroni I., Brighenti F. (2009). Intervention study with a high or low antioxidant capacity diet: Effects on circulating β-carotene.. Eur. J. Clin. Nutr..

[21] Dwyer J.H., Paul-Labrador M.J., Fan J., Shircore A.M., Merz C.N.B., Dwyer K.M. (2004). Progression of carotid intima-media thickness and plasma antioxidants: The los angeles atherosclerosis study.. Arterioscler. Thromb. Vasc. Biol..

[22] Akbaraly N.T., Faure H., Gourlet V., Favier A., Berr C. (2007). Plasma carotenoid levels and cognitive performance in an elderly population: Results of the EVA study.. J. Gerontol. A Biol. Sci. Med. Sci..

[23] Epplein M., Shvetsov Y., Wilkens L., Franke A., Cooney R., Le Marchand L., Henderson B., Kolonel L., Goodman M. (2009). Plasma carotenoids, retinol, and tocopherols and postmenopausal breast cancer risk in the Multiethnic Cohort Study: A nested case-control study.. Breast Cancer Res..

[24] Epplein M., Franke A.A., Cooney R.V., Morris J.S., Wilkens L.R., Goodman M.T., Murphy S.P., Henderson B.E., Kolonel L.N., Le Marchand L. (2009). Association of plasma micronutrient levels and urinary isoprostane with risk of lung cancer: The multiethnic cohort study.. Cancer Epidemiol. Biomark. Prev..

[25] Goodman M.T., Kiviat N., McDuffie K., Hankin J.H., Hernandez B., Wilkens L.R., Franke A., Kuypers J., Kolonel L.N., Nakamura J. (1998). The association of plasma micronutrients with the risk of cervical dysplasia in Hawaii.. Cancer Epidemiol. Biomark. Prev..

[26] Jenab M., Riboli E., Ferrari P., Friesen M., Sabate J., Norat T., Slimani N., Tjonneland A., Olsen A., Overvad K. (2006). Plasma and dietary carotenoid, retinol and tocopherol levels and the risk of gastric adenocarcinomas in the European prospective investigation into cancer and nutrition.. Br. J. Cancer.

[27] Shardell M.D., Alley D.E., Hicks G.E., El-Kamary S.S., Miller R.R., Semba R.D., Ferrucci L. (2011). Low-serum carotenoid concentrations and carotenoid interactions predict mortality in US adults: The third national health and nutrition examination survey.. Nutr. Res..

[28] Yuan J.-M., Ross R.K., Chu X.-D., Gao Y.-T., Yu M.C. (2001). Prediagnostic Levels of serum β-cryptoxanthin and retinol predict smoking-related lung cancer risk in Shanghai, China. Cancer Epidemiol. Biomark. Prev..

[29] Akbaraly T.N., Favier A., Berr C. (2009). Total plasma carotenoids and mortality in the elderly: Results of the Epidemiology of Vascular Ageing (EVA) study.. Br. J. Nutr..

[30] Bates C.J., Hamer M., Mishra G.D. (2011). Redox-modulatory vitamins and minerals that prospectively predict mortality in older British people: The national diet and nutrition survey of people aged 65 years and over.. Br. J. Nutr..

[31] De Waart F., Schouten E., Stalenhoef A., Kok F. (2001). Serum carotenoids, α-tocopherol and mortality risk in a prospective study among Dutch elderly. Int. J. Epidemiol..

[32] Lauretani F., Semba R.D., Dayhoff-Brannigan M., Corsi A.M., Di Iorio A., Buiatti E., Bandinelli S., Guralnik J.M., Ferrucci L. (2008). Low total plasma carotenoids are independent predictors of mortality among older persons: The InCHIANTI study.. Eur. J. Nutr..

[33] Li C., Ford E.S., Zhao G., Balluz L.S., Giles W.H., Liu S. (2011). Serum α-carotene concentrations and risk of death among US Adults: The third national health and nutrition examination survey follow-up study.. Arch. Intern. Med..

[34] Mayne S., Cartmel B., Lin H., Zheng T., Goodwin W.J. (2004). Low plasma lycopene concentration is associated with increased mortality in a cohort of patients with prior oral, pharynx or larynx cancers. J. Am. Coll. Nutr..

[35] Ray A.L., Semba R.D., Walston J., Ferrucci L., Cappola A.R., Ricks M.O., Xue Q.-L., Fried L.P. (2006). Low serum selenium and total carotenoids predict mortality among older women living in the community: The women’s health and aging studies.. J. Nutr..

[36] Sahyoun N.R., Jacques P.F., Russell R.M. (1996). Carotenoids, vitamins C and E, and mortality in an elderly population.. Am. J. Epidemiol..

[37] Alipanah N., Varadhan R., Sun K., Ferrucci L., Fried L.P., Semba R.D. (2009). Low serum carotenoids are associated with a decline in walking speed in older women.. J. Nutr. Health Aging.

[38] Yang Z., Zhang Z., Penniston K.L., Binkley N., Tanumihardjo S.A. (2008). Serum carotenoid concentrations in postmenopausal women from the United States with and without osteoporosis.. Int. J. Vitam. Nutr. Res..

[39] D’Odorico A., Martines D., Kiechl S., Egger G., Oberhollenzer F., Bonvicini P., Sturniolo G.C., Naccarato R., Willeit J. (2000). High plasma levels of α- and β-carotene are associated with a lower risk of atherosclerosis: Results from the Bruneck study.. Atherosclerosis.

[40] Hak A.E., Stampfer M.J., Campos H., Sesso H.D., Gaziano J.M., Willett W., Ma J. (2003). Plasma carotenoids and tocopherols and risk of myocardial infarction in a low-risk population of US male physicians.. Circulation.

[41] Hozawa A., Jacobs D.R., Steffes M.W., Gross M.D., Steffen L.M., Lee D.-H. (2009). Circulating carotenoid concentrations and incident hypertension: The Coronary Artery Risk Development in Young Adults (CARDIA) study.. J. Hypertens..

[42] Hozawa A., Jacobs D.R., Steffes M.W., Gross M.D., Steffen L.M., Lee D.-H. (2007). Relationships of circulating carotenoid concentrations with several markers of inflammation, oxidative stress, and endothelial dysfunction: The Coronary Artery Risk Development in Young Adults (CARDIA)/Young Adult Longitudinal Trends in Antioxidants (YALTA) Study.. Clin. Chem..

[43] Beydoun M.A., Shroff M.R., Chen X., Beydoun H.A., Wang Y., Zonderman A.B. (2011). Serum antioxidant status is associated with metabolic syndrome among US adults in recent national surveys.. J. Nutr..

[44] Sugiura M., Nakamura M., Ogawa K., Ikoma Y., Matsumoto H., Ando F., Shimokata H., Yano M. (2008). Associations of serum carotenoid concentrations with the metabolic syndrome: Interaction with smoking.. Br. J. Nutr..

[45] Suzuki K., Ito Y., Inoue T., Hamajima N. (2011). Inverse association of serum carotenoids with prevalence of metabolic syndrome among Japanese.. Clin. Nutr..

[46] Ford E.S., Liu S., Mannino D.M., Giles W.H., Smith S.J. (2003). C-reactive protein concentration and concentrations of blood vitamins, carotenoids, and selenium among United States adults.. Eur. J. Clin. Nutr..

[47] Hughes K.J., Mayne S.T., Blumberg J.B., Ribaya-Mercado J.D., Johnson E.J., Cartmel B. (2009). Plasma carotenoids and biomarkers of oxidative stress in patients with prior head and neck cancer.. Biomark. Insights.

[48] Akbaraly T.N., Fontbonne A., Favier A., Berr C. (2008). Plasma carotenoids and onset of dysglycemia in an elderly population.. Diabetes Care.

[49] Coyne T., Ibiebele T.I., Baade P.D., Dobson A., McClintock C., Dunn S., Leonard D., Shaw J. (2005). Diabetes mellitus and serum carotenoids: Findings of a population-based study in Queensland, Australia. Am. J. Clin. Nutr..

[50] Hozawa A., Jacobs D.R., Steffes M.W., Gross M.D., Steffen L.M., Lee D.-H. (2006). Associations of serum carotenoid concentrations with the development of diabetes and with insulin concentration: Interaction with smoking: The Coronary Artery Risk Development in Young Adults (CARDIA) study.. Am. J. Epidemiol..

[51] Suzuki K., Ito Y., Nakamura S., Ochiai J., Aoki K. (2002). Relationship between serum carotenoids and hyperglycemia: A population-based cross-sectional study.. J. Epidemiol..

[52] Wang L., Liu S., Pradhan A.D., Manson J.E., Buring J.E., Gaziano J.M., Sesso H.D. (2006). Plasma lycopene, other carotenoids, and the risk of type 2 diabetes in women.. Am. J. Epidemiol..

[53] Connett J.E., Kuller L.H., Kjelsberg M.O., Polk B.F., Collins G., Rider A., Hulley S.B.  (1989). Relationship between carotenoids and cancer. The Multiple Risk Factor Intervention Trial (MRFIT) study.. Cancer.

[54] Ito Y., Wakai K., Suzuki K., Tamakoshi A., Seki N., Ando M., Nishino Y., Kondo T., Watanabe Y., Ozasa K., Ohno Y. (2003). JACC study group serum carotenoids and mortality from lung cancer: A case-control study nested in the Japan Collaborative Cohort (JACC) study.. Cancer Sci..

[55] Ito Y., Wakai K., Suzuki K., Ozasa K., Watanabe Y., Seki N., Ando M., Nishino Y., Kondo T., Ohno Y., Tamakoshi A. (2005). Lung cancer mortality and serum levels of carotenoids, retinol, tocopherols, and folic acid in men and women: A case-control study nested in the JACC Study.. J. Epidemiol..

[56] Dorjgochoo T., Gao Y.-T., Chow W.-H., Shu X.-O., Li H., Yang G., Cai Q., Rothman N., Cai H., Franke A.A., Zheng W., Dai Q. (2009). Plasma carotenoids, tocopherols, retinol and breast cancer risk: Results from the Shanghai Women Health Study (SWHS).. Breast Cancer Res. Treat..

[57] Ito Y., Gajalakshmi K.C., Sasaki R., Suzuki K., Shanta V. (1999). A study on serum carotenoid levels in breast cancer patients of Indian women in Chennai (Madras), India. J. Epidemiol..

[58] Kabat G.C., Kim M., Adams-Campbell L.L., Caan B.J., Chlebowski R.T., Neuhouser M.L., Shikany J.M., Rohan T.E. (2009). Longitudinal study of serum carotenoid, retinol, and tocopherol concentrations in relation to breast cancer risk among postmenopausal women.. Am. J. Clin. Nutr..

[59] Maillard V., Kuriki K., Lefebvre B., Boutron‐Ruault M., Lenoir G.M., Joulin V., Clavel‐Chapelon F., Chajès V. (2010). Serum carotenoid, tocopherol and retinol concentrations and breast cancer risk in the E3N‐EPIC study. Int. J. Cancer.

[60] Rock C.L., Flatt S.W., Natarajan L., Thomson C.A., Bardwell W.A., Newman V.A., Hollenbach K.A., Jones L., Caan B.J., Pierce J.P. (2005). Plasma carotenoids and recurrence-free survival in women with a history of breast cancer.. J. Clin. Oncol..

[61] Rock C.L., Natarajan L., Pu M., Thomson C.A., Flatt S.W., Caan B.J., Gold E.B., Al-Delaimy W.K., Newman V.A., Hajek R.A., Stefanick M.L., Pierce J.P., Women’s Healthy Eating and Living Study Group (2009). Longitudinal biological exposure to carotenoids is associated with breast cancer-free survival in the women’s healthy eating and living study.. Cancer Epidemiol. Biomark. Prev..

[62] Sato R., Helzlsouer K.J., Alberg A.J., Hoffman S.C., Norkus E.P., Comstock G.W. (2002). Prospective study of carotenoids, tocopherols, and retinoid concentrations and the risk of breast cance. Cancer Epidemiol. Biomark. Prev..

[63] Tamimi R.M., Hankinson S.E., Campos H., Spiegelman D., Zhang S., Colditz G.A., Willett W.C., Hunter D.J. (2005). Plasma carotenoids, retinol, and tocopherols and risk of breast cance. Am. J. Epidemiol..

[64] Toniolo P., van Kappel A.L., Akhmedkhanov A., Ferrari P., Kato I., Shore R.E., Riboli E. (2001). Serum carotenoids and breast cancer.. Am. J. Epidemiol..

[65] Chang S., Erdman J.W., Clinton S.K., Vadiveloo M., Strom S.S., Yamamura Y., Duphorne C.M., Spitz M.R., Amos C.I., Contois J.H., Gu X., Babaian R.J., Scardino P.T., Hursting S.D. (2005). Relationship between plasma carotenoids and prostate cancer. Nutr. Cancer.

[66] Gill J.K., Franke A.A., Morris J.S., Cooney R.V., Wilkens L.R., Le Marchand L., Goodman M.T., Henderson B.E., Kolonel L.N. (2009). Association of selenium, tocopherols, carotenoids, retinol, and 15-isoprostane F2t in serum or urine with prostate cancer risk: The multiethnic cohort.. Cancer Causes Control.

[67] Goodman G.E., Schaffer S., Omenn G.S., Chen C., King I. (2003). The association between lung and prostate cancer risk, and serum micronutrients. Cancer Epidemiol. Biomark. Prev..

[68] Huang H.-Y., Alberg A.J., Norkus E.P., Hoffman S.C., Comstock G.W., Helzlsouer K.J. (2003). Prospective study of antioxidant micronutrients in the blood and the risk of developing prostate cancer.. Am. J. Epidemiol..

[69] Key T.J., Appleby P.N., Allen N.E., Travis R.C., Roddam A.W., Jenab M., Egevad L., Tjonneland A., Johnsen N.F., Overvad K. (2007). Plasma carotenoids, retinol, and tocopherols and the risk of prostate cancer in the European prospective investigation into cancer and nutrition study.. Am. J. Clin. Nutr..

[70] Lu Q.-Y., Hung J.-C., Heber D., Go V.L.W., Reuter V.E., Cordon-Cardo C., Scher H.I., Marshall J.R., Zhang Z.-F. (2001). Inverse associations between plasma lycopene and other carotenoids and prostate cancer.. Cancer Epidemiol. Biomark. Prev..

[71] Peters U., Leitzmann M.F., Chatterjee N., Wang Y., Albanes D., Gelmann E.P., Friesen M.D., Riboli E., Hayes R.B. (2007). Serum lycopene, other carotenoids, and prostate cancer risk: A nested case-control study in the prostate, lung, colorectal, and ovarian cancer screening trial.. Cancer Epidemiol. Biomark. Prev..

[72] Vogt T.M., Mayne S.T., Graubard B.I., Swanson C.A., Sowell A.L., Schoenberg J.B., Swanson G.M., Greenberg R.S., Hoover R.N., Hayes R.B., Ziegler R.G. (2002). Serum lycopene, other serum carotenoids, and risk of prostate cancer in US blacks and white. Am. J. Epidemiol..

[73] Zhang J., Dhakal I., Stone A., Ning B., Greene G., Lang N.P., Kadlubar F.F. (2007). Plasma carotenoids and prostate cancer: A population-based case-control study in arkansas.. Nutr. Cancer.

[74] Jiang J., Suzuki S., Xiang J., Kuriki K., Hosono A., Arakawa K., Wang J., Nagaya T., Kojima M., Katsuda N., Tokudome S. (2005). Plasma carotenoid, α-tocopherol and retinol concentrations and risk of colorectal adenomas: A case-control study in Japan. Cancer Lett..

[75] Steck-Scott S., Forman M.R., Sowell A., Borkowf C.B., Albert P.S., Slattery M., Brewer B., Caan B., Paskett E., Iber F., Kikendall W., Marshall J., Shike M., Weissfeld J., Snyder K., Schatzkin A., Lanza E. (2004). he polyp prevention trial study group carotenoids, vitamin a and risk of adenomatous polyp recurrence in the polyp prevention trial.. Int. J. Cancer.

[76] Wakai K., Suzuki K., Ito Y., Kojima M., Tamakoshi K., Watanabe Y., Toyoshima H., Hayakawa N., Hashimoto S., Tokudome S., Suzuki S., Kawado M., Ozasa K., Tamakoshi A. (2005). Serum carotenoids, retinol, and tocopherols, and colorectal cancer risk in a Japanese cohort: Effect modification by sex for carotenoids.. Nutr. Cancer.

[77] Persson C., Sasazuki S., Inoue M., Kurahashi N., Iwasaki M., Miura T., Ye W., Tsugane S. (2008). lasma levels of carotenoids, retinol and tocopherol and the risk of gastric cancer in Japan: A nested case-control study.. Carcinogenesis.

[78] Yuan J.-M., Ross R.K., Gao Y.-T., Qu Y.-H., Chu X.-D., Yu M.C. (2004). Prediagnostic levels of serum micronutrients in relation to risk of gastric cancer in Shanghai, China. Cancer Epidemiol. Biomark. Prev..

[79] Nagata C., Shimizu H., Yoshikawa H., Noda K., Nozawa S., Yajima A., Sekiya S., Sugimori H., Hirai Y., Kanazawa K., Sugase M., Kawana T. (1999). Serum carotenoids and vitamins and risk of cervical dysplasia from a case-control study in Japan.. Br. J. Cancer.

[80] Schiff M.A., Patterson R.E., Baumgartner R.N., Masuk M., van Asselt-King L., Wheeler C.M., Becker T.M. (2001). Serum carotenoids and risk of cervical intraepithelial neoplasia in southwestern American Indian women.. Cancer Epidemiol. Biomark. Prev..

[81] Nomura A.M., Ziegler R.G., Stemmermann G.N., Chyou P.H., Craft N.E. (1997). Serum micronutrients and upper aerodigestive tract cancer.. Cancer Epidemiol. Biomark. Prev..

[82] Delcourt C., Carriere I., Delage M., Barberger-Gateau P., Schalch W., POLA Study Group (2006). Plasma lutein and zeaxanthin and other carotenoids as modifiable risk factors for age-related maculopathy and cataract: The POLA study.. Invest. Ophthalmol. Vis. Sci..

[83] McCann J.C., Ames B.N. (2009). Vitamin K, an example of triage theory: Is micronutrient inadequacy linked to diseases of aging?. Am. J. Clin. Nutr..

[84] Kim Y., Seo J.H., Kim H. (2011). β-carotene and lutein inhibit hydrogen peroxide-induced activation of NF-κB and IL-8 expression in gastric epithelial AGS cells.. J. Nutr. Sci. Vitaminol..

[85] Rubin L.P., Chan G.M., Barrett-Reis B.M., Fulton A.B., Hansen R.M., Ashmeade T.L., Oliver J.S., Mackey A.D., Dimmit R.A., Hartmann E.E., Adamkin D.H.  (2011). Effect of carotenoid supplementation on plasma carotenoids, inflammation and visual development in preterm infants.. J. Perinatol..

[86] Sasaki M., Ozawa Y., Kurihara T., Noda K., Imamura Y., Kobayashi S., Ishida S., Tsubota K. (2009). Neuroprotective effect of an antioxidant, lutein, during retinal inflammation.. Investig. Ophthalmol. Vis. Sci..

[87] Hantz H.L., Young L.F., Martin K.R. (2005). Physiologically attainable concentrations of lycopene induce mitochondrial apoptosis in LNCaP human prostate cancer cells.. Exp. Biol. Med..

[88] Hwang E.-S., Bowen P.E. (2004). Cell cycle arrest and induction of apoptosis by lycopene in LNCaP human prostate cancer cells.. J. Med. Food.

[89] Kotake-Nara E., Kushiro M., Zhang H., Sugawara T., Miyashita K., Nagao A. (2001). Carotenoids affect proliferation of human prostate cancer cells.. J. Nutr..

[90] Livny O., Kaplan I., Reifen R., Polak-Charcon S., Madar Z., Schwartz B. (2002). Lycopene inhibits proliferation and enhances gap-junction communication of KB-1 human oral tumor cells.. J. Nutr..

[91] Zhang L.-X., Cooney R.V., Bertram J.S. (1991). Carotenoids enhance gap junctional communication and inhibit lipid peroxidation in C3H/10T1/2 cells: Relationship to their cancer chemopreventive action.. Carcinogenesis.

[92] Palozza P., Serini S., Maggiano N., Angelini M., Boninsegna A., Di Nicuolo F., Ranelletti F.O., Calviello G. (2002). Induction of cell cycle arrest and apoptosis in human colon adenocarcinoma cell lines by β-carotene through down-regulation of cyclin A and Bcl-2 family proteins.. Carcinogenesis.

[93] Palozza P., Serini S., Boninsegna A., Bellovino D., Lucarini M., Monastra G., Gaetani S. (2007). The growth-inhibitory effects of tomatoes digested *in vitro* in colon adenocarcinoma cells occur through down regulation of cyclin D1, Bcl-2 and Bcl-xL.. Br. J. Nutr..

[94] Palozza P., Bellovino D., Simone R., Boninsegna A., Cellini F., Monastra G., Gaetani S. (2009). Effect of β-carotene-rich tomato lycopene β-cyclase (*tlcy-b*) on cell growth inhibition in HT-29 colon adenocarcinoma cells.. Br. J. Nutr..

[95] Palozza P., Sestito R., Picci N., Lanza P., Monego G., Ranelletti F.O. (2008). The sensitivity to β-carotene growth-inhibitory and proapoptotic effects is regulated by caveolin-1 expression in human colon and prostate cancer cells.. Carcinogenesis.

[96] Hwang E.-S., Lee H.J. (2006). Inhibitory effects of lycopene on the adhesion, invasion, and migration of SK-Hep1 human hepatoma cells.. Exp. Biol. Med..

[97] Huang C.-S., Liao J.-W., Hu M.-L. (2008). Lycopene inhibits experimental metastasis of human hepatoma SK-Hep-1 cells in athymic nude mice.. J. Nutr..

[98] Chalabi N., Satih S., Delort L., Bignon Y.-J., Bernard-Gallon D.J. (2007). Expression profiling by whole-genome microarray hybridization reveals differential gene expression in breast cancer cell lines after lycopene exposure.. Biochim. Biophys. Acta.

[99] Centers for Disease Control and Prevention (CDC) (2008). National Report on Biochemical Indicators of Diet and Nutrition-Vitamins A and E and Carotenoids.

[100] Maharshak N., Shapiro J., Trau H. (2003). Carotenoderma-A review of the current literature.. Int. J. Dermatol..

[101] The Alpha-Tocopherol Beta Carotene Cancer Prevention Study Group (1994). The effect of vitamin E and beta carotene on the incidence of lung cancer and other cancers in male smokers.. N. Engl. J. Med..

