# Supplementary material for: A Carotenoid Health Index Based on Plasma Carotenoids and Health Outcomes
Source: Nutrients. 2011 Dec 8;3(12):1003–22. doi: 10.3390/nu3121003 (PMC3260489; doi:10.3390/nu3121003)
Supplement: Supplementary File 1: — PDF-Document (PDF, 1065 KB) [file nutrients-03-01003-s001.pdf]

Supplementary Materials

Summary Overview of Plasma Carotenoids and Health Outcomes

Description: Spreadsheet summary of data from 111 studies of plasma carotenoids and health outcomes. Information includes references, study name and country, study type, % female, age range, cohort size, follow-up time, number of cases and controls (if applicable), statistical adjustments, range of carotenoids, effect size, and additional comments.

Study, N=111	Study, Country	Study Type	% female	Age range	Cohort Size	Follow-up, years	Cases (and controls, if applicable)	Adjustments	Range of Carotenoids	Effect Size, (95% CI) from lowest to highest	Comments
**All Cause Mortality, N=12**											
Akbaraly 2009	Epidemiology of Vascular Aging, France	Prosp. Cohort	58.6	59-70	1389	9	61 men, 32 women	Age, education level, marital status, smoking, alcohol, diabetes, hypertension, cardiovascular antecedents, dyslipidaemia, BMI	Quintile cut-offs, total carotenoids, men <1.36, 1.86, 2.3, ≥2.9 µM; women <2.0, 2.60, 3.25, ≥4.04 µM	Men: HR 2.94 (1.21, 7.17)<br>Women: No signif effect	Sex difference. Women had higher carotenoid levels. In men no signif dif between Q2, Q3, Q4, and Q5, suggesting threshold effect.
Bates 2011	British National Diet and Nutrition Survey, UK	Prosp. Cohort	49	65+, X,SD (76.6± 7.4)	1054	14	398 men, 320 women	Age, sex	Mean±SD, total (estimated SD) Men: 1.150±0.514; Women 1.299±0.612 µM. Individual carotenoid values for mean, median, range given in text but not for total carotenoids.	In men, HR=0.90 (0.81, 0.99) p=0.04 per SD for alpha-carotene, lutein/zeaxanthin HR=0.91 (0.83-1.00) p=0.04; for cancer mortality a-carotene HR=0.67 (0.47-0.96) p=0.03	HRs are only age and sex adjusted. Not significant in multivariate models. Plasma vitamin C also a factor, but not in multivariate analysis. Plasma Se, Zn, Cu, Fe were stronger factors.
Buijise 2005	SENECA, Survey in Europe on Nutrition and the Elderly, a Concerted Action, Europe	Prosp. Cohort	50	70-75	1168	10	388	age, sex, BMI, cholesterol, HDL cholesterol, smoking, alcohol, physical activity, SENECA center, alpha-tocopherol	Tertiles of carotenes, X±SD, µM: 0.22±0.12; 0.54±0.09; 1.12±0.39	for increment of 0.39 µM carotenes, RR 0.79 (0.70, 0.89) total mortality	cancer: RR 0.59 (0.44, 0.79) CVD: RR 0.83 (0.70. 1.00), lower risk of CVD only for those w/ BMI<25; for those w/ BMI <25 RR=0.67 (0.49, 0.94). More men with low carotenes: 70%, 47%, and 34% men in low, mid, and high tertile of carotenes.
DeWaart	Elderly Dutch, Netherlands	Prosp. Cohort	47.5	65-85	638	7.2	171 (108 men, 63 women)	age, sex, smoking, cholesterol, BMI, alcohol, antiox suppl. Use, phylical activity score, CVD, lung disease, hypertension, cancer	90% range. Men: 0.42 - 1.86; Women: 0.54 - 2.46)	For oxygenated carotenoids HR=1.73 (1.12, 2.67) tertiles. Beta-cryptoxanthin HR=1.52 (1.00, 2.32)	No significant trend for total carotenoids, but signif. trend seen for sum of beta-cryptoxanthin, lutein, and zeaxanthin and for beta-cryptoxanthin by itself.
Hu 2004	MacArthur Studies of Successful Aging, USA	Prosp. Cohort	54	74±2.7	672	7	98 men (31.9%), 54 women (14.8%)	age, race, CRP, IL-6,tota Chol, HDL-chol, BMI, waist-hip ratio, coronary artery disease, hypertension, diabetes, stroke, cancer, smoking, alcohol, vit-A or beta-carotene suppl.	Men: mean beta-carotene 0.20±0.20 µM; Women: 0.30±0.37 µM	Men: beta-carotene RR=2.30 (1.23, 4.31)<br>Women: RR=0.85 (0.42, 1.75)	Both low beta-carotene and high inflammation (CRP, IL-6) RR=3.78 (1.69, 8.47) for men only. Mortality about same in bottom 2 quartiles, and for the top 2 quartiles, so these were combined.
Ito 2002	rural Hokkaido, Japan	Prosp. Cohort	61.1	elderly	2,444 (994 men, 1,495 women)	9	146 (94 men, 52 women)	Age, sex for reported HRs. When also adjusted for smoking, alcohol, total chol, glutmic pyruvic transaminase activity results similar.	Geometric means only, for controls.<br>A-carotene: M 0.088, W 0.138 µM<br>B-carotene: men 0.457, W 1.039 µM<br>b-crypto: M 0.214, W 0.403<br>lut/zea: M 1.023, W 1.266<br>lycopene: M 0.213, W 0.311<br>Total carotenoids: M 2.210, W 3.435 µM	all-cause mortality HRs:<br>lycopene: 0.44 (0.28, 0.69); beta-carotene: 0.59 (0.39, 0.90)<br>zeaxanthin/lutein: 0.61 (0.40. 0.93); total carotenoids: 0.50 (0.33, 0.76)	Cancer mortality:<br>Lycopene: 0.36 (0.19, 0.69)<br>beta-carotene: 0.53 (0.29, 0.95)<br>zeaxanthin/lutein: 0.73 (0.43, 1.25)<br>total carotenoids: 0.52 (0.30, 0.92)
Lauretani 2008	InCHIANTI, Italy (Aging in the Chianti area)	Prosp. Cohort	56.7	65+	1,043	8	310 (29.7%)	Age, education, smoking, BMI, energy intake, chronic diseases	tertile cut-off: 1.466, 1.971 µM mean level 1.80±0.69 µM (±2 SD = 0.42 - 3.18 µM)	HR, 0.81 (0.65, 0.99)	When IL-6 included as a covariate, HR=0.73 (0.52, 0.98). Age, sex, lower education, higher BMI, lower total energy, smoking, congestive heart failure, stroke all significant risk factors for mortality. Not a threshold effect here.
Li 2010	NHANES III follow-up study, USA	Prosp. Cohort	52.12	20+	15,318	13.9	3,810	Age, sex, race/ethnicity, alcohol, physical activity, BMI, HDL-chol, non-HLD-chol, education level, smoking	alpha-carotene: 0 - >9 µg/dL (0 - >0.168 µM) mean was 0.089±0.0017 µM for total cohort. For men = 0.0786±.0019; for women = 0.0989±0.0020 µM<br>beta-carotene levels were from ~0.15 - 0.85 µM<br>From Ford 2003 total carotenoids quartile cutoffs: 0.8979 1.309 1.649 µM	Compared to lowest quintile, All cause RR=0.61 (0.51-0.73);<br>CVD RR=0.71 (0.55-0.91);<br>Cancer RR=0.57 (0.34-0.96);<br>Diabetes RR=0.33 (0.13-0.82);<br>Chronic lower respiratory disease RR=0.14 (0.06-0.36)	The 9 µg/dl level is 0.168 µM. This is a terribly low number.

Study, N=111	Study, Country	Study Type	% female	Age range	Cohort Size	Follow-up, years	Cases (and controls, if applicable)	Adjustments	Range of Carotenoids	Effect Size, (95% CI) from lowest to highest	Comments
Mayne 2004	Carotene Prevention Trial, A Chemopreve ntion trial, USA, Yale & U of Miami	part of RCT	18	~62 avg	259	7.5	61	Age, sex, total chol, smoking, treatment arm, study site	Mean ± SD<br>beta-carotene = 0.335±0.39 μM<br>alpha-carotene = 0.06±0.056 μM<br>lutein/zeaxanthin = 0.26~0.29±0.14 μM<br>totalcarotenoids, surv 1.33±0.72, died 1.15±0.69μM total carotenoids wt avg 1.29±0.71 μM<br>lycopene, surv. = 0.63±0.39 μM<br>lycopene, died = 0.50±0.34 μM<br>mean lycopene = 0.52 μM	Whole group lycopene above vs. below median conc. HR=0.53 (0.30-0.930; for CV death alpha-carotene HR=0.24 (0.08-0.75) Non-smokers only alpha-carotene HR=0.25 (009-0.73); lycopene HR=0.08 (0.02-0.36); total carotenoids HR=.22 (0.07-0.70)	Among non-smokers there are some very significant trends. Half of smokers dead and half of non-smokers alive
Ray 2006	Women's Health and Aging Study I and II, Baltimore, MD, USA	Prosp. Cohort	100	70-79	632	5	14.1% (89 cases)	Age, education, smoking, BMI, poor appetite, diabetes, CKD, renal disease	total carotenoids (median, min, max) = 1.67 (0.13, 9.10) μM<br>cut-offs for quartiles = <1.038, 1.452, >1.995 μM	HR=0.77 (0.64-0.84 / 1 SD increase in loge total carotenoids)	Selenium also found to be protective in this cohort. One of first studies to find protective effects specifically in women.
Sahyoun 1996	Nutrition Status Survey, Mass., USA	Prosp. Cohort	65	60+	725	9-12 y	70 men, 127 women	Age, sex, cholesterol, Trends not signif. After adjusting for existing disease, disabilities affecting shopping, and plasma vit C and E.	Men (mean±SD) 2.39±0.91 μM<br>Women 2.53±0.97 μM<br>20th percentile 1.70; 80th 3.08 μM	Compare to lowest quintile, RR=0.67 (0.46-0.98) middle quintile, RR=0.56 (0.32-0.97) for highest quintile, when adjusted for age, sex, cholesterol	Vitamin C levels were the main reported effector. Carotenoids not independent from vitamin C, which makes sense.
Shardell 2011	NHANES III follow-up study, USA	Prosp. Cohort	52	≥20	13293	14.3 y	2,933 (1,264 CVD, 645 cancer)	Age, sex, race/ethnicity, marital status, education, alcohol, smoking, multivitamin use, physical activity, BMI, BP, total cholesterol, HDL-cholesterol, CRP, use of BP lowering medication, use of cholesterol-lowering medication, congestive heart failure, cancer, diabetes, emphysema, stroke	Total carotenoid quartile cutoffs 1.01, 1.33, 1.75 μM, individual carotenoid quartiles given in text.	RR=1.38 (1.15-1.65) p=0.005 for total carotenoid, low vs high	a-carotene also signif. related to all cause mortality and CVD death. No levels of carotenoids protective from cancer death. Seems to be a threshold effect in this study, too, with only the lowest quartile having significant higher mortality rates compared to the other 3 quartiles.
**Disability / Frailty / Cognition, N=7**											
Akbaraly 2007	Epidemiology of Vascular Aging, France	Cross-Section	61.3	68-79	589	0	NA	Age, sex, education, smoking, alcohol, medicine use, BMI, diabetes, hypertension	25, 50, 75th percentiles, μM<br>lutein: 0.176, 0.235, 0.335<br>zeaxanthin: 0.02, 0.029, 0.042<br>lycopene: 0.160, 0.257, 0.403<br>a-carotene: 0.098, 0.154, 0.245<br>trans-b-carotene: 0.374, 0.607, 0.953<br>total : 1.16, 1.65, ≥2.35 μM	Lower functioning had higher probability of lower levels of lycopene and zeaxanthin. Zeaxanthin OR are DSS=1.97 (1.21-3.20), FTT=1.70 (1.05-2.74), WFT=1.82 (1.08-3.07) lycopene OR DSS=1.93 (1.20-3.12), TMTB=1.64 (1.04-2.59)	plasma carotenoids highly and significantly correlated Diabetes assoc. with low levels of carotenoids, hypertension was assoc. with low levelsof lutein, a-carotene, trans- and cis-B-carotene
Alipanah 2009		Prospectiv e	100	65+	687	3		Age, BMI, chronic disease	Mean 2.08±0.95 μM		mean total serum carotenoids assoc with mean walking speed (p=0.0003) and rate of change of walking speed (p=0.007).
Hu 2006	MacArthur Stdies of Successful Aging, USA	Prosp. Cohort	59.3	74±2.7	455 survivo rs	7		Age, sex, race, education, BMI, baseline SPMSQ score, education, income, smoking status, alcohol, CRP, IL-6, total chol, HDL-chol	mean b-carotene = 0.27±0.31μM	OR for mental decline (SPMSQ scores)<br>APOE 4 positive: 0.11 (0.02-0.57)<br>APOE4 negative: 0.89 (0.54-1.47)	
Michelon 2006	Women's Health and Aging Study I and II, Baltimore, MD, USA	Cross-Section	100	70-79	754	5		Age, sociodemographic status, smoking, BMI	Mean total carotenoids for nonfrail, pre-frail, and frail women were 1.842. 1.593, and 1.376 μM	OR (from 1.82 to 2.45) for fraility were higher for women with micronutrient conc. In lowest quartile compared to top 3 quartiles	Frail women tend to have at least 2 micronutrient deficiencies. Associations strongest for b-carotene, luteinzeaxanthin, and total carotenoids.

Study, N=111	Study, Country	Study Type	% female	Age range	Cohort Size	Follow-up, years	Cases (and controls, if applicable)	Adjustments	Range of Carotenoids	Effect Size, (95% CI) from lowest to highest	Comments
Semba 2003	Women's Health and Aging Studies, Baltimore, MD, USA	Cross-Section	100	70-79	696			Age, race, smoking, CVD, arthritis, IL-6	close to rest of this Woman's Health and Aging study.	highest compared to lowest, total carotenoids grip strength OR=0.34 (0.20-0.59), hip strength OR=0.28 (0.16-0.48), knee strength OR=0.45 (0.27-0.75)	
Semba 2006	Women's Health and Aging Study I, Baltimore, MD, USA	Prosp. Cohort	100	65+	766	3	31.9% (205 of 463 w/ at least one follow-up visit	Age, smoking, chronic pulmonary disease, others?	close to rest of this Woman's Health and Aging study.	lowest compared to highest quartile total carotenoid HR=1.54 (1.11-2.13)	
Yang 2008	USA	Cross-Section	100	62.7±8.8	59			Serum retinol, supplement usage, milk, yogurt, fruit, and vegetable intake, BMI	5-95th percentile<br>Lutein (0.18 - 1.25)<br>b-cryptoxanthin (0.08 - 0.87)<br>lycopene (0.47 - 3.98)<br>a-carotene (0.07 - 0.79)<br>b-carotene (0.20 - 4.20)<br>Total mean = 4.22±1.73 µM (1.0-11.09)	Lycopene and b-cryptoxanthin lower in osteoporosis group, T-test used.	Mean serum lycopene, osteo group 1.22±1.21 µM, median 0.93 µM and control group 1.97±1.01 µM, median 1.83 µM. Mean serum b-cryptoxanthin osteo group = 0.27±0.18 µM, median 0.24 µM, and control group [0.43±0.36 µM, median 0.34 µM<br>Overall carotenoid concentrations higher than in Framingham study or in NHANES III cohorts.
**Cardiovascular / Carotid media intima / hypertension, N=11**											
Buijise 2005	SENECA, Survey in Europe on Nutrition and the Elderly, a Concerted Action, Europe	Prosp. Cohort	50	70-75	1168	10	388	age, sex, BMI, cholesterol, HDL cholesterol, smoking, alcohol, physical activity, SENECA center, alpha-tocopherol	Tertiles of carotenes, X±SD, µM: 0.22±0.12; 0.54±0.09; 1.12±0.39	for increment of 0.39 µM carotenes, RR 0.79 (0.70, 0.89) total mortality	cancer: RR 0.59 (0.44, 0.79) CVD: RR 0.83 (0.70. 1.00), lower risk of CVD only for those w/ BMI<25; for those w/ BMI <25 RR=0.67 (0.49, 0.94). More men with low carotenes: 70%, 47%, and 34% men in low, mid, and high tertile of carotenes.
D'Odorico	Bruneck study, Italy	Prosp. Cohort	47.7	45-65	392	5	all	Age, sex, LDL, ferritin, systolic BP, smoking, alcohol, social status, CRP	Varies by Age Category, approximates<br>Lutein: M ~0.34, W ~0.37 µM<br>Zeaxanthin: M ~0.10, W ~0.10 µM<br>Cryptoxanthin: M ~0.23, W ~0.36 µM<br>Lycopene: M ~0.55, W ~0.65 µM<br>a-carotene: M ~0.11, W ~0.15 µM<br>b-carotene: M ~0.56, W ~1.05 µM<br>Total: M 1.98±0.81, W 2.78±1.32 µM (adjusted)	OR of atherosclerosis for Q5 (>1.30 µM a+b-carotene) vs Q1 (<0.40) = 0.2 (~0.05 - 0.55)	a-carotene and b-carotene inversely assoc. with prevalence of atherosclerotic lesions and 5-year incidence of atherosclerotic lesions in carotid arteries. See graph next to this. Only top quintile signif. different from bottom quartile.
Dwyer 2001	Los Angeles Atherosclerosis Study	Prosp. Cohort	47	40-60	480	1.5	all	Age, sex, ethnicity, time between exams, smoking, systolic BP, total chol, HDL chol, plasma b-carotene, BMI, alcohol, hours since last meal at blood draw, history of diabetes, current meds for diabetes or hypertension, interaction between lutein and meds	Mean±SD Lutein: 0.28±0.12 µM<br>b-carotene 0.74±0.80 µM<br>Range of lutein quintiles: Women, 0.070-0.182, 0.240, 0.296, 0.367-0.805; Men: 0.019-0.180, 0.230, 0.280, 0.350-0.790 µM	IMT declined for highest vs lowest quintile lutein 0.004±0.005mm high, 0.021±0.005mm low (±SEM)	

Study, N=111	Study, Country	Study Type	% female	Age range	Cohort Size	Follow-up, years	Cases (and controls, if applicable)	Adjustments	Range of Carotenoids	Effect Size, (95% CI) from lowest to highest	Comments
Dwyer 2004	Los Angeles Atherosclerosis Study	Prosp. Cohort	46	40-60	573	1.5	all	Age, sex, BMI, smoking, total chol, HDL-C, systolic BP, current meds for hypertension and elevated cholesterol, history of diabetes, ethviticy, alcohol, height, CRP, time between exams	Antioxidant quintile ranges (µmol/L): lutein: 0.02 to 0.18, 0.18 to 0.23, 0.24 to 0.29, 0.29 to 0.36, 0.36 to 0.81; zeaxanthin: 0.01 to 0.04, 0.04 to 0.05, 0.05 to 0.06, 0.06 to 0.08, 0.09 to 0.28; b-cryptoxanthin: 0.01 to 0.04, 0.05 to 0.06, 0.06 to 0.08, 0.08 to 0.12, 0.12 to 0.60; a-carotene: 0.01 to 0.07, 0.07 to 0.11, 0.11 to 0.15, 0.16 to 0.27, 0.27 to 1.06; b-carotene: 0.02 to 0.24, 0.24 to 0.42, 0.42 to 0.64, 0.64 to 1.04, 1.05 to 8.00; and lycopene: 0.03 to 0.31, 0.31 to 0.47, 0.47 to 0.67, 0.67 to 1.02, 1.03 to 6.47	higher plasma levels of lutein (P=0.017), zeaxanthin (P=0.0004),b-cryptoxanthin (P=0.015), and a-carotene (P=0.003) were associated with reduced progression of carotid IMT. On average, for every 1 µmol/L increase of plasma lutein, zeaxanthin, b-cryptoxanthin, or a-carotene, IMT progression was reduced by 3.2, 4.7, 3.4, and 4.2 µm/18 months, respectively.	
Hak 2003	Physicians' Health Study, USA	nested case-control	0	40-84 58±8.5	1,062	13	531 (531)	Age, smoking, BMI, total chol, HDL-C, history of hypertension, diabetes, parental MI before age 60, freq of vigorous exercise, alcohol, multivitamin, assignment to aspirin, b-carotene, or placebo	Median of quintiles (lowest and highest)<br>a-carotene: 0.057-0.198 µM<br>b-carotene: 0.207-0.888 µM<br>b-cryptoxanthin: 0.046-0.233 µM<br>lutein: 0.192-0.517 µM<br>lycopene: 0.405-1.078 µM	No protective effect seen, even in model only adjusted for age and smoking.	14.7% smokers, 40.9% former smokers, 44.4% never smoked in cases and controls
Hozawa 2009	CARDIA (Coronary Artery Risk Development in Young Adults), USA	Prosp. Cohort	54.9	18-30 24.8±3.6	4,412	20	all	Age, sex, race, center, education, yr0 alcohol, total chol, HDL-C, triglycerides, energy intake, BMI, smoking, physical activity, vitamin suppl., yr0 systolic BP	Sum of 4 carotenoids acress quartiles:<br>Q1: 0.010 - 0.053 µM<br>Q2: 0.053 - 0.074 µM<br>Q3: 0.074 - 0.1015 µM<br>Q4: 0.1015 - 0.579 µM<br>Lycopene: ~ 0.055 (yr0) to 0.079 (yr15)	Fully adjusted Q4 vs Q1 RH=0.86 (0.70-1.06). W/o yr0 BP, RH=0.78 (0.63-0.96) RH for hypertension, time dependent model, for 1 SD increase in sum of 4 carotenoids RH=0.83 (0.76-0.90), model 2 w/o yr1 BP	Amounts of carotenoids are all low in this cohort. Top of Q4 is 0.58 µM, which is still in the dangerous range. All of these numbers are 10-fold lower than normal and 10-fold lower than numbers in the Hozawa 2006 report. Adjusted to be equivalent in the published article.
Hozawa 2007	CARDIA (Coronary Artery Risk Development in Young Adults), USA	Prosp. Cohort	55	18-30 24.8±3.6	4580	15	all	Age, sex, race, center, education, energy intake, alcohol, BMI, physical activity, LDL-C, systolic BP, use of vitamin suppl. (A, C, or E), smoking, HDL-C, triglycerides	Range of sum of 4 carotenoids: 0.010 - 0.0513, 0.0513-0.0716, 0.0716-0.0979, 0.0979-0.5749 µM<br>Lycopene range from 0.0012 to 0.1779 µM	No RR given, just correlations. The year 0 sum of 4 carotenoids was inversely associated (all P <0.05) with year 0 leukocyte count (slope per sum carotenoid SD, -0.17); year 7 fibrinogen (slope, -0.10); year 7 and year 15 C-reactive protein (slope, -0.12 and -0.09); and year 15 F2-isoprostanes (slope, -13.0), soluble P-selectin (slope, -0.48), and soluble intercellular adhesion molecule-1 (sICAM1; slope, -5.1).	Leukocyte counts and sICAM1 and F2-isoprostane concentrations had stronger associations in smokers than in nonsmokers, and sICAM1 concentrations were higher in the highest carotenoid quartile in smokers than in the lowest carotenoid quartile in nonsmokers. Superoxide dismutase was positively associated with the sum of 4 carotenoids (slope, 0.12; P <0.01). Lycopene was inversely associated only with sICAM1. The year 7 carotenoid associations with these markers were mostly similar to those at year 0.
Ito 2006a	Hokkaido, Japan	Prosp. Cohort	61.1	39-80	3061	11.9	80 (49 M, 31 W), 40 from heart disease, 37 from stroke	Age, sex, BMI, total chol, smoking, alcohol, systolic BP, triglyceride, ALT activity	For those alive at end of study, other values slightly lower<br>a-carotene: M: 0.106, W: 0.166 µM<br>b-carotene: M: 0.425, W: 0.969 µM<br>lycopene: M: 0.258, W: 0.371 µM<br>total carotene: M: 0.848, W: 1.587 µM	Cardiovascular disease, all: a-carotene HR=0.55 (0.38-0.79) b-carotene HR=0.64 (0.47-0.89)<br>Heart disease: a-carotene HR=0.51 (0.30-0.86), b-carotene HR=0.84 (0.53-1.34)<br>Stroke: a-carotene HR=0.52 90.31-0.88), b-carotene HR=0.48 (0.31-0.76)	Only a-carotene and b-carotene are related strongly with reduced risk. Lycopene attenuates the total carotene score towards a null finding.

Study, N=111	Study, Country	Study Type	% female	Age range	Cohort Size	Follow-up, years	Cases (and controls, if applicable)	Adjustments	Range of Carotenoids	Effect Size, (95% CI) from lowest to highest	Comments
Ito 2006b	Hokkaido, Japan	Prosp. Cohort	61.3	39-85	3254	11.7	140 (86 M, 54 W) cancer deaths, (41 lung, 17 stomach, 16 colorectal, 12 liver) 89 cardiovascular disease (45 heart, 37 stroke)	Age, sex, smoking, alcohol, total chol, triglycerides, ALT activity (cancer) For CVD death BMI, systolic BP added	Geometric mean values for those alive a-carotene: 0.121 µM b-carotene 0.727 µM; lycopene 0.289 µM; b-cryptoxanthin 0.327 µM; zeaxanthin/lutein 1.064 µM, Total Carotenoids 2.821 µM	**All Cause Mortality:** a-carotene HR=0.79 (0.65-0.95), b-carotene HR=0.81 (0.69-0.95), lycopene HR=0.80 (0.69-0.93), b-cryptxanthin HR=.84 (0.71-0.99); zeaxanthin/lutein HR=0.78 (0.63-0.95); Total carotenoids HR=0.69 (0.55-0.87) **Cancer Mortality:** All sites a-carotene HR=0.71 (0.54-0.94), b-carotene HR=0.76 (0.60-0.96), lycopene HR=0.72 (0.57-0.90), zeaxanthin/lutein HR=0.67 (0.49-0.91), total carotenoids HR=0.61 (0.43-0.86). Individual signif corr for b-carotene and colorectal and liver, lycopene for colorectal, total carotenoids for colorectal cancer. **Cardiovascular Mortality:** a-carotene HR=0.71 (0.49-1.04), b-carotene HR=0.71 (0.51-0.99), total carotenoid HR=0.62 (0.38-1.01)P=0.054. Stroke, total carotenoid HR=0.40 (0.24-1.05)P=.067	Total carotenoids signif lower, alive compared to dead, for all cause mortality, all cancer sites, lung, stomach, and liver, but not colorectal. And signif. Lower in stroke death.
Rissanen 2003	Kuopio Ischaemic Heart Disease Risk Factor Study, eastern Finland	Cross-section, population based	0	46-64	1028		all	age, ultrasound observer, examination years, systolic BP, HDL-C, LDL-C, smoking, triacylglycerols, BMI, serum folate, b-carotene, alpha-tocopherol	Lycopene quartiles: <0.04, 0.04-0.13, 0.14-0.22, >0.22 µM; range from below detectable limit to 1.02 µM. Mean wal 0.15±0.14 µM.	Significant inverse association between serum lycopene and mean and maximal IMT, P=0.005 and P=0.001, respect.	
Yanagiswa 2009	Japan	Cross-section	68	39-70	625	0	all			TT genotype of IL-1beta C-31T OR=1.82 (1.09-3.06) for hypertension. Low b-carotene and TT genotype OR, males=5.03 (1.34-21.58), women OR=2.47 (1.04-6.00). Male with CC genotype (negative for risk) with high b-carotene OR=0.25 (0.06-0.95) for hypertension.	gene polymorphism and beta-carotene interaction
**Metabolic Syndrome, N=6**											
Beydoun	NHANES 2001-2006, USA	Cross-section, population based	51	20-85	3008 - 9009	0	all	Age, sex, race/ethnicity, marital status, education level, PIR (poverty income ratio), smoking, physical activity, dietary intakes (total energy, alcohol, caffeine, b-carotene, vit C, vit E, diet suppl. Use), serum levels of folate, tHcy, vit B-12 25(OH)D, total chol., triglycerides	Total carotenoids quartiles, n=1574: 0.057-0.863, 0.863-1.183, 1.183-1.622, 1.62-10.114 µM	n=3008, total carotenoids, **per 1-SD increase (0.73µM), OR of MetS+ = 0.67 (0.58, 0.77)**. About same for men and women. n=4218, **Total Carotenoids** OR, abdominal obesity = 0.58 (0.51, 0.65); Hypertriglyceridemia OR= 0.39 (0.34-0.45); Low HDL-C OR=0.65 (0.55-0.77); high BP OR=0.78 (0.68-0.90); hyperglycemia OR=0.80 (0.70-0.92); Elevatie HOMA-IR OR=0.62 (0.55-0.70); elevated CRP OR=0.35 (0.22-0.55); hyperuricemia OR=0.58 (0.46-0.74)	32% of men and 29.5% of women had metabolic syndrome in this sample. Shows that an increase of just 0.73 µM has a significant impact on health.

Study, N=111	Study, Country	Study Type	% female	Age range	Cohort Size	Follow-up, years	Cases (and controls, if applicable)	Adjustments	Range of Carotenoids	Effect Size, (95% CI) from lowest to highest	Comments
Coyne 2009	Australian Diabetes, Obesity and Lifestyle Study, Australia	Cross-section	57.9	25+	1523	0	all	Age, sex, educationb BMI, alcohol, smoking, physical activity, vitamin/mineral use	Adj. geometric means w/ and w/o MetS,µM<br>a-carotene: 0.10 (0.07-0.15); 0.13 (0.10-0.18)<br>b-carotene: 0.45 (0.33-0.62); 0.59 (0.47-0.73)<br>b-cryptoxanthin: 0.20 (0.16-0.24); 0.21 (0.18-0.25)<br>lutein/zeaxanthin: 0.41 (0.36-0.46); 0.41 (0.34-0.50)<br>lycopene: 0.40 (0.34-0.47); 0.43 (0.39-0.49)<br>total carotenoids: 1.82 (1.62-2.04); 2.02 (1.76-2.33)	OR for metabolic syndrome:<br>a-carotene Q1 vs Q4 OR=0.36 (0.21-0.63)P,trend=0.005<br>b-carotene Q1 vs Q4 OR=0.36 (0.24-0.55) P,trend=0.003<br>Total carotenoids Q1 vs Q4 OR=0.43 (0.23-0.79) P,trend=0.04<br>Also signif OR for abdominal obesity and a-carotene, b-carotene and total carotenoids, high triglycerides and a- b-carotene, high BP and a- and b-carotene and total carotenoids	Prevalence of metabolic syndrome was 24%. Relationship particularly strong for a-carotene and b-carotene
Czernichow 2009	SU.VI.MAX, France	RCT, baseline data	62	49±6.2	5220	7.5	263 MetS+	Age, sex, education, smoking, physical activity, alcohol, triglycerides, total chol.	b-carotene only: medians of tertiles, 0.29, 0.52, 0.91 µM	Compare lowest to highest tertile of b-carotene: OR=0.34 (0.21-0.53), for 2nd to lowest tertlie of b-carotene: OR=0.55 (0.38-0.80), fully adjusted	supplemental b-carotene did not prevent MetS, but baseline b-carotene did predict it quite well.
Ford 2003	NHANES III (1988-1994)	Cross-section, population based	50.4	20+	8808	0	2268 MetS+ (23% age adjusted rate)	Age, sex, race/ethnicity, education, smoking, cotinine conc., physical activity, fruit and vegetable intake, vitamin or mineral use, serum lipids	MetS+ avg total carotenoids = 1.29±0.02, MetS- avg total carotenoids = 1.51±0.02 µM<br>Avg conc. From 0 to 5 MetS criteria (0, then 5)<br>a-carotene: 0.11±0.00; 0.05±0.00<br>b-carotene: 0.47±0.02; 0.22±0.01<br>b-cryptoxanthin: 0.18±0.00; 0.15±0.00<br>lutein/zeaxanthin: 0.41±0.01; 0.34±0.02<br>lycopene: 0.44±0.01; 0.40±0.02 (not signif diff.)<br>**Total carotenoids: 1.61±0.02; 1.17±0.04**<br>Range of carotenoids in Cohort in other publication	No effect sizes given. Significantly lower carotenoids among subjects with MetS for all but lycopene.	
Sugiura 2008	Mikkabi, Shizuoka Prefecture, Japan,	Cross-section, population based	66.7	30-70	1073	0	59 MetS+	Age, sex, alcohol, exercise habits, energy intake	Highest range is non-smokers, MetS-, µM all<br>lycopene: 0.29 (0.28-0.31), 0.19 (0.12-0.30)<br>a-carotene: 0.14 (0.13-0.14), 0.07 (0.05-0.09)<br>b-carotene: 0.66 (0.64-0.69), 0.20 (0.14-0.29)<br>lutein: 0.56 (0.55-0.58), 0.45 (0.35-0.57)<br>b-cryptoxanthin: 1.39 (1.31-1.48), 0.44 (0.27-0.72)<br>zeaxanithin: 0.23 (0.22-0.23), 0.22 (0.18-0.26)<br>Total carotenoids: 3.27, 1.57	serum b-carotene, high vs low tertile OR=0.41 (0.18-0.92)<br>in current smokers middle tertile OR=0.10 (0.01-0.72) and highest tertile OR=0.06 (0.01-0.73), in curr smokers a-carotene and b-cryptoxanthin signif low OR.<br>In non-smokers highest tertile b-carotene OR=0.30 (0.10-0.89)	Rates of MetS were 5.6 and 10.7% in non-smokers and smokers. Much lower than western populations.
Suzuki 2011	Japan	Cross-section, population based	65.8	39-70	931	0	Men 71 (247); Women 46 (567)	age, sex, smoking, alcohol, serum total cholesterol	Geometric mean (25-75 percentile) Also tertiles for men and women. Individual carotenoids summed and adjusted.<br>Men, controls: 2.728 (2.054 3.482); Met syn 2.464 (1.894 3.337) Women, controls: 4.081 (3.087 5.160) met syn 3.465 (2.669 4.355) µM	Highest vs lowest tertile. B-cryptoxanthin OR=0.45 (0.22-0.93) in men and OR=0.41 (0.17-0.93) in women. B-carotene OR, men=0.45 (0.21-0.95); OR,women=0.37 (0.15-0.83). No signif diff found in current male smokers. In women zeaxanthin/lutein OR=0.37 (0.16-0.84), high vs low tertile. Carotenoids decreased as number of MetS components increased.	
**Inflammation / Antiox Measures, N=6**											
Collins 1998	Spain	Cross-section	50	25-45	32	0		Not many	Not given	significant negative correlation between basal conc. Of total serum carotenoids and oxidized pyrimidines, and also for lutein and b-carotene.	suppl. b-carotene did not reduce oxidative damage measured by Comet assay

Study, N=111	Study, Country	Study Type	% female	Age range	Cohort Size	Follow-up, years	Cases (and controls, if applicable)	Adjustments	Range of Carotenoids	Effect Size, (95% CI) from lowest to highest	Comments
Ford 2003 (CRP)	NHANES III, USA	Cross-section, population based	~50%	20+	14,519	0		Age, sex, race/ethnicity, education, cotinine conc., BMI, leisure physical activity, aspirin use	a-Carotene: quartile 1, ≤0.02; q2, 0.03–0.06; q3, 0.07–0.09; q4, >0.09. b-Carotene: q1, ≤0.15; q2, 0.16–0.26; q3, 0.27–0.43; q4, >0.43. b-Cryptoxanthin: q1, ≤0.07; q2, 0.08–0.11; q3, 0.12–0.18; q4, >0.18. Lutein/zeaxanthin: q1, ≤0.23; q2, 0.24–0.32; q3, 0.33–0.46; q4, >0.46. Lycopene: q1, ≤0.26; q2, 0.27–0.39; q3, 0.40–0.54; q4, >0.54.	OR for high CRP, highest vs lowest quartile. a-carotene: 0.41 (0.33, 0.50), b-carotene: 0.39 (0.31, 0.48), b-cryptoxanthin: 0.50 (0.40, 0.63), lutein/zeaxanthin: 0.70 (0.54-0.89), lycopene: 0.58 (0.49-0.70)	
Ford 2005	NHANES III, USA	Cross-section, population based	~50%	20+	9575	0		age, sex, race or ethnicity, education, smoking status, cotinine concentration, physical activity, alcohol use, fruit and vegetable intake, vitamin or mineral use during the past 24 hours, body mass index, systolic blood pressure, and total cholesterol, triglyceride, glucose, insulin, and C-reactive protein concentrations	same as rest of NHANES III	OR for microalbuminuria, highest vs lowest quartile. B-cryptoxanthin: OR=0.56 (0.38-0.82), lutein/zeaxanthin OR=0.59 (0.37-0.94), lycopene OR=0.64 (0.46-0.89), Total carotenoids OR=0.54 (0.38-0.75)	Vit C, E, and selenium in this population not related to microalbuminuria.
Hughes 2009	USA, from patients of "curative" early stage head and neck cancer)	Cross-section	27		52			smoking status, alcohol consumption, age, dietary vitamin C, plasma alpha-tocopherol, sex, BMI and education	Median values (25th:75th quartile)<br>Plasma alpha-carotene 0.048 (0.028:0.097)<br>Plasma beta-carotene 0.23 (0.10:0.45)<br>Plasma lutein 0.16 (0.11:0.22)<br>Plasma zeaxanthin 0.049 (0.033:0.066)<br>Plasma lycopene 0.30 (0.18:0.47)<br>Plasma beta-cryptoxanthin 0.071 (0.044:0.13)<br>Total plasma carotenoids 0.92 (0.63:1.51)	unadjusted signif diff in plasma lutein and both F2-IsoP measures. Not signif after adjustments.	Almost a null study.
Rao 2006	Canada	Cross-section	100	50-60	33	0				higher serum lycopene signif assoc with low NTx (P<0.005) and lower protein oxidation (p<0.05)	
Suzuki 2010	Japan	Cross-section	73.5	adults ? 39-70?	437	0		confounding factors (not listed in abstract)		serum b-carotene signif assoc w/ HMW adiponectin in men and women; serum a- & b-carotene signif assoc with CRP in men; women had signif neg assoc w/ carotenoids and IL-6.	
**Diabetes, N=11**											
Akbaraly 2008	Epidemiology of Vascular Aging, France	Prosp. Cohort	58.6	59-70	1035 (Table 3)	9	127 dysglycemia	Age, sex, education, smoking, alcohol, BMI, total and HDL-C, BP, use of lipid lowering medications, history of CVD	Q1 <1.82, Q2 1.82-2.55, Q3 2.55-3.43, Q4 ≥3.43 µM	Q4 vs Q1 HR=0.42 (0.22-0.82) P=0.01	Graded effect, with no low threshold as seen with all-cause mortality studies. And highest quartile is ≥3.43 µM. Important to note this.
Arnlov	Uppsala Longi	Prosp. Cohort	0	50	846	27	245 diabetics	BMI, physical activity, smoking, HOMA insulin sensitivity index, acute insulin response, impaired fasting blood glucose	highest tertile b-carotene >0.335 µM	59% lower risk of diabetes in highest tertile of serum b-carotene (>0.335 µM) HR=0.41 (0.23-0.74); in the middle tertile there was a significant diff also HR=0.54 (0.32-0.92). In continous model for 1-SD inccrease in serum b-carotene there was a 32% lower risk of diabetes HR = 0.68 (0.53-0.89)	

Study, N=111	Study, Country	Study Type	% female	Age range	Cohort Size	Follow-up, years	Cases (and controls, if applicable)	Adjustments	Range of Carotenoids	Effect Size, (95% CI) from lowest to highest	Comments
Coyne 2005	Australian Diabetes, Oxesity and lifestyle Study, Australia	Cross-section	57.5	≥25	1597	0	132 diabetic, 320 impaired glucose metabolism, 1,145 normal	Age; sex; BMI; physical activity; educational status; smoking; alcohol intake; vitamin use; total, HDL, and LDL cholesterol; triacylglycerol; and systolic and diastolic blood pressures	Median values of quintiles. a-carotene: Q1 (0.04) Q2 (0.08) Q3 (0.13) Q4 (0.21) Q5 (0.39). B-carotene: Q1 (0.17) Q2 (0.36) Q3 (0.55) Q4 (0.83) Q5 (1.50). B-cryptoxanthin: Q1 (0.07) Q2 (0.13) Q3 (0.21) Q4 (0.33) Q5 (0.66). Lutein/zeaxanthin: Q1 (0.20) Q2 (0.32) Q3 (0.42) Q4 (0.56) Q5 (0.82). Lycopene: Q1 (0.17) Q2 (0.33) Q3 (0.46) Q4 (0.65) Q5 (0.98).	Mean 2-h postload plasma glucose and fasting insulin concentrations decreased significantly with increasing quintiles of the 5 serum carotenoids—α-carotene, ß-carotene, ß-cryptoxanthin, lutein/zeaxanthin, and lycopene. Fasting glucose decreased significantly with increasing quintiles of a-carotene and b-carotene. Geometric mean concentrations for all serum carotenoids decreased (all decreases were significant except that of lycopene) with declining glucose tolerance status. Sum of total carotenoids was 1.80, 1.60, and 1.41 µM for normal, impaired, and diabetic (OGT test).	Range is not as great as in Arnlov study, but results good.
Czernichow 2006	SU.VI.MAX, France	Prosp. Cohort	59.2	W 35-60, M 45-60	3146	7.5		age, sex, BMI, smoking status, physical activity, educational level, supplementation group, and energy intake	Geometric mean conc. Of b-carotene: W 0.59 (0.57-0.61); M 0.41 (0.39-0.43), placebo; M 0.39 (0.37-0.41)intervention group	Baseline ß-carotene dietary intakes and plasma concentrations were inversely associated with FPG in multivariate mixed models (P = 0.0045 and P < 0.0001, respectively).	Low concentrations of b-carotene
Ford 1999	NHANES III, USA	Cross-section, population based	47.7	40-74	1563 w/plasma carotenoid measures	0	Normal = 1,010 Impaired glucose tolerance = 277, newly diagnosed diabetic = 148, previous diagnosed diabetes = 230	age, sex, race, education, serum cotinine, serum cholesterol, body mass index, physical activity, alcohol consumption, vitamin use, and carotene and energy intake	Mean levels for normal group: a-carotene 0.098, b-carotene 0.425, cryptoxanthin 0.156, lutein/zeaxanthin 0.413, lycopene 0.415 geometric means of b-carotene for normal, impaired glucose tolerance, and diabtes: 0.363, 0.36. 0.290 µM for lycopene 0.277, 0.259, 0.231 µM	b-carotene and lycopene have signif inverse trends in relation to fasting blood glucose. All carotenoids inversely related to fasting blood insulin (as in Czernichow 2006)	
Hozawa 2006	CARDIA, USA	Prosp. Cohort	55.1	18-30	4493	15	148 diabetes	study center, race, sex, education, age, total energy intake, alcohol, BMI, phylical activity, total chol, HDL-C, systolic BP, triglyceride, use of vitamin suppl. (A, C, E, or b-carotene)	lowest quartile 0.16 - 0.98 µM, Q2 0.98-1.29, Q3 1.29-1.66, Q4 1.66-5.98 µM, individual quartiles of carotenoids on separate tab	Diabetes incidence was inversely associated with the sum of carotenoid concentrations in nonsmokers (per standard deviation (SD) increase, relative hazard = 0.74, 95% confidence interval: 0.55, 0.99) but not in current smokers. Similarly, year 15 insulin and insulin resistance values, adjusted for baseline levels, were inversely related to sum of carotenoids only in nonsmokers (per SD increase in insulin level, slope = -0.46 (p = 0.03); per SD increase in insulin resistance, slope = -0.14 (p = 0.01)).	relationships found in non-smokers, but not in smokers relationships would have been stronger if lycopene was excluded from their calculations, using the other 4 carotenoids instead by themselves.
Reunanen 1998	Finland	Nested case-control	48.2	59.9±9.5			106 (201)	sex, age, study region matched between cases and controls, full adjustment included total chol, obesity, smoking, hypertension		RR=0.45 (0.22-0.92) high vs low tertile of b-carotene, not fully adjusted. Fully adjusted RR=0.94 (0.38-2.32)	
Suzuki 2000	Japan	Cross-section								serum a- and b-carotene and cryptoxanthin lower and TBARS higher among those with high hbA1c newly detected, compared to those with normal levels	very few numerical details in abstract, full article in Japanese

Supplementary Materials

Study, N=111	Study, Country	Study Type	% female	Age range	Cohort Size	Follow-up, years	Cases (and controls, if applicable)	Adjustments	Range of Carotenoids	Effect Size, (95% CI) from lowest to highest	Comments
Suzuki 2002	Hokkaido, Japan	Case-control, population based	46.4 w/ high HbA1c; 45.1 with diabetes	63±10	1,691 examined from 1998 to 2000	0	151 (302) with high HbA1c; 133 (266) with diabetes	cases and controls matched for sex and age, logistic regression adjusted for smoking, alcohol, systolic BP, BMI, total chol, triglyceride, gamma-GTP activity	Lowest is the subjects with high HbA1c. Geometric mean (10-90% range) B-carotene: 0.87 (0.28-2.39) a-carotene: 0.10 (0.04-0.22), lycopene 0.38 (0.14-1.02), b-cryptoxanthin 0.20 (0.08-0.42), lutein/zeaxanthin 0.97 (0.56-1.63) Highest is normal subjects. B-carotene 1.24 (0.45-3.29), a-carotene 0.13 (0.05-0.31), lycopene 0.46 (0.17-1.23), b-cryptxanthin 0.26 (0.10-0.63), lutein/zeaxanthin 1.17 (0.62-1.58) Highest end of upper tertiles are b-carotene 8.66, a-carotene 2.63, lycopene 3.19, b-cryptoxanthin 1.91, lutein/zeaxanthin 6.67 µM	The ORs for high Hb1Ac on high serum levels of alpha- and beta-carotenes, lycopene, beta-cryptoxanthin and zeaxanthin and lutein were 0.51 (0.27-0.96), 0.36 (0.22-0.80), 0.589 0.35-0.99), 0.41 (0.22-0.79), 0.37 (0.26-0.60) for high HbA1c, respectively. Not signif related to incidence of diabetes.	Differences in carotenoid levels for diabetics and healthy controls were very small in this case-control study.
Wang 2006	Women's Health Study, USA	Nested case-control	100	≥45		10	470 (470)	age, total chol, randomized treatment assignment, smoking, alcohol, vigorous exercise, menopausal status, postmenopausal hormone use, multivitamin use, family history of diabetes, BMI, history of hypertension, hypercholesterolemia, total energy intake, intake of energy-adjusted saturated fat, fiber, and glycemic load	Median values of quartiles, µM a-carotene Q1 0.039, Q2 0.093, Q3 0.158, Q4 0.39; b-carotene Q1 0.170, Q2 0.326, Q3 0.494, Q4 0.907; lutein/zeaxanthin Q1 .0176, Q2 0.264, Q3 0.350, Q4 0.501; b-cryptoxanthin Q1 0.069, Q2 0.137, Q3 0.221, Q4 0.374; lycopene Q1 0.209, Q2 0.335, Q3 0.443, Q4 0.590	highest versus the lowest quartile of plasma carotenoids were 1.13 (95% confidence interval (CI): 0.60, 2.13) for lycopene, 1.27 (95% CI: 0.63, 2.57) for α-carotene, 1.10 (95% CI: 0.57, 2.13) for β-carotene, 0.91 (95% CI: 0.46, 1.81) for β-cryptoxanthin, and 1.35 (95% CI: 0.68, 2.69) for lutein/zeaxanthin.	
Ylonen 2003	Botnia study, western Finland	Cross-section	55.5	53±9	182			Age, BMI, waist-to-hip ratio, physical activity, smoking, education, intake of polyunsaturated fat, dietary fiber, alcohol	Mean ±SD. A-carotene M 0.12±0.11, W 0.20±0.16; b-carotene M 0.51±0.30, W 0.73±0.42; lycopene M 0.31±0.24, W 0.30±0.18 µM	In men plasma ß-carotene concentrations were inversely associated with insulin resistance (P = 0.003), plasma ß-carotene concentrations were inversely and directly associated, respectively, with fasting plasma glucose concentrations (P < 0.05).	
**Lung Cancer, N=13 (1 more is a repeat entry)**											
Alfonso 2006 (data from Alfonso 2005	western Australia	Prospective cohort			1953	yes	65 (mesothelioma) 47 (lung cancer)			Plasma carotene concentrations at the first measurement, but not during the follow-up period, were associated with lower incidence of lung cancer in men and in workers.	
Comstock 1991 (same cohort as 1997)	Maryland, USA	Nested case-control			25,802		436 cancer (765)	Age, race, sex, month blood was drawn and time between last meal and blood draw all matched in cases and controls	Cancer cases, median serum b-carotene ~0.33 - 0.73 µM. Serum lycopene ~0.37-0.78 µM	Lycopene, OR,pancreas = 5.4 (low vs high, p=0.01). OR,bladder lycopene OR=2.0 (p=0.06). OR,lung b-carotene = 2.2 (p=0.04)	Not sure that the results were adjusted for smoking, alcohol, cholesterol levels, BMI, and other confounding factors. Squamous cell lung cancer was only subtype of lung cancer that had high OR (4.3)
Comstock 1997	Maryland, USA	Nested case-control	39.2				M 157 lung (313); W 101 (202)	Age, race, sex, months in which blood was drawn, and program of participation (1989 or 1974). Smoking not controlled for because authors argue that this is really a partial dietary adjustment.	Mean values, control then case. A-carotene 0.057, 0.049 b-carotene 0.294, 0.244 cryptoxanthin 0.132, .098 lutein / zeaxanthin 0.364, 0.327 lycopene 0.643, .635 Total: 1.490, 1.353 µM	OR for lung cancer, highest quintile vs lowest. A-carotene OR=0.48 (p=0.01), b-carotene OR=0.44 (p=0.002), cryptoxanthin OR=0.29 (p<0.001), lut/zea OR=0.41 (p=<0.0010	

Study, N=111	Study, Country	Study Type	% female	Age range	Cohort Size	Follow-up, years	Cases (and controls, if applicable)	Adjustments	Range of Carotenoids	Effect Size, (95% CI) from lowest to highest	Comments
Connet 1989	Multiple Risk factor Intervention Trial (MRFIT)	Nested case-control	0	35-57	12,866	10	156 (311) 66 lung	Age, smoking, randomization group, date of randomization, clinical center, further adjusted for # cigarettes, alcohol, serum thiocyanate levels, cholesterol levels (also checked for effect of BMI, cholesterol, BP, leukocyte count, education)	Total carotenoids, cases = 1.59 µM, controls = 1.81 µM. B-carotene, cases = 0.168, controls = 0.216 µM	For total carotenoids, low vs high quintile OR=1.84 (p=0.033). For a 0.75 µM increase in total carotene (~1 SD), the estimated RR equaled 0.65 (0.44-0.97).	Results much stronger for lung cancer than for other sites.
Epplein 2009 lung	Multiethnic Cohort Study	Nested case-control					207 (414)	age, sex, ethnicity, study loc (Hawaii or Calif.), smoking, date/time of collection, hrs of fasting, education, family history of lung cancer	Info on mean, 25, 75th percentile for men, women, both cases and controls for each carotenoid. Total here: Men case 2.206, (1.642-2.996), controls 2.756 (2.011-3.400). Women case 2.417 (1.583-3.628), controls 2.398 (1.756-3.160)<br>Median across tertiles, µM<br>a-carotene: 0.0447, 0.0838, 0.1863<br>b-carotene: 0.162, 0.376, 0.926<br>b-cryptoxanthin: 0.148, 0.298, 0.6385<br>lutein/zeaxanthin: 0.4395, 0.6908, 1.0952<br>lycopene: 0.3055, 0.5513, 0.8624<br>Total carotenoids: 1.666, 2.527, 3.725	A-carotene OR=0.24 (0.11-0.53)<br>B-carotne OR=0.30 (0.15-0.64)<br>b-cryptoxathin OR=0.33 (0.15-0.73)<br>lutein/zeaxanthin OR=0.45 (0.21-0.94)<br>lycopene OR=0.36 (0.18-0.75)<br>Total carotenoid OR=0.32 (0.15-0.68)	You can see immediately why there is no effect in women. The cases have higher carotenoid levels.<br>Some of the higher levels seen in these studies, yet still seeing effects at the higher level, not just a very low threshold.
Eichholzer 1996	Basel, Switzerland	Prospective cohort	0		2974	17	290 cancer (87 lung, 30 prostate, 28 stomach, 22 colon)	cholesterol, smoking, age,	Mean values, adj for cholesterol<br>Survivors 0.43±0.01 µM, lung 0.31 ±0.02, stomach 0.30±0.03, all cancer 0.35±0.01, other causes 0.35±0.01 µM.	Low retinol and low carotene, excluding first 2 years follow-up (all analyses), RR=3.56 (1.43-8.86). Risk of stomach cancer elevated for low carotene when carotenes measured as contiuous variable (RR=3.30 (1.42-7.70). All cancers for low retinol and low carotene RR=2.15 (1.47-3.16).	For vit E < 30.02 µM and vit C < 22.7 µM, excluding first 2 years of follow-up, RR=3.76 (1.63-8.71) for lung cancer. Low vit E and being a smoker had dramatic elevated risk for prostate cancer, though case numbers were small RR=19.87 (3.60-109.80).
Goodman 2003	see entry under prostate cancer										
Holick 2002	Alpha-Tocopherol, Beta-Carotene Cancer Prevention Study, Finland	Prospective cohort	0	50-69	27,084	14	1644 lung	Age, years smoked, cigarettes/day, intervention, supplement use (b-carotene and vit A), serum cholesterol	Quintiles of b-carotene: Q1 <0.184, Q2 0.184-0.274, Q3 0.276-0.374, Q4 0.376-0.540, Q5 >0.540 µM. Median quintile values: 0.134, 0.227. 0.319. 0.443, 0.717 µM.	Lower risks of lung cancer were observed for the highest versus the lowest quintiles of lycopene (28%), lutein/zeaxanthin (17%), β-cryptoxanthin (15%), total carotenoids (16%), serum β-carotene (19%), and serum retinol (27%).<br>Serum b-carotene RR=0.81 (0.69-0.95, p-trend 0.02) for multivariate risk. Among those w/5-19 cig/d, RR=0.65 (0.49-0.88, p-trend 0.02)	Dietary intake of fruits and vegetables also associated with lower lung cancer risk.
Ito 2003	Japan Collaborative Cohort Study	Nested case-control			39140	8	147 lung (311), 113 M, 34 W	Age, sex, participating institution, smoking, alcohol, BMI, serum total cholesterol	Individual carotenoids given in paper. Total carotenoids, cases 1.74 (0.55-4.71), controls 1.87 (0.48-5.20) µM. Geometric mean (5-95th%).	The risk of lung cancer death for the highest quartile of serum α-carotene, β-carotene, lycopene, β-cryptoxanthin, and canthaxanthin was significantly or marginally significantly lower than for the lowest quartile: the ORs, adjusted for smoking and other covariates, were 0.35 (95% confidence interval (Cl), 0.14–0.88), 0.21 (0.08–0.58), 0.46 (0.21–1.04), 0.44 (0.17–1.16) and 0.37 (0.15–0.91), respectively. Total carotenoids OR=0.27 (0.10-0.70).	The carotenes were stronger protection than the xanthophylls in this case.

Study, N=111	Study, Country	Study Type	% female	Age range	Cohort Size	Follow-up, years	Cases (and controls, if applicable)	Adjustments	Range of Carotenoids	Effect Size, (95% CI) from lowest to highest	Comments
Ito 2005b	Japan Collaborative Cohort Study	Nested case-control			39242	10	211 (163 men, 48 women), (487 controls, 375 men, 112 women)	Matched for age, sex, participating institution, smoking, alcohol, BMI, total cholesterol	Men, cases geometric mean (5-95% range) total carotenoids 1.50 (0.54-3.62) controls 1.65 (0.49-4.22) µM Women, cases 2.33 (0.74-5.45), controls 2.56 (0.82-6.45) µM Cut-offs for quartiles: a-carotene: 0.032, 0.053, 0.089; b-carotene <0.14, 0.29, >0.58; lycopene: <0.04, 0.06, >0.15. For women a-carotene: <0.058, 0.09, >0.150; b-carotene <0.40, 0.74, >1.20; lycopene: <0.07, 0.11, >0.20	Men, a-carotene (4 vs 1 quartile) OR=0.40 (0.18-0.86) p-trend=0.02; b-carotene OR=0.23 (0.09-0.55) p-trend<0.01; lycopene OR=0.44 (0.19-1.05) p-trend=0.03; b-cryptoxanthin OR=0.32 (0.13-0.78) p-trend=0.03; total carotenoids OR=0.42 (0.19-0.95) p-trend=0.09, not signif.	Same cohort as the 2003 report, but stronger numbers. Same conclusions. Number of cases of lung cancer only dropped in the highest quartile of carotenoids for men, indicating that these levels and not lower were protective. No signif trends in women, numbers of lung cancer cases is small compared to the men.
Menkes 1986	Maryland, USA						99 (196)	Age, sex, race, month of blood donation, smoking matched for cases and controls	see Comstock 1997	Risk of squamous cell lung cancer OR=4.30 (1.38-13.41) for serum b-carotene.	See Comstock 1997 for more on this same cohort.
Nomura 1985	Honolulu Heart Program, Hawaii	Nested case-control	0		6800	10	81 colon, 74 lung, 70 stomach, 32 rectum, 27 urinary bladder, (302 controls)	age, smoking	Quintiles of b-carotene: Q1 0-0.279, Q2 0.281-0.466, Q3 0.468-0.643, Q4 0.644-1.062, Q5 1.063-5.802 µM	Unadjusted OR, lowest vs highest Quintile, b-carotene = 3.4 (1.4-8.4). Adjusteid for age and smoking OR=2.2 (0.8-6.0) (not signif.) B-carotene seems to have a protective effect even for smokers, see 2nd graph,	
Ratnasinghe 2000	Chinese tin miners, high radon, alcohol	case control	0	63±6.5		0	108 (216)		tertiles of b-carotene: T1 <0.168, T2 0.186-0.335, T3 >0.354 µM. Tertiles of lut/zea: T1 <0.773, T2 0.791-1.055, T3 >1.072 µM. Tertiles of b-cryptoxanthin: T1 <0.072, T2 0.090-0.127, T3 >0.145 µM	showed increased risk of lung cancer with increased b-carotene levels and increased b-cryptoxanthin levels. Alcohol consumption greatly increased the above trend for increased lung cancer with higher carotenoids.	cases and controls have almost the exact same average levels of individual carotenoids, except that b-carotene levels are slightly higher.
Yuan 2001	Shanghai, China	Prospective cohort	0	45-64	18,244	12	209 lung (627)	Matched for age, month and year of blood sample collection, neighborhood of residence. Adjusted for smoking	Have all in separate tab sheet here for individual carotenoids. For total carotenoids, Q1=<0.743, Q2 0.743-0.941, Q3 0.9415-1.221, Q4 >1.221 µM	Compared to lowest quartile of b-cryptoxanthin 2nd, 3rd, and 4th quartile categories were 0.72 (0.41–1.26), 0.42 (0.21–0.84), and 0.45 (0.22–0.92), respectively (P for trend = 0.02). A statistically significant 37% reduction in risk of lung cancer was noted in smokers with above versus below median level of total carotenoids.	
**Breast Cancer, N=12**											
Ching	Australia	case control	100	30-84			153 (151)	Age at menarche, parity, alcohol, total fat intake	Quartiles of b-carotene: Q1 ≤0.4, Q2 0.4-0.7, Q3 0.7-1.1, Q4 ≥1.1 µM	b-carotene, high vs lowest quartile OR=0.47 (0.24-0.91), p,trend=0.016	
Dorjgochoo 2009	Shanghai Womens Health Study	nested case control	100	40-70	74,942	7.5	365 (726)	Matched for age, date and time of blood collection, interval fasting before blood sample, menopausal status, antibiotic use in past week. Linear regressions adjusted for BMI, education, occupation, age at menarche, age at 1st live birth, waist to hip ratio, exercise, smoke, history of brest fibroadenoma, 1st degree family history cancer, total intakes of energy, vegetables, fruit, red meat and fish, tea consumption, batch for assays	Total carotenoid range, median (25th, 75th percentile) Cases: 2.43 (1.89-2.99), Controls: 2.21 (1.86-2.86) µM	We observed no associations between breast cancer risk and any of the tocopherols, retinol, and most carotenoids. However, high levels of plasma lycopene other than trans, 5-cis and 7-cis or trans α-cryptoxanthin were inversely associated with the risk of developing breast cancer. Total carotenoids have increased risk in post-menopausal women, OR=1.77 (0.96-3.25) p,trend=0.05, for 4th vs 1st quartile.	

Study, N=111	Study, Country	Study Type	% female	Age range	Cohort Size	Follow-up, years	Cases (and controls, if applicable)	Adjustments	Range of Carotenoids	Effect Size, (95% CI) from lowest to highest	Comments
Epplein 2009	Multiethnic Cohort Study (Hawaii, Calif.)	nested case control	100				286 (535)	match for age, sex, ethnicity, study location, smoking status, date/time of collection and hours fasting.	Total carotenoid quartiles, µM. Q1 ≤2.072, Q2 2.072-2.771, Q3 2.771-3.583, Q4 ≥3.583 µM	Women with breast cancer tended to have lower levels of plasma carotenoids and tocopherols than matched controls, but the differences were not large or statistically significant and the trends were not monotonic	
Ito 1999	Madras, India	case control	100	22-80			206 (150 hospital controls w/other cancer) (61 healthy)	healthy controls did not smoke or drink. Few of the others did either.	Total carotenoids, mean±SD. Breast cancer 0.847±0.334, Hosp. controls 0.725±0.387, healthy controls 1.181±0.526 µM. Individual carotenoids also in paper and sorted by pre- and post-menopausal status.	just gives signif. Diff. of means, T-test type of stats. Compared to healthy controls, breast cancer patients had stastically lower conc. Of a- and b-carotene, lycopene, cryptoxanthin, zeaxanthin/lutein, and total carotenoids. Differences were greater in the post-menopausal group.	
Kabat 2009	Women's Health Initiative, USA	nested case control	100	62±7	5450	8	190	age, education, ethnicity, BMI, oral contraceptive use, hormone therapy, age at menarthe, age at first birth, age at menopause, alcohol, family history of breast cancer, breast biopsy, physical activity, energy intake, randomization status. Smoking and serum cholesterol did not have an effect on analysis.	Cut-offs for Tertiles. A-carotene: 0.0745, 0.1676; b-carotene: 0.317, 0.615; b-cryptoxanthin: 0.109, 0.181; lutein/zeaxanthin: 0.281, 0.422; lycopene: 0.559, 0.875	After multivariable adjustment, risk of invasive breast cancer was inversely associated with baseline serum **α-carotene** concentrations (hazard ratio for highest compared with the lowest tertile: 0.55; 95% CI: 0.34, 0.90; P = 0.02) and positively associated with baseline **lycopene** (hazard ratio: 1.47; 95% CI: 0.98, 2.22; P = 0.06). Analysis of repeated measurements indicated that α-carotene and β-carotene were inversely associated with breast cancer	Lycopene is probably opposite because it comes from different foods that are accessible on western diet
Kato 2006	Nutrtiion and Breast Health Study, USA	cross section	100	post-meno pause	71	1				The probability of detecting epithelial cells in NAF decreased with increasing plasma levels of lutein and alpha-carotene (p-values for linear trend; 0.001 and 0.049, respectively). The ORs for the highest versus lowest quartile levels are 0.17 (95% CI 0.04-0.65) and 0.19 (95% CI 0.04-0.91), respectively	
Maillard 2010	EPIC, France	prospectiv e cohort	100	56.8±6 .3	19934	7	366, 84 pre- and 282 post-menopausa l (720 controls)	Controls matched to age, menopausal status, fasting status at blood collection, date and collection center, multivariate adjustment for alcohol, menopausal hormone use, education, age at first birth and parity, family history of breast cancer in first degree relative, personal history of benign breast disease.	Individual and total carotenoid mean±SD in text for controls and cases. Total carotenoid, controls = 2.19±0.80, cases = 2.16±0.78 µM (identical)	no significant effects over quintiles of carotenoids	null study
Rock 2005	WHEL, Women w/history of early stage breast cancer	control arm of WHEL study, so prosp. Cohort	100		1551	7	205	tumor stage, grade, hormone receptor status, chemotherapy and tamoxifen therapy, clinical site, age at diagnosis, BMI, plasma cholesterol	Quartiles of total carotenoids: Q1 0.268-1.392, Q2 1.393-1.989, Q3 1.989-2.863, Q4 2.867-22.920 µM. Mean values for quartiles are 1.038, 1.683, 2.375, and 4.189 µM	highest vs lowest quartile of total serum carotenoids, HR=0.57 (0.37-0.89).	
Rock 2009	Women's Healthy Living and Eating Study (WHEL)	RCT, but looked at plasma values of all women	100	53	3043	7.12	508		Tertiles of total carotenoids. T1 ≤1.656, T2 1.675-2.452, T3 >2.452 µM	Compared with the lowest tertile, the hazard ratio for the medium/high plasma carotenoid tertiles was 0.67 (95% confidence interval, 0.54-0.83) after adjustment	

Study, N=111	Study, Country	Study Type	% female	Age range	Cohort Size	Follow-up, years	Cases (and controls, if applicable)	Adjustments	Range of Carotenoids	Effect Size, (95% CI) from lowest to highest	Comments
Sato 2002	Maryland, USA, samples in 1974 and 1989	nested case control	100			≥10	295 (295)	matched on age, race, menopausal status, date of blood donation	Individual ranges for carotenoids given in paper. Total carotenoids quintiles are Q1 <.943, Q2 0.945-1.316, Q3 1.317-1.683, Q4 1.683-2.229, Q5 ≥2.231 µM	The risk of developing breast cancer in the highest fifth was approximately half of that of women in the lowest fifth for β-carotene [odds ratio (OR) = 0.41; 95% confidence interval (CI) 0.22–0.79; P trend = 0.007], lycopene (OR = 0.55; 95% CI 0.29–1.06; P trend = 0.04), and total carotene (OR = 0.55; 95% CI 0.29–1.03; P trend = 0.02) in the 1974 cohort.	signif effects seen in the 1974 cohort, but not in the 1989 cohort. Total carotenoids not that much different.
Tamimi 2005	Nurses' Health Study	nested case control	100	30-55	32,86		969	controls matched to age, benopausal status, post-menopausal hormone use, time of day, month, and fasting status at blood draw. Regression adjusted for BMI at age 18, weight gain since 18, age at menarche, parity/age at first birth, family history of breast cancer, history of benign breast disease, alcohol, post-menopausal hormone use	Quintiles of total carotenoids, median values. 1.01, 1.48, 1.85, 2.27, 3.05 µM. Individual carotenoid values in text.	The multivariate risk of breast cancer was 25-35% less for women with the highest quintile compared with that for women with the lowest quintile of **a-carotene** (odds ratio (OR) = 0.64, 95% confidence interval (CI): 0.47, 0.88; ptrend = 0.01), **b-carotene** (OR = 0.73, 95% CI: 0.53, 1.02; ptrend = 0.01), **lutein/zeaxanthin** (OR = 0.74, 95% CI: 0.55, 1.01; ptrend = 0.04), and **total carotenoids** (OR = 0.76, 95% CI: 0.55, 1.05; ptrend = 0.05). The inverse association observed with {alpha}-carotene and breast cancer was greater for invasive cancers with nodal metastasis.	Drop in risk mostly seen in the upper quintile, not in the lower ones in this study. Important to note.
Toniolo 2001	New York University Women's Health Study	nested case control	100	35-65	14275		270 (270) 125 pre- and 145 post-menopausal	Age at menarche, age at 1st full term pregnancy, parity, history of breast cancer in 1st degree relatives, history of benign breast conditions, BMI, serum cholesterol. Only age at first full term pregnancy, family history, and benign breast conditions, and cholesterol in final model.	Only mean±SD given. Individual carotenoids in text. Total carotenoid mean±SD, cases = 2.306±0.868, controls = 2.593±1.066 µM	The risk of breast cancer approximately doubled among subjects with blood levels of β-carotene at the lowest quartile, as compared with those at the highest quartile (odds ratio = 2.21; 95% confidence interval (CI): 1.29, 3.79). The risk associated with the other carotenoids was similar, varying between 2.08 (95% CI: 1.11, 3.90) for lutein and 1.68 (95% CI: 0.99, 2.86) for β-cryptoxanthin. The odds ratio for the lower quartile of total carotenoids was 2.31 (95% CI: 1.35, 3.96).	Seems like the drop in risk here, too, is in the top quintile. It appears different from the other quintiles, which seem statistically to be more similar. The OR for the quintiles are about the same except for the upper quintile.
**Prostate Cancer, N=13**											
Chang 2005	Texas, USA	case control	0	~62±7		0	118 (52)	Age, smoking, height, family history	Total carotenoids, controls 1.14 (0.88-1.63), cases 1.18 (0.82-1.45) µM. (25-75%). Differences are small between cases and controls.	Risk for men with high plasma levels of alpha-carotene, trans-beta-carotene, beta-cryptoxanthin, and lutein and zeaxanthin was less than half that for those with lower levels. A-carotene 0.33 (0.16-0.72), b-carotene 0.46 (0.21-0.98), lut/zea 0.32 (0.15-0.69), b-crypt 0.48 (0.23-1.02).	low levels of carotenoids overall. Small study, very few controls.

Study, N=111	Study, Country	Study Type	% female	Age range	Cohort Size	Follow-up, years	Cases (and controls, if applicable)	Adjustments	Range of Carotenoids	Effect Size, (95% CI) from lowest to highest	Comments
Gann 1999	Physicians' Health Study, USA	nested case control	0	40-84	22,071	13	578 (1294)	Age and smoking matched controls, BMI, exercise frequency, alcohol, total cholesterol, multivitamin use	Quintiles for carotenoids:<br>a-carotene: 0.064, 0.094, 0.126, 0.192 µM<br>b-cryptoxanthin: 0.067, 0.097, 0.136, 0.202 µM<br>lutein: 0.107, 0.139, 0.179, 0.258 µM<br>lycopene: 0.487, 0.659, 0.825, 1.081 µM	The ORs for all prostate cancers declined slightly with increasing quintile of plasma lycopene (5th quintile OR 0.75 (0.54 –1.06; P, trend 0.12); there was a stronger inverse association for aggressive prostate cancers (5th quintile OR 0.56 (0.34–0.91; P, trend 0.05). In the placebo group, plasma lycopene was very strongly related to lower prostate cancer risk (5th quintile OR=0.40; P, trend 0.006 for aggressive cancer), whereas there was no evidence for a trend among those assigned to b-carotene supplements. However, in the b-carotene group, prostate cancer risk was reduced in each lycopene quintile relative to men with low lycopene and placebo.	levels of carotenoids overall pretty low.
Gill 2009 (null)	Multiethnic Cohort, Hawaii, Calif.	nested case control	0	45-75	96382 29,009 w/plasma		467 (934)	Matched for ethnicity, age, date and time of blood collection, fasting status. BMI, family history, education	Ranges, µM. A-carotene: 0.183, 0.352, 0.555, 1.112<br>lycopene: 0.41, 0.631, 0.861, 1.222; b-cryptoxnathin: 0.250, 0.400, 0.557, 1.017;<br>lutein/zeaxanthin: 0.473, 0.647, 0.814, 1.099;<br>Total carotenoid: 1.842, 2.5522 3.310, 4.712 µM	b-carotene OR=0.81 (0.55-1.18) 1st vs 4th Quartile. Lycopene OR=0.78 (0.53-1.14, p,trend=0.16). B-crypt OR=0.97 (0.66-1.43); Lut/zea OR=1.08 (0.73-1.61); Total carotenoids 1.00 (0.67-1.49)	seems like the range of carotenoids included some higher levels here.
Goodman 2003	b-Carotene and Retino/ Efficacy Trial, USA, CARET	nested case control	0	45-69	4060 asbestos, 7982 heavy smokers		278 (lung cancer) 205 (prostate) (483 controls)	Matched for high-risk population, study center, location, age, sex (for lung cancer), smoking, year of randomization	Quartile cut-off points, µM. A-carotene: 0.034, 0.056, 0.080; b-carotene: 0.175, 0.259, 0.408; b-cryptoxanthin: 0.065, 0.099, 0.163; lutein: 0.181, 0.237, 0.304; zeaxanthin: 0.037, 0.047, 0.063; lycopene: 0.427, 0.598, 0.777 µM. Polar carotenoids: 0.328, 0.419, 0.540; Non-polar carotenoids: 0.667, 0.916, 1.223 µM; Total carotenoids	Statistically significant trends for lung cancer across quartiles were observed in **lutein** (P = 0.02), **zeaxanthin** (P = 0.02), and α-tocopherol (P = 0.03). Statistically significant odds ratios (ORs) comparing 4th to 1st quartiles in the female population were seen in **lutein** [OR, 0.31; confidence interval (CI), 0.13–0.75], **zeaxanthin** (OR, 0.31; CI, 0.12–0.77), and **β-cryptoxanthin** (OR, 0.34; CI, 0.14–0.81). For prostate cancer, mean serum concentrations were lower in cases for all of the nutrients except α-carotene. Only for α-tocopherol (Ptrend = 0.04) were the findings statistically significant. There was no statistically significant association between serum carotenoids and prostate cancer.	The carotenoid findings in the overall population were because of the strong inverse association between serum micronutrients and lung cancer in females.
Huang 2003	Maryland, USA, CLUE I and CLUE II (1974, 1989)	nested case control	0		20260		182 (1974) 142 (1989) (2 controls /case)		Medians and interquartile ranges for controls 1974<br>a-carotene 0.043 (0.026-0.065) µM<br>b-carotene 0.153 (0.093-0.259)µM<br>b-cryptoxanthin 0.104 (0.054-0.209) µM<br>lutein 0.250 (0.183-0.332) µM<br>lycopene 0.665 (0.456-0.959) µM. Gives quintile cutoffs for individual carotenoids.	No effect for carotenoids. Cases and controls have about same levels. Of course there is no effect, because levels of all of them are way low. Maybe not a large enough range to see a difference.	lycopene levels are higher than b-carotene levels, not indicative of high vegetable intake.
Hsing 1990 (null)	Marylane, USA CLUE I (1974)	nested case control	0			13	103 (103)	age, race		no significant associations with any carotenoids	
Key 2007	EPIC, Europe	nested case control	0		137001	6	966 (1064)	matched for study center, age, date of recruitment, adjusted for smoking, alcohol, BMI, marital status, physical activity, education level	lycopene quintile cutoffs: 0.28, 0.45, 0.65, 0.92 µM; total carotenoid quintiles cutoffs: 1.09, 1.45, 1.82, 2.32 µM	for advanced disease, lycopene quintile 5 vs 1 OR=0.40 (0.19-0.88) and for total carotenoids OR=0.35 (0.17-0.78)	no association with localized disease; only the upper quintile was signifcantly different from the lowest quintile.

Study, N=111	Study, Country	Study Type	% female	Age range	Cohort Size	Follow-up, years	Cases (and controls, if applicable)	Adjustments	Range of Carotenoids	Effect Size, (95% CI) from lowest to highest	Comments
Lu 2001	USA	case control	0				65 (132)	age, race, years of education, daily caloric intake, pack-years of smoking, alcohol consumption, and family history of prostate cancer	Quartile cutoff points, µM a-carotene 0.03455, 0.05865, 0.10686; b-carotene 0.1242, 0.174, 0.3411; b-cryptoxanthin: 0.04496, 0.0703, 0.1326; lutein: 0.0840, 0.1325, 0.1947; zeaxanthin: 0.0310, 0.05285, 0.08309; lycopene: 0.1787, 0.27515, 0.40098 µM	**Lycopene** [odds ratio (OR), 0.17 (0.04–0.78; P for trend, 0.0052] and **zeaxanthin** (OR, 0.22 (0.06–0.83; P for trend, 0.0028) when comparing highest with lowest quartiles. Borderline associations were found for **lutein** (OR, 0.30 (0.09–1.03; P for trend, 0.0064) and **β-cryptoxanthin** (OR, 0.31; (0.08–1.24; P for trend, 0.0666). No obvious associations were found for α- and β-carotenes.	
Nomura 1997 (null)	Honolulu heart program, Japanese American ethnic	nested case control	0	52-74 (avg 62) at time of diagnosis	6860	>20	142 (142)	matched for hour of examination, age at exam, month and year of exam	Median levels for controls (not signif diff from patients) Total carotenoids 1.63 µM, lutein, 0.207, zeaxanthin 0.042, b-cryptoxanthin 0.2207, lycopene 0.2496, a-carotene 0.0763, b-carotene 0.264 µM	no significant associations with any carotenoids	
Peters 2007	Prostate, Lung, Colorectal, and Ovarian Cancer Screening Trial	nested case control					692 (270 aggressive) 844 controls	matched for age, time since initial screening, year of blood draw. Checked for other factors that were, in the end, not significant-- family history of prostate cancer, education, physical activity, BMI, aspirin and ibuprofen use, history of diabetes, smoking, alcohol, energy, fat, red meat, heterocyclic amines from meat, fruits, vegetables, cruciferous vegetables, vit E, calcium, and serum selenium.	Median level for quintiles, µM<br>a-carotene: 0.048, 0.088, 0.129, 0.192, 0.309<br>b-carotene: 0.114, 0.192, 0.283, 0.410, 0.721<br>b-cryptox: 0.02, 0.090, 0.123, 0.172, 0.304<br>lutein: 0.163, 0.234, 0.302, 0.394, 0.554<br>zeaxathinh: 0.056, 0.084, 0.104, 0.134, 0.181<br>lycopene: 0.568, 0.872, 1.159, 1.462, 2.019	No association was observed between serum **lycopene** and total prostate cancer [odds ratios (OR), 1.14; 95% confidence intervals (95% CI), 0.82-1.58 for highest versus lowest quintile; P for trend, 0.28] or aggressive prostate cancer (OR, 0.99; 95% CI, 0.62-1.57 for highest versus lowest quintile; P for trend, 0.433). **B-Carotene** was associated with an increased risk of aggressive prostate cancer (OR, 1.67; 95% CI, 1.03-2.72 for highest versus lowest quintile; P for trend, 0.13); in particular, regional or distant stage disease (OR, 3.16; 95% CI, 1.37-7.31 for highest versus lowest quintile; P for trend, 0.02); other carotenoids were not associated with risk.	high levels of lycopene, but overall low total carotenoids.
Vogt 2002	Population based, multi-center, USA	case control	0	40-79			209 (228)	study center, race, age, month of blood draw only significant other factor, though many tested see text for details).	Ranges for quartiles, µM<br>a-carotene: 0-0.026, 0.028-0.060, 0.061-0.088, 0.089-0.548<br>b-carotene: 0.006-0.153, 0.155-0.276, 0.278-0.430, 0.600-2.189<br>b-cryp: 0.005-0.089, 0.090-0.128, 0.130-0.201, 0.203-0.801<br>lut/zea: 0.063-0.248, 0.250-0.345, 0.346-0.468, 0.469-1.420<br>lycopene: 0.009-0.199, 0.201-0.319, 0.320-0.460, 0.464-1.069	**Lycopene** was inversely associated with prostate cancer risk (comparing highest with lowest quartiles, odds ratio (OR) = 0.65(0.36, 1.15; test for trend, p = 0.09), particularly for aggressive disease (comparing extreme quartiles, OR = 0.37, (0.15, 0.94; test for trend, p = 0.04). Other carotenoids were positively associated with risk. For all carotenoids, patterns were similar for Blacks and Whites. Trend for lycopene not significant for localized cancer, only for aggressive cancers.	

Study, N=111	Study, Country	Study Type	% female	Age range	Cohort Size	Follow-up, years	Cases (and controls, if applicable)	Adjustments	Range of Carotenoids	Effect Size, (95% CI) from lowest to highest	Comments
Wu 2004	Health Professionals Follow-up Study	nested case control	0				450 (450)	matched for age, time, month, and year of blood donation. Multivariate model adjusted for family history of prostate cancer, history of vasectomy, BMI, height, vigorous exercise, smoking, vit E suppl, selenium suppl, plasma cholesterol.	Medians of quintiles not given. Median levels for controls (not signif diff from cases). Total carotene (carotenoid?) 1.625 µM, lutein/zeaxanthin 0.218, b-cryptoxanthin 0.116, a-carotene 0.107, b-carotene 0.351, lycopene 0.726 µM	**α-carotene**: OR, 0.67 [95% confidence interval (CI), −0.40–1.09]; **β-carotene**: OR, 0.78 (95% CI, 0.48–1.25); **lycopene**: OR, 0.66 (95% CI, 0.38–1.13)}. The inverse association between plasma lycopene concentrations and prostate cancer risk was limited to participants who were 65 years or older (OR, 0.47; 95% CI, 0.23–0.98) and without a family history of prostate cancer (OR, 0.48; 95% CI, 0.26–0.89). Combining, older age and a negative family history provided similar results (OR, 0.43; 95% CI, 0.18–1.02). Inverse associations between β-carotene and prostate cancer risk were also found among younger participants (<65 years of age; OR, 0.36; 95% CI, 0.14–0.91; = 0.03).	
Zhang 2007	Arkansas, USA	population based case control	0				193 (197)	matched by age, race, county of residence. Model adjusted for BMI, education, smoking. 1 model adjusted for other four carotenoids (not the one I quote).	Median levels for quartiles, µM<br>a-carotene: 0.013, 0.027, 0.045, 0.113<br>b-carotene: 0.065, 0.135, 0.362, 0.528<br>b-crypt: 0.058, 0.106, 0.156, 0.248<br>lut/zea: 0.149, 0.236, 0.304, 0.417<br>lycopene: 0.262, 0.429, 0.621, 0.957	Highest vs lowest quartile of lycopene: OR=0.45 (0.24-0.85) p,trend = 0.042	
**Colon Cancer, N=3 (1 more is a repeat)**											
Eichholzer 1996	see entry under lung cancer									No signif. Results for colon cancer. Only 22 cases.	
Jiang 2005	Nagoya, Japan	case control	29.7	50-74		0	Men 159 (160) Women 65 (70)	Age, sex, history of colorectal ademomas and cancers, BMI, smoking, alcohol, multivitamin use, plasma cholesterol, vigorous exercise	Total Carotenoids, quartiles<br>Men: <1.36, 1.36–1.91, 1.92–2.50, >2.50;<br>Women: <1.91, 1.91–2.63, 2.64–3.06 >3.06<br>Geom mean (5-95%) Men, cases: 1.61 (0.66–3.95), controls: 1.87 (0.79–4.45). Women, cases: 2.29 (1.04-4.89), controls: 2.41 (1.13-5.18).	In men, a-carotene highest quartile vs lowest OR=0.38 (0.18-0.84, p-trend=0.01); b-carotene OR=0.48 (0.22-1.03, p-trend=0.04); total carotenoids OR=0.48 (0.22-1.03, p-trend=0.04). In women a-carotene OR=0.53 90.19-1.52, p-trend=0.35)	
Steck-Scott 2004	Polyp Prevention Trial, USA	prospective cohort	33	≥35, Avg=61	781	4	323 reoccurrence, 147 multiple, 217 RHS, 164 LHS	age, NSAIDS use, sex, intervention group, gender*group interaction	Median value in quartile, µM<br>a-carotene: 0.056, 0.075, 0.180, 0.224<br>b-carotene: 0.168, 0.279, 0.447, 0.820<br>b-cryptoxanthin: 0.090, 0.145, 0.217, 0.307<br>lut/zea: 0.246, 0.352, 0.457, 0.668<br>lycopene: 0.224, 0.354, 0.484, 0.671<br>Total carotenoids: 0.972, 1.376, 1.780, 2.495	Compared to the lowest quartile of baseline alpha-carotene concentrations, the OR of multiple polyp recurrence for the highest quartile was 0.55 (95% CI = 0.30–0.99) and the OR of right-sided recurrence was 0.60 (95% CI = 0.37–0.95).<br>Compared to the lowest quartile of averaged beta-carotene concentrations, the OR of multiple adenomas for the highest quartile was 0.40 (95% CI = 0.22–0.75) with an inverse trend (p = 0.02). For total carotenoids, for recurrence of multiple polyps OR=.52 (0.29-0.95, P-trend=0.06)	Only the upper quartile is significantly different from the lowest quartile in the multiple regression model.

Study, N=111	Study, Country	Study Type	% female	Age range	Cohort Size	Follow-up, years	Cases (and controls, if applicable)	Adjustments	Range of Carotenoids	Effect Size, (95% CI) from lowest to highest	Comments
Wakai 2005	Japan Collaborative Cohort Study	nested case control	52.9	40-79	116	8	116 (298) 54 men (141 men controls; 62 women (157 women controls)	sex, age, education, family history colorectal cancer, BMI, smoking, alcohol, walking (time/day), sedentary work, consumption of beef, total cholesterol	Geometric mean, (5-95% range) Men total carotenoids cases 1.59 (0.52-4.87) controls 1.79 (0.64-4.68) µM; Women cases 2.55 (0.80-5.88) controls 2.33 (0.75-5.00) µM. Individual carotenoids also in text. Have tertile cut-offs for indiv and total in text as well. Men total carotenoids: 1.48, 2.21; women 2.06, 2.96 µM	In **men,** the higher level of serum total carotenoids was associated with a decreased risk: The multivariate-adjusted odds ratio (OR) for the highest vs. the lowest tertile was 0.34 (95% confidence interval [CI] = 0.11-1.00; trend P over tertiles = 0.040)<br>In **women,** the higher levels of alpha- and and total carotenoids were instead related to an increased risk: The corresponding ORs were 4.72 (95% CI = 1.29-17.3), 2.00 (0.70-5.73), and 2.47 (0.73-8.34), respectively (trend P = 0.007, 0.040, and 0.064, respectively)	Opposite results in men and women. Overall the women have higher carotenoid levels, so other factors also become more important.
**Stomach Cancer, N=4**											
Ito & Suzuki 2001	Hokkaido, Japan	Cross-section	63.1		206	0	91	age, sex, more adjustments did not dampen the ORs.	No levels given in full paper.	the odds ratios for serum carotenoid levels were lower for subjects with high serum levels of α-carotene (odds ratio, 0.41; 95% C.I., 0.19-0.88) and β-carotene (odds ratio, 0.41; 95% C.I., 0.18-0.91) than for those with low serum carotenoid levels. In addition, the odds ratios of subjects with high serum levels of β-cryptoxanthin (odds ratio, 0.60; 95% C.I., 0.28-1.31), provitamin A (odds ratio, 0.38; 95% C.I., 0.17-0.85), and retinol (odds ratio, 0.67; 95% C.I., 0.31-1.48) were found to be lower than the odds ratios for those with low serum levels. Odds ratios for subjects with high serum zeaxanthin/lutein levels were higher than odds ratios for those with low serum levels, but not stat. signif.	result for lutein/zeaxanthin is odd.
Jenab 2006	EPIC, Europe, 10 countries	nested case control	45.3	mean age of cases/ controls = 59.1		3.2	244 (645)	matched by age, sex, study centre, date of blood donation. Multilinear adjustment for BMI, total energy intake, smoking, Helicobacter pylori infection status	Range of carotenoids (5-95th%), cases only<br>a-carotene: 0.028-0.415 µM<br>b-carotene: 0.104-0.998 µM<br>b-cryptoxanthin: 0.036-0.693 µM<br>lutein: 0.156-0.809 µM<br>zeaxanthin: 0.023-0.160 µM<br>lycopene: 0.145-1.317 µM<br>Total carotenoids: 0.741-3.824 µM<br><br>Quartile cut-offs, µM<br>a-carotene: 0.060, 0.101, 0.168<br>b-carotene: 0.224, 0.332, 0.494<br>b-cryptoxanthin: 0.105, 0.194, 0.338<br>lutein: 0.257, 0.352, 0.508<br>zeaxanthin: 0.056, 0.083, 0.118<br>lycopene: 0.332, 0.544, 0.834<br>Total carotenoids: 1.294, 1.811, 2.494	highest vs lowest quartiles b-cryptoxanthin OR=0.30 (0.30-0.94, p-trend=0.006)<br>zeaxanthin (OR=0.39, (0.22-0.69. p-trend=0.005)<br>For cardial gastric cancer, total carotenoids (70 cases, 176 controls) OR(4 vs 1)=0.31 (0.08-1.16) p-trend=0.076. Not quite significant.	

Study, N=111	Study, Country	Study Type	% female	Age range	Cohort Size	Follow-up, years	Cases (and controls, if applicable)	Adjustments	Range of Carotenoids	Effect Size, (95% CI) from lowest to highest	Comments
Persson 2008	Japan Public Health Center-based Prospective Study	nested case control	33	40-69	######		511 (511)	Matched for age, sex. Adjusted for family history of gastric cancer, smoking, H. pylori infection, salt intake, consumption of highly salted foods (salted fish guts and roe), BMI	Quartile medians (max and min also in paper)<br>a-carotene: 0.017, 0.035, 0.052. 0.091, max 1.354<br>b-carotene: 0.084, 0.214, 0.380, 0.715, max 2.999<br>b-crypto: 0.056, 0.137, 0.284, 0.637, max 6.989<br>lut/zea: 0.387, 0.581, 0.779, 1.191 max 2.869<br>lycopene: 0.0, 0.091, 0.184, 0.358 max 1.395	b-carotene OR (2, 3, 4 vs 1) 0.63 (0.81-0.75), 0.48 (0.31-0.75) and OR=0.46 (0.28-0.75) p-trend <0.01. a-carotene p-trend = 0.04, b-carotene p-trend <0.01 for men only both.	inverse trends in men for b- and a-carotene, but not in women who had relatively higher levels of carotenoids.
Yuan 2004	Shanghai Cohort Study, Shanghai, China	nested case control	0		18,244	12	191 (570)	matched for age, year of blood collection, neighborhood of residence, adjusted for H. pylori, detectable urinary epigallocatechin (protective), smoking, alcohol	Geometric mean (95% CI of the mean) cases<br>a-carotene: 0.010 (0.009-0.011) µM<br>b-carotene: 0.147 (0.134-0.162)<br>b-crypt: 0.049 (0.043-0.052)<br>lut/zea: 0.526 (0.499-0.554)<br>lycopene: 0.041 (0.035-0.045)<br>Quartile cut-offs, µM<br>a-carotene ND, 0.015, 0.026<br>b-carotene 0.112, 0.175, 0.276<br>b-crypto: 0.033, 0.052, 0.078<br>lut/zea: 0.404, 0.529, 0.673<br>lycopene: 0.022, 0.047, 0.086	the odds ratios (95% confidence intervals) for the highest versus the lowest quartile of α-carotene, β-carotene, and lycopene were 0.38 (0.13-1.11), 0.54 (0.32-0.89), and 0.55 (0.30-1.00), respectively. (These are unadjusted Ors, only matched.) Also for non-smokers and <3 drinks/day b-carotene OR=0.31 (0.13-0.75) p-trend 0.008 for 4 vs 1st quartile.	Vitamin C levels in non-smoking, low alcohol men also associated with lower gastric cancer OR 0.39 (0.15-0.98) p-trend=0.02<br><br>Lutein/zeaxanthin levels are way higher than b-carotene levels. I think this means that yellow / orange vegetable intake was minimal in the whole population.
**Cervical cancer, dysplasia, N=4**											
Goodman 1998	Hawaii	case-control	100	18-65		0	147 (191)	Age, ethnicity, tobacco use, alcohol, HPV detection	Quartile cut-offs, µM<br>a-carotene: 0.061, 0.098, 0.161<br>b-carotene: 0.192, 0.320, 0.615<br>b-crypto: 0.114, 0.169, 0.270<br>a-crypto: 0.043, 0.059, 0.075<br>crypto total: 0.208, 0.286, 0.421<br>lut/zea: 0.571, 0.700, 0.882<br>lycopene: 0.430, 0.594, 0.756<br>Total carotenoids: 1.710, 2.183, 2.826 µM	OR, 4 vs 1st quartile: Total cryptoxanthin OR= 0.4 (0.2-1.0) p-trend=0.10; a-cryptoxanthin OR=0.3 (0.1-0.7) p-trend 0.01; Total carotenoids OR=0.6 (0.3-1.5) p-trend=0.10 (trend significant w/o adjust for smoke, alcohol, HPV)	
Nagata 1999	Japan	case-control	100				152 (152)	HPV, smoking, age-matched	Tertile medians, µM<br>a-carotene: 0.095, 0.158, 0.256<br>b-carotene: 0.639, 1.063, 2.061<br>cryptoxanthin: 0.228, 0.467, 1,256<br>lut/zea: 0.647, 0.937, 1.338<br>lycopene: 0.307, 0.573, 0.973	OR, 3 vs 1st tertile: **a-carotene** adjusted OR=0.16 (0.04-0.62), **b-carotene** OR=0.45 (0.25-0.82) but not adjsted for HPV or smoking; with adjust OR=0.65 (0.22-1.92) p-trend=0.38; **lycopene** OR=0.28 (0.08-1.01) p-trend=0.049; others not significant	
Palan 1996	Bronx Borough, New York City, USA	cross section	100	19-65	235		56 CIN I, 40 CIN II, 29 CIN III, 15 invasive cancer (95 control, 58 routine care at gynecologic al clinic, 37 who had biopsies with no CIN)	smoking checked	b-carotene: cancer 0.136±0.084, normal 0.371±0.311 µM; lycopene: cancer 0.628±0.224, normal 0.987±0.456 µM other grades of dysplasia inbetween.	There were significant linear trends for all three carotenoids and quadratic trends for alpha- and tau-tocopherol with the degree of cervical histopathology. Plasma beta-carotene concentrations in cigarette smokers were significantly lower regardless of cervical pathology, whereas plasma lycopene and canthaxanthin levels were significantly lower in smokers with CIN.	41% Blacks, 46% Hispanics

Study, N=111	Study, Country	Study Type	% female	Age range	Cohort Size	Follow-up, years	Cases (and controls, if applicable)	Adjustments	Range of Carotenoids	Effect Size, (95% CI) from lowest to highest	Comments
Schiff 2001	Southwestern American Indian women, USA	case-control	100	18-45			81 (1600	human papilloma virus test, income, urban vs rural residence, age	Tertiles, min, 1-2 cut, 2-3 cut, max µM a-carotene: 0.121, 0.237, 0.335, 1.626 b-carotene: 0.205, 0.447, 0.708, 3.241 b-cryptoxanthin: 0.040, 0.116, 0.186, 0.548 lut/zea: 0.211, 0.369, 0.492, 1.336 lycopene: 0.276, 0.572, 0.758, 1.300	Increasing levels of α-carotene, β-cryptoxanthin, and lutein/zeaxanthin were associated with decreasing risk of CIN II/III. In addition, the highest tertiles of β-cryptoxanthin (odds ratio = 0.39, 95% confidence interval = 0.17–0.91) and lutein/zeaxanthin (odds ratio = 0.40, 95% confidence interval = 0.17–0.95) were associated with the lowest risk of CIN	HPV was strong risk factor, OR=9.0 (4.7-17.5).
**Cancer, general, N=3 (3 more repeats)**											
Eichholzer 1996	Basel Study, Switzerland, see entry under lung cancer	prospective cohort	0			17	290 cancer (87 lung, 30 prostate, 28 stomach, 22 colon)				
Ito 1997	Comprehensive Health Examination Program (CHEP) rural Japan, Hokkaido	prospective cohort	63.7	39-83	2353	2-8 years	98 deaths (67 men, 31 women) 44 cancer (34 men, 10 women)	Age, smoking, alcohol	B-carotene,tertiles, men: <0.266, 0.266-0.592, >0.592; women: <0.682, 0.682-1.266, >1.266 A-carotene, terttiles, men: <0.065, 0.065-0.151, >0.151, women: <0.108, 0.108-0.229, >0.229 Lycopene, tertiles, men: <0.201, 0.201-0.449, >0.449, women: <0.300, 0.300-0.605, >0.605 Geometric means (5-95% range) b-carotene, men: 0.397 (0.100-1.360), women: 0.933 (0.313-2.384) µM; a-carotene, men: 0.098 (0.030-0.315); women: 0.157 (0.037-0.466); lycopene, men: 0.300 (0.074-0.987), women: 0.430 (0.123-1.229) µM.	b-carotene high vs low tertile, all cause mortality HR=0.44 (0.22-0.85), cancer mortality HR=0.36 (0.13-0.95) a-carotene no signif diff.; lycopene high vs low tertile women all cause mortality HR,mid=0.38 (0.16-0.87) HR,high=0.34 (0.13-0.94)	The women had higher levels of all 3 carotenes than men. The results weren't significant in women only because the numbers were so small. All cause mortality rates for women in tertiles of b-carotene were 34.9, 14.1, 19.8 (low to high). Cancer mortality rates were 15, 3.5 and 4.4 (6, 2 and 2 deaths).
Ito 2005a	Hokkaido, Japan	Prospective cohort	61.1	39-79	3182	10.5	287 deaths (175M, 112W), 134 cancer (81M, 53W) 31 lung, 21 colorectal, 20 stomach, 62 other	Age, sex, smoking, serum cholesterol, ALT activity	Comparison of Alive and Lung Cancer, geometric means. Total carotenes: 1.244 vs. 0.906, p=0.004 a-carotene: 0.141 vs. 0.104, p=0.01 b-carotene: 0.698 vs. 0.517, p=0.016 lycopene: 0.328 vs. 0.229, p=0.007 b-cryptoxanthin: 0.311 vs. 0.249, p=0.165 lutein/zeaxanthin: 0.985 vs. 0.950, p=0.775	High serum levels of alpha- and beta-carotenes and lycopene were found to marginally significantly or significantly reduce the risk for mortality rates of cancer of all sites and of colorectal cancers. All sites, total carotenes HR=0.67 (0.43-1.06), a-carotene HR=0.68 (0.48-1.05), b-carotene HR=0.64 (0.39-1.03), lycopene HR=0.61 (0.39-0.97) for high tertile vs low tertile. For colorectal cancer total carotene HR=0.21 (0.06-0.68), a-carotene HR=0.25 (0.07-0.87), b-carotene HR=0.28 (0.09-0.85), lycopene HR=0.23 (0.06-0.84)	
Ito 2006b	see entry for this study under cardiovascular	prospective cohort	61.3	39-79	3254						
Ito 2002	see entry for all cause mortality										

Study, N=111	Study, Country	Study Type	% female	Age range	Cohort Size	Follow-up, years	Cases (and controls, if applicable)	Adjustments	Range of Carotenoids	Effect Size, (95% CI) from lowest to highest	Comments
Karppi 2009	Kuopio Ischaemic Heart Disease Risk factor cohort, Finland	prospective cohort	0	middle aged	997	12.6	141 (55 prostate)	adjusted but no details in abstract	Mean conc. Is 0.15±0.14 µM, range 0 to 1.02 µM. Quartile cut-offs: <0.04, 0.13, >0.22 from Rissanen et al 2003	high vs low tertile lycopene RR=0.55 (0.34-0.89) p-trend=0.015. No signif trend w/prostate cancer. Other cancer RR=0.43 (0.23-0.79) p-trend=0.007	
**Other Cancer, N=3**											
Sakhi 2009 (head & neck)	Norway	case control with follow-up with cases	10% controls, 13% casese	35-85		4	78 (100)	stage, treatment, smoking status	Cases signif less than controls, cases even lower after radiation. Control and pre- and post-therapy here, µM a-carotene 0.107, 0.0613, 0.0567; b-carotene 0.375, 0.241, 0.2295; b-cryptoxanthin: 0.131, 0.0603, 0.0275; lutein 0.1425, 0.0956 0.051; zeaxanthin: 0.0356, 0.0224, 0.0122; lycopene: 0.533, 0.244, 0.125; Total carotenoids: 1.325, 0.703, 0.4745 µM	All carotenoids signif lower in cases than controls. Survival odds greater for progression-free survival for above median vs below median lutein HR=0.47 (0.22-0.98) p=0.04, b-cryptoxanthin HR=0.46 (0.23-0.94) p=0.03, b-carotene HR=0.45 (0.22-0.92) p=0.03, and total carotenoidHR=0.42 (0.20-0.91) p=0.03 and for overall survival for lutein HR=0.39 (0.16-0.96) p=0.04, b-cryptoxanthin HR=0.27 (0.11-0.69) p=0.006, a-carotene HR=0.36 (0.15-0.86) p=0.02, b-carotene HR=0.23 (0.08-0.62) p=0.004, and total carotenoids HR=0.38 (0.15-0.98) p=0.05	
Nomura 1997	Honolulu Heart Program, Hawaii, USA	nested case control	0		6832	20	69 (28 esophageal, 23 laryngeal, 16 oral-pharyngeal) (138 controls)	Adjusted for smoking, alcohol		The odds ratios for the highest tertile were 0.19 (95% CI 0.05-0.75) for alpha-carotene, 0.10 (0.02-0.46) for beta-carotene, 0.25 (0.06-1.04) for beta-cryptoxanthin, and 0.22 (0.05-0.88) for total carotenoids. When the cases were separated into esophageal, laryngeal, and oral-pharyngeal cancer, both alpha-carotene and beta-carotene were consistently and strongly associated with reduced risk at each site.	
Ozasa 2005 (urothelial)	Japan Collaborative Cohort Study, Japan	nested case control	64.5	40-79	39759	~10	42 (124)	Matched for age, sex, study area. Adjusted for smoking.	From another report on same cohort (Ito 2005) Men, cases geometric mean (5-95% range) total carotenoids 1.50 (0.54-3.62) controls 1.65 (0.49-4.22) µM Women, cases 2.33 (0.74-5.45), controls 2.56 (0.82-6.45) µM Cut-offs for quartiles: a-carotene: 0.032, 0.053, 0.089; b-carotene <0.14, 0.29, >0.58; lycopene: <0.04, 0.06, >0.15. For women a-carotene: <0.058, 0.09, >0.150; b-carotene <0.40, 0.74, >1.20; lycopene: <0.07, 0.11, >0.20	The OR for the highest to lowest tertile of serum concentration was 0.28 (95% CI 0.07 to 1.15, trend p = 0.08) for beta-carotene, 0.36 (95% CI 0.10 to 1.27, trend p = 0.10) for total carotenes and 0.31 (95% CI 0.09 to 1.09, trend p = 0.09) for total carotenoids	Reported ORs are not statistically significant.
**Age-related Macular Degeneration (AMD), N=3**											

Study, N=111	Study, Country	Study Type	% female	Age range	Cohort Size	Follow-up, years	Cases (and controls, if applicable)	Adjustments	Range of Carotenoids	Effect Size, (95% CI) from lowest to highest	Comments
Delcourt	POLA study, France	nested case control		≥60	899			age, sex, smoking, lipid standardized alpha-tocopheror, HDL-cholesterol, BMI	Tertile cut-offs, µM<br>a-carotene: <0.04, ≥0.20<br>b-carotene: <0.27, ≤0.97<br>b-cryptoxanthin: <0.13, ≥0.47<br>lutein: <0.18, ≥0.41<br>zeaxanthin: <0.04, ≥0.09<br>total lut/zea: <0.03, ≥0.56<br>lycopene: <0.22, ≥0.71 µM	highest quintile of plasma zeaxanthin was significantly associated with reduced risk of ARM (OR = 0.07; 95% CI: 0.01-0.58; P for trend = 0.005), nuclear cataract (OR = 0.23; 95% CI: 0.08-0.68; P for trend = 0.003) and any cataract (OR = 0.53; 95% CI: 0.31-0.89; P for trend = 0.01). ARM was significantly associated with combined plasma lutein and zeaxanthin (OR = 0.21; 95% CI: 0.05-0.79; P for trend = 0.01), and tended to be associated with plasma lutein (OR = 0.31; 95% CI: 0.09-1.07; P for trend = 0.04), whereas cataract showed no such associations. Among other carotenoids, only {beta}-carotene showed a significant negative association with nuclear cataract, but not ARM.	carotenoids inter-correlated, so even if there is a true effect of only one or two, the levels of all might seem to be correlated due to them coming together in foods.
Gale 2001	Sheffield, England	cross-section		66-75	372			age, sex, other confounding factors		risk of nuclear cataract was lowest in people with the highest plasma concentrations of alpha-carotene (odds ratio [OR], 0.5; 95% confidence interval [CI], 0.3-0.9, P for trend 0.006) or beta-carotene (OR, 0.7; 95% CI, 0.4-1.4, P for trend 0.033). Risk of cortical cataract was lowest in people with the highest plasma concentrations of lycopene (OR, 0.4; 95% CI, 0.2-0.8, P for trend 0.003), and risk of posterior subcapsular cataract was lowest in those with higher concentrations of lutein (OR, 0.5; 95% CI, 0.2-1.0, P for trend 0.012). High plasma concentrations of vitamin C, vitamin E, or the carotenoids zeaxanthin and beta-cryptoxanthin were not associated with decreased risk	
Gale 2003	Sheffield, England	cross-section	45.5	66-75	380		78 (20.5%) had AMD	age, smoking, serum cholesterol, beer consumption, history of angioplasty or coronary bypass grafting, hypermetropic refractive error.	Tertile cut-offs, µM<br>zeaxanthin: ≤0.0265, >0.0463<br>lutein: ≤0.1409, >0.2068<br>lut/zea: ≤0.1721, >0.2479	Compared with those whose plasma concentrations of **zeaxanthin** were in the highest third of the distribution, people whose plasma concentration was in the lowest third had an odds ratio for risk of age-related macular degeneration of 2.0 (95% confidence interval [CI] 1.0-4.1), after adjustment for age and other risk factors. Risk of age-related macular degeneration was increased in people with the lowest plasma concentrations of **lutein plus zeaxanthin** (odds ratio [OR] 1.9, 95% CI 0.9-3.5) and in those with the lowest concentrations of **lutein** (OR 1.7, 95% CI 0.9-3.3), but neither of these relations was statistically significant.	did not measure all of the carotenoids

Supplementary Materials

Additional file 2

Bibliography for Summary Overview of Plasma Carotenoids and Health Outcomes

Bibliography containing 111 references in Additional File 1.pdf.

1. Akbaraly, N.T.; Faure, H.; Gourlet, V.; Favier, A.; Berr, C. Plasma Carotenoid Levels and Cognitive Performance in an Elderly Population: Results of the EVA Study. *J Gerontol A Biol Sci Med Sci* **2007**, *62*, 308-316.
2. Akbaraly, T.N.; Favier, A.; Berr, C. Total Plasma Carotenoids and Mortality in the Elderly: Results of the Epidemiology of Vascular Ageing (EVA) Study. *British Journal of Nutrition* **2009**, *101*, 86-92.
3. Akbaraly, T.N.; Fontbonne, A.; Favier, A.; Berr, C. Plasma Carotenoids and Onset of Dysglycemia in an Elderly Population. *Diabetes Care* **2008**, *31*, 1355-1359.
4. Alfonso, H.S.; Fritschi, L.; de Klerk, N.H.; Ambrosini, G.; Beilby, J.; Olsen, N.; Musk, A.W. Plasma retinol, carotene and vitamin E concentrations and lung function in a crocidolite-exposed cohort from Wittenoom, Western Australia: a cohort study. *Nutr J* **2005**, *4*, 16-16.
5. Alfonso, H.S.; Fritschi, L.; de Klerk, N.H.; Ambrosini, G.L.; Beilby, J.; Olsen, N.; Musk, A.W. Plasma vitamin concentrations and incidence of mesothelioma and lung cancer in individuals exposed to crocidolite at Wittenoom, Western Australia. *Eur. J. Cancer Prev* **2006**, *15*, 290-294.
6. Alipanah, N.; Varadhan, R.; Sun, K.; Ferrucci, L.; Fried, L.P.; Semba, R.D. Low serum carotenoids are associated with a decline in walking speed in older women. *J Nutr Health Aging* **2009**, *13*, 170-175.
7. Ärnlöv, J.; Zethelius, B.; Risérus, U.; Basu, S.; Berne, C.; Vessby, B.; Alfthan, G.; Helmersson, J. Serum and dietary β-carotene and α-tocopherol and incidence of type 2 diabetes mellitus in a community-based study of Swedish men: report from the Uppsala Longitudinal Study of Adult Men (ULSAM) study. *Diabetologia* **2008**, *52*, 97-105.
8. Bates, C.J.; Hamer, M.; Mishra, G.D. Redox-Modulatory Vitamins and Minerals That Prospectively Predict Mortality in Older British People: The National Diet and Nutrition Survey of People Aged 65 Years and Over. *British Journal of Nutrition* **2011**, *105*, 123-132.
9. Beydoun, M.A.; Shroff, M.R.; Chen, X.; Beydoun, H.A.; Wang, Y.; Zonderman, A.B. Serum Antioxidant Status Is Associated with Metabolic Syndrome among U.S. Adults in Recent National Surveys. *The Journal of Nutrition* **2011**.
10. Bingham, S.; Luben, R.; Welch, A.; Low, Y.L.; Khaw, K.T.; Wareham, N.; Day, N. Associations between dietary methods and biomarkers, and between fruits and vegetables and risk of ischaemic heart disease, in the EPIC Norfolk Cohort Study. *Int. J. Epidemiol.* **2008**, *37*, 978-987.
11. Bingham, S.A.; Luben, R.; Welch, A.; Wareham, N.; Khaw, K.-T.; Day, N. Are imprecise methods obscuring a relation between fat and breast cancer? *Lancet* **2003**, *362*, 212-4.
12. Block, G.; Norkus, E.; Hudes, M.; Mandel, S.; Helzlsouer, K. Which Plasma Antioxidants Are Most Related to Fruit and Vegetable Consumption? *Am. J. Epidemiol.* **2001**, *154*, 1113-1118.
13. Chalabi, N.; Satih, S.; Delort, L.; Bignon, Y.-J.; Bernard-Gallon, D.J. Expression profiling by whole-genome microarray hybridization reveals differential gene expression in breast cancer cell lines after lycopene exposure. *Biochim. Biophys. Acta* **2007**, *1769*, 124-130.

14. Chang, S.; Erdman, J.W., Jr.; Clinton, S.K.; Vadiveloo, M.; Strom, S.S.; Yamamura, Y.; Duphorne, C.M.; Spitz, M.R.; Amos, C.I.; Contois, J.H.; Gu, X.; Babaian, R.J.; Scardino, P.T.; Hursting, S.D. Relationship between plasma carotenoids and prostate cancer. *Nutr Cancer* **2005**, *53*, 127-134.
15. Ching, S.; Ingram, D.; Hahnel, R.; Beilby, J.; Rossi, E. Serum Levels of Micronutrients, Antioxidants and Total Antioxidant Status Predict Risk of Breast Cancer in a Case Control Study. *J. Nutr.* **2002**, *132*, 303-306.
16. Collins, A.R.; Olmedilla, B.; Southon, S.; Granado, F.; Duthie, S.J. Serum carotenoids and oxidative DNA damage in human lymphocytes. *Carcinogenesis* **1998**, *19*, 2159-62.
17. Comstock, G.W.; Alberg, A.J.; Huang, H.Y.; Wu, K.; Burke, A.E.; Hoffman, S.C.; Norkus, E.P.; Gross, M.; Cutler, R.G.; Morris, J.S.; Spate, V.L.; Helzlsouer, K.J. The risk of developing lung cancer associated with antioxidants in the blood: ascorbic acid, carotenoids, alpha-tocopherol, selenium, and total peroxyl radical absorbing capacity. *Cancer Epidemiology Biomarkers & Prevention* **1997**, *6*, 907 -916.
18. Comstock, G.W.; Helzlsouer, K.J.; Bush, T.L. Prediagnostic serum levels of carotenoids and vitamin E as related to subsequent cancer in Washington County, Maryland. *Am. J. Clin. Nutr* **1991**, *53*, 260S-264S.
19. Connett, J.E.; Kuller, L.H.; Kjelsberg, M.O.; Polk, B.F.; Collins, G.; Rider, A.; Hulley, S.B. Relationship between carotenoids and cancer. The Multiple Risk Factor Intervention Trial (MRFIT) Study. *Cancer* **1989**, *64*, 126-134.
20. Coyne, T.; Ibiebele, T.I.; Baade, P.D.; Dobson, A.; McClintock, C.; Dunn, S.; Leonard, D.; Shaw, J. Diabetes mellitus and serum carotenoids: findings of a population-based study in Queensland, Australia. *The American Journal of Clinical Nutrition* **2005**, *82*, 685 -693.
21. Coyne, T.; Ibiebele, T.I.; Baade, P.D.; McClintock, C.S.; Shaw, J.E. Metabolic Syndrome and Serum Carotenoids: Findings of a Cross-Sectional Study in Queensland, Australia. *British Journal of Nutrition* **2009**, *102*, 1668-1677.
22. Czernichow, S.; Couthouis, A.; Bertrais, S.; Vergnaud, A.-C.; Dauchet, L.; Galan, P.; Hercberg, S. Antioxidant supplementation does not affect fasting plasma glucose in the Supplementation with Antioxidant Vitamins and Minerals (SU.VI.MAX) study in France: association with dietary intake and plasma concentrations. *The American Journal of Clinical Nutrition* **2006**, *84*, 395 -399.
23. Czernichow, S.; Vergnaud, A.-C.; Galan, P.; Arnaud, J.; Favier, A.; Faure, H.; Huxley, R.; Hercberg, S.; Ahluwalia, N. Effects of long-term antioxidant supplementation and association of serum antioxidant concentrations with risk of metabolic syndrome in adults. *The American Journal of Clinical Nutrition* **2009**, *90*, 329 -335.
24. D'Odorico, A.; Martines, D.; Kiechl, S.; Egger, G.; Oberhollenzer, F.; Bonvicini, P.; Sturniolo, G.C.; Naccarato, R.; Willeit, J. High plasma levels of alpha- and beta-carotene are associated with a lower risk of atherosclerosis: results from the Bruneck study. *Atherosclerosis* **2000**, *153*, 231-239.
25. De Waart, F.; Schouten, E.; Stalenhoef, A.; Kok, F. Serum carotenoids, α-tocopherol and mortality risk in a prospective study among Dutch elderly. *International Journal of Epidemiology* **2001**, *30*, 136 -143.
26. Delcourt, C.; Carriere, I.; Delage, M.; Barberger-Gateau, P.; Schalch, W.; the POLA Study Group Plasma Lutein and Zeaxanthin and Other Carotenoids as Modifiable Risk Factors for Age-

Related Maculopathy and Cataract: The POLA Study. *Invest. Ophthalmol. Vis. Sci.* **2006**, *47*, 2329-2335.
27. Dorjgochoo, T.; Gao, Y.-T.; Chow, W.-H.; Shu, X.-O.; Li, H.; Yang, G.; Cai, Q.; Rothman, N.; Cai, H.; Franke, A.A.; Zheng, W.; Dai, Q. Plasma carotenoids, tocopherols, retinol and breast cancer risk: results from the Shanghai Women Health Study (SWHS). *Breast Cancer Res Treat* **2009**, *117*, 381-389.
28. Dwyer, J.H.; Navab, M.; Dwyer, K.M.; Hassan, K.; Sun, P.; Shircore, A.; Hama-Levy, S.; Hough, G.; Wang, X.; Drake, T.; Merz, C.N.B.; Fogelman, A.M. Oxygenated Carotenoid Lutein and Progression of Early Atherosclerosis : The Los Angeles Atherosclerosis Study. *Circulation* **2001**, *103*, 2922-2927.
29. Dwyer, J.H.; Paul-Labrador, M.J.; Fan, J.; Shircore, A.M.; Merz, C.N.B.; Dwyer, K.M. Progression of Carotid Intima-Media Thickness and Plasma Antioxidants: The Los Angeles Atherosclerosis Study. *Arterioscler Thromb Vasc Biol* **2004**, *24*, 313-319.
30. Eichholzer, M.; Stähelin, H.B.; Gey, K.F.; Lüdin, E.; Bernasconi, F. Prediction of male cancer mortality by plasma levels of interacting vitamins: 17-year follow-up of the prospective Basel study. *Int. J. Cancer* **1996**, *66*, 145-150.
31. Epplein, M.; Franke, A.A.; Cooney, R.V.; Morris, J.S.; Wilkens, L.R.; Goodman, M.T.; Murphy, S.P.; Henderson, B.E.; Kolonel, L.N.; Le Marchand, L. Association of Plasma Micronutrient Levels and Urinary Isoprostane with Risk of Lung Cancer: The Multiethnic Cohort Study. *Cancer Epidemiology Biomarkers & Prevention* **2009**, *18*, 1962-1970.
32. Epplein, M.; Shvetsov, Y.; Wilkens, L.; Franke, A.; Cooney, R.; Le Marchand, L.; Henderson, B.; Kolonel, L.; Goodman, M. Plasma carotenoids, retinol, and tocopherols and postmenopausal breast cancer risk in the Multiethnic Cohort Study: a nested case-control study. *Breast Cancer Research* **2009**, *11*, R49.
33. Ford, E.S.; Liu, S.; Mannino, D.M.; Giles, W.H.; Smith, S.J. C-reactive protein concentration and concentrations of blood vitamins, carotenoids, and selenium among United States adults. *Eur J Clin Nutr* **2003**, *57*, 1157-1163.
34. Ford, E.S.; Will, J.C.; Bowman, B.A.; Narayan, K.M. Diabetes mellitus and serum carotenoids: findings from the Third National Health and Nutrition Examination Survey. *Am. J. Epidemiol* **1999**, *149*, 168-176.
35. Ford, E.S.; Giles, W.H.; Mokdad, A.H.; Ajani, U.A. Microalbuminuria and concentrations of antioxidants among US adults. *Am. J. Kidney Dis* **2005**, *45*, 248-255.
36. Ford, E.S.; Mokdad, A.H.; Giles, W.H.; Brown, D.W. The Metabolic Syndrome and Antioxidant Concentrations. *Diabetes* **2003**, *52*, 2346 -2352.
37. Freedman, L.S.; Potischman, N.; Kipnis, V.; Midthune, D.; Schatzkin, A.; Thompson, F.E.; Troiano, R.P.; Prentice, R.; Patterson, R.; Carroll, R.; Subar, A.F. A comparison of two dietary instruments for evaluating the fat-breast cancer relationship. *Int J Epidemiol* **2006**, *35*, 1011-21.
38. Gale, C.R.; Hall, N.F.; Phillips, D.I.; Martyn, C.N. Plasma antioxidant vitamins and carotenoids and age-related cataract. *Ophthalmology* **2001**, *108*, 1992-1998.
39. Gale, C.R.; Hall, N.F.; Phillips, D.I.W.; Martyn, C.N. Lutein and Zeaxanthin Status and Risk of Age-Related Macular Degeneration. *Invest. Ophthalmol. Vis. Sci.* **2003**, *44*, 2461-2465.

40. Gann, P.H.; Ma, J.; Giovannucci, E.; Willett, W.; Sacks, F.M.; Hennekens, C.H.; Stampfer, M.J. Lower Prostate Cancer Risk in Men with Elevated Plasma Lycopene Levels. *Cancer Research* **1999**, *59*, 1225 -1230.
41. Gill, J.K.; Franke, A.A.; Morris, J.S.; Cooney, R.V.; Wilkens, L.R.; Le Marchand, L.; Goodman, M.T.; Henderson, B.E.; Kolonel, L.N. Association of selenium, tocopherols, carotenoids, retinol, and 15-isoprostane F2t in serum or urine with prostate cancer risk: the multiethnic cohort. *Cancer Causes Control* **2009**, *20*, 1161-1171.
42. Goodman, G.E.; Schaffer, S.; Omenn, G.S.; Chen, C.; King, I. The Association between Lung and Prostate Cancer Risk, and Serum Micronutrients. *Cancer Epidemiology Biomarkers & Prevention* **2003**, *12*, 518 -526.
43. Goodman, M.T.; Kiviat, N.; McDuffie, K.; Hankin, J.H.; Hernandez, B.; Wilkens, L.R.; Franke, A.; Kuypers, J.; Kolonel, L.N.; Nakamura, J.; Ing, G.; Branch, B.; Bertram, C.C.; Kamemoto, L.; Sharma, S.; Killeen, J. The association of plasma micronutrients with the risk of cervical dysplasia in Hawaii. *Cancer Epidemiol. Biomarkers Prev* **1998**, *7*, 537-544.
44. Hak, A.E.; Stampfer, M.J.; Campos, H.; Sesso, H.D.; Gaziano, J.M.; Willett, W.; Ma, J. Plasma carotenoids and tocopherols and risk of myocardial infarction in a low-risk population of US male physicians. *Circulation* **2003**, *108*, 802-7.
45. Holick, C.N.; Michaud, D.S.; Stolzenberg-Solomon, R.; Mayne, S.T.; Pietinen, P.; Taylor, P.R.; Virtamo, J.; Albanes, D. Dietary Carotenoids, Serum β-Carotene, and Retinol and Risk of Lung Cancer in the Alpha-Tocopherol, Beta-Carotene Cohort Study. *American Journal of Epidemiology* **2002**, *156*, 536 -547.
46. Hozawa, A.; Jacobs, D.R.; Steffes, M.W.; Gross, M.D.; Steffen, L.M.; Lee, D.-H. Circulating Carotenoid Concentrations and Incident Hypertension: The Coronary Artery Risk Development in Young Adults (CARDIA) study. *J Hypertens* **2009**, *27*, 237-242.
47. Hozawa, A.; Jacobs, D.R.; Steffes, M.W.; Gross, M.D.; Steffen, L.M.; Lee, D.-H. Associations of Serum Carotenoid Concentrations with the Development of Diabetes and with Insulin Concentration: Interaction with Smoking: The Coronary Artery Risk Development in Young Adults (CARDIA) Study. *Am. J. Epidemiol.* **2006**, *163*, 929-937.
48. Hozawa, A.; Jacobs, D.R.; Steffes, M.W.; Gross, M.D.; Steffen, L.M.; Lee, D.-H. Relationships of Circulating Carotenoid Concentrations with Several Markers of Inflammation, Oxidative Stress, and Endothelial Dysfunction: The Coronary Artery Risk Development in Young Adults (CARDIA)/Young Adult Longitudinal Trends in Antioxidants (YALTA) Study. *Clin Chem* **2007**, *53*, 447-455.
49. Hsing, A.W.; Comstock, G.W.; Abbey, H.; Polk, B.F. Serologic precursors of cancer. Retinol, carotenoids, and tocopherol and risk of prostate cancer. *J. Natl. Cancer Inst* **1990**, *82*, 941-946.
50. Hu, P.; Bretsky, P.; Crimmins, E.M.; Guralnik, J.M.; Reuben, D.B.; Seeman, T.E. Association Between Serum Beta-Carotene Levels and Decline of Cognitive Function in High-Functioning Older Persons With or Without Apolipoprotein E 4 Alleles: MacArthur Studies of Successful Aging. *The Journals of Gerontology Series A: Biological Sciences and Medical Sciences* **2006**, *61*, 616 -620.
51. Hu, P.; Reuben, D.B.; Crimmins, E.M.; Harris, T.B.; Huang, M.-H.; Seeman, T.E. The Effects of Serum Beta-Carotene Concentration and Burden of Inflammation on All-Cause Mortality Risk in

High-Functioning Older Persons: MacArthur Studies of Successful Aging. *The Journals of Gerontology Series A: Biological Sciences and Medical Sciences* **2004**, *59*, M849 -M854.
52. Huang, H.-Y.; Alberg, A.J.; Norkus, E.P.; Hoffman, S.C.; Comstock, G.W.; Helzlsouer, K.J. Prospective Study of Antioxidant Micronutrients in the Blood and the Risk of Developing Prostate Cancer. *American Journal of Epidemiology* **2003**, *157*, 335 -344.
53. Hughes, K.J.; Mayne, S.T.; Blumberg, J.B.; Ribaya-Mercado, J.D.; Johnson, E.J.; Cartmel, B. Plasma Carotenoids and Biomarkers of Oxidative Stress in Patients with prior Head and Neck Cancer. *Biomark Insights* **2009**, *4*, 17-26.
54. Ito, Y.; Gajalakshmi, K.C.; Sasaki, R.; Suzuki, K.; Shanta, V. A study on serum carotenoid levels in breast cancer patients of Indian women in Chennai (Madras), India. *J Epidemiol* **1999**, *9*, 306-314.
55. Ito, Y.; Suzuki, K. The effect of serum carotenoids on atrophic gastritis among the inhabitants of a rural area in Hokkaido, Japan. *Environ Health Prev Med* **2001**, *6*, 184-188.
56. Ito, Y.; Suzuki, S.; Yagyu, K.; Sasaki, R.; Suzuki, K.; Aoki, K. Relationship between serum carotenoid levels and cancer death rates in the residents, living in a rural area of Hokkaido, Japan. *J Epidemiol* **1997**, *7*, 1-8.
57. Ito, Y.; Kurata, M.; Hioki, R.; Suzuki, K.; Ochiai, J.; Aoki, K. Cancer mortality and serum levels of carotenoids, retinol, and tocopherol: a population-based follow-up study of inhabitants of a rural area of Japan. *Asian Pac. J. Cancer Prev* **2005**, *6*, 10-15.
58. Ito, Y.; Kurata, M.; Suzuki, K.; Hamajima, N.; Hishida, H.; Aoki, K. Cardiovascular disease mortality and serum carotenoid levels: a Japanese population-based follow-up study. *J Epidemiol* **2006**, *16*, 154-160.
59. Ito, Y.; Suzuki, K.; Ishii, J.; Hishida, H.; Tamakoshi, A.; Hamajima, N.; Aoki, K. A population-based follow-up study on mortality from cancer or cardiovascular disease and serum carotenoids, retinol and tocopherols in Japanese inhabitants. *Asian Pac. J. Cancer Prev* **2006**, *7*, 533-546.
60. Ito, Y.; Suzuki, K.; Suzuki, S.; Sasaki, R.; Otani, M.; Aoki, K. Serum antioxidants and subsequent mortality rates of all causes or cancer among rural Japanese inhabitants. *Int J Vitam Nutr Res* **2002**, *72*, 237-250.
61. Ito, Y.; Wakai, K.; Suzuki, K.; Ozasa, K.; Watanabe, Y.; Seki, N.; Ando, M.; Nishino, Y.; Kondo, T.; Ohno, Y.; Tamakoshi, A. Lung cancer mortality and serum levels of carotenoids, retinol, tocopherols, and folic acid in men and women: a case-control study nested in the JACC Study. *J Epidemiol* **2005**, *15 Suppl 2*, S140-149.
62. Ito, Y.; Wakai, K.; Suzuki, K.; Tamakoshi, A.; Seki, N.; Ando, M.; Nishino, Y.; Kondo, T.; Watanabe, Y.; Ozasa, K.; Ohno, Y.; JACC Study Group Serum carotenoids and mortality from lung cancer: a case-control study nested in the Japan Collaborative Cohort (JACC) Study. *Cancer Science* **2003**, *94*, 57-63.
63. Jenab, M.; Riboli, E.; Ferrari, P.; Friesen, M.; Sabate, J.; Norat, T.; Slimani, N.; Tjonneland, A.; Olsen, A.; Overvad, K.; Boutron-Ruault, M.-C.; Clavel-Chapelon, F.; Boeing, H.; Schulz, M.; Linseisen, J.; Nagel, G.; Trichopoulou, A.; Naska, A.; Oikonomou, E.; Berrino, F.; Panico, S.; Palli, D.; Sacerdote, C.; Tumino, R.; Peeters, P.H.; Numans, M.E.; Bueno-de-Mesquita, H.B.; Buchner, F.L.; Lund, E.; Pera, G.; Chirlaque, M.D.; Sanchez, M.-J.; Arriola, L.; Barricarte, A.; Quiros, J.R.; Johansson, I.; Johansson, A.; Berglund, G.; Bingham, S.; Khaw, K.-T.; Allen, N.; Key, T.; Carneiro, F.; Save, V.; Giudice, G.D.; Plebani, M.; Kaaks, R.; Gonzalez, C.A. Plasma

and dietary carotenoid, retinol and tocopherol levels and the risk of gastric adenocarcinomas in the European prospective investigation into cancer and nutrition. *Br J Cancer* **2006**, *95*, 406-415.
64. Jiang, J.; Suzuki, S.; Xiang, J.; Kuriki, K.; Hosono, A.; Arakawa, K.; Wang, J.; Nagaya, T.; Kojima, M.; Katsuda, N.; Tokudome, S. Plasma carotenoid, alpha-tocopherol and retinol concentrations and risk of colorectal adenomas: A case-control study in Japan. *Cancer Lett* **2005**, *226*, 133-141.
65. Kabat, G.C.; Kim, M.; Adams-Campbell, L.L.; Caan, B.J.; Chlebowski, R.T.; Neuhouser, M.L.; Shikany, J.M.; Rohan, T.E. Longitudinal study of serum carotenoid, retinol, and tocopherol concentrations in relation to breast cancer risk among postmenopausal women. *The American Journal of Clinical Nutrition* **2009**, *90*, 162 -169.
66. Karppi, J.; Kurl, S.; Nurmi, T.; Rissanen, T.H.; Pukkala, E.; Nyyssönen, K. Serum lycopene and the risk of cancer: the Kuopio Ischaemic Heart Disease Risk Factor (KIHD) study. *Ann Epidemiol* **2009**, *19*, 512-518.
67. Kato, I.; Ren, J.; Visscher, D.W.; Djuric, Z. Nutritional predictors for cellular nipple aspirate fluid: Nutrition and Breast Health Study. *Breast Cancer Res. Treat* **2006**, *97*, 33-39.
68. Key, T.J.; Appleby, P.N.; Allen, N.E.; Travis, R.C.; Roddam, A.W.; Jenab, M.; Egevad, L.; Tjonneland, A.; Johnsen, N.F.; Overvad, K.; Linseisen, J.; Rohrmann, S.; Boeing, H.; Pischon, T.; Psaltopoulou, T.; Trichopoulou, A.; Trichopoulos, D.; Palli, D.; Vineis, P.; Tumino, R.; Berrino, F.; Kiemeney, L.; Bueno-de-Mesquita, H.B.; Quiros, J.R.; Gonzalez, C.A.; Martinez, C.; Larranaga, N.; Chirlaque, M.D.; Ardanaz, E.; Stattin, P.; Hallmans, G.; Khaw, K.-T.; Bingham, S.; Slimani, N.; Ferrari, P.; Rinaldi, S.; Riboli, E. Plasma carotenoids, retinol, and tocopherols and the risk of prostate cancer in the European Prospective Investigation into Cancer and Nutrition study. *Am J Clin Nutr* **2007**, *86*, 672-681.
69. Lauretani, F.; Semba, R.D.; Dayhoff-Brannigan, M.; Corsi, A.M.; Di Iorio, A.; Buiatti, E.; Bandinelli, S.; Guralnik, J.M.; Ferrucci, L. Low total plasma carotenoids are independent predictors of mortality among older persons : The InCHIANTI study. *Eur J Nutr* **2008**, *47*, 335-40.
70. Li, C.; Ford, E.S.; Zhao, G.; Balluz, L.S.; Giles, W.H.; Liu, S. Serum {alpha}-Carotene Concentrations and Risk of Death Among US Adults: The Third National Health and Nutrition Examination Survey Follow-up Study. *Arch Intern Med* **2010**, archinternmed.2010.440.
71. Lu, Q.-Y.; Hung, J.-C.; Heber, D.; Go, V.L.W.; Reuter, V.E.; Cordon-Cardo, C.; Scher, H.I.; Marshall, J.R.; Zhang, Z.-F. Inverse Associations between Plasma Lycopene and Other Carotenoids and Prostate Cancer. *Cancer Epidemiology Biomarkers & Prevention* **2001**, *10*, 749 -756.
72. Maillard, V.; Kuriki, K.; Lefebvre, B.; Boutron Ruault, M.; Lenoir, G.M.; Joulin, V.; Clavel Chapelon, F.; Chajès, V. Serum carotenoid, tocopherol and retinol concentrations and breast cancer risk in the E3N EPIC study. *International Journal of Cancer* **2010**, *127*, 1188-1196.
73. Mayne, S.; Cartmel, B.; Lin, H.; Zheng, T.; Goodwin, W.J. Low Plasma Lycopene Concentration is Associated with Increased Mortality in a Cohort of Patients with Prior Oral, Pharynx or Larynx Cancers. *J Am Coll Nutr* **2004**, *23*, 34-42.
74. McCann, J.C.; Ames, B.N. Vitamin K, an example of triage theory: is micronutrient inadequacy linked to diseases of aging? *Am J Clin Nutr* **2009**, *90*, 889-907.

75. Menkes, M.S.; Comstock, G.W.; Vuilleumier, J.P.; Helsing, K.J.; Rider, A.A.; Brookmeyer, R. Serum beta-carotene, vitamins A and E, selenium, and the risk of lung cancer. *N. Engl. J. Med* **1986**, *315*, 1250-1254.
76. Michelon, E.; Blaum, C.; Semba, R.D.; Xue, Q.-L.; Ricks, M.O.; Fried, L.P. Vitamin and carotenoid status in older women: associations with the frailty syndrome. *J. Gerontol. A Biol. Sci. Med. Sci* **2006**, *61*, 600-607.
77. Nagata, C.; Shimizu, H.; Yoshikawa, H.; Noda, K.; Nozawa, S.; Yajima, A.; Sekiya, S.; Sugimori, H.; Hirai, Y.; Kanazawa, K.; Sugase, M.; Kawana, T. Serum carotenoids and vitamins and risk of cervical dysplasia from a case-control study in Japan. *Br J Cancer* **1999**, *81*, 1234-1237.
78. Nomura, A.M.; Stemmermann, G.N.; Lee, J.; Craft, N.E. Serum micronutrients and prostate cancer in Japanese Americans in Hawaii. *Cancer Epidemiol. Biomarkers Prev* **1997**, *6*, 487-491.
79. Nomura, A.M.; Ziegler, R.G.; Stemmermann, G.N.; Chyou, P.H.; Craft, N.E. Serum micronutrients and upper aerodigestive tract cancer. *Cancer Epidemiol. Biomarkers Prev* **1997**, *6*, 407-412.
80. Nomura, A.M.Y.; Stemmermann, G.N.; Heilbrun, L.K.; Salkeld, R.M.; Vuilleumier, J.P. Serum Vitamin Levels and the Risk of Cancer of Specific Sites in Men of Japanese Ancestry in Hawaii. *Cancer Research* **1985**, *45*, 2369 -2372.
81. Ozasa, K.; Ito, Y.; Suzuki, K.; Watanabe, Y.; Hayashi, K.; Mikami, K.; Nakao, M.; Miki, T.; Mori, M.; Sakauchi, F.; Washio, M.; Kubo, T.; Wakai, K.; Tamakoshi, A. Serum carotenoids and other antioxidative substances associated with urothelial cancer risk in a nested case-control study in Japanese men. *J. Urol* **2005**, *173*, 1502-1506.
82. Palan, P.R.; Mikhail, M.S.; Goldberg, G.L.; Basu, J.; Runowicz, C.D.; Romney, S.L. Plasma levels of beta-carotene, lycopene, canthaxanthin, retinol, and alpha- and tau-tocopherol in cervical intraepithelial neoplasia and cancer. *Clin. Cancer Res* **1996**, *2*, 181-185.
83. Persson, C.; Sasazuki, S.; Inoue, M.; Kurahashi, N.; Iwasaki, M.; Miura, T.; Ye, W.; Tsugane, S.; for the JPHC Study Group Plasma levels of carotenoids, retinol and tocopherol and the risk of gastric cancer in Japan: a nested case–control study. *Carcinogenesis* **2008**, *29*, 1042 -1048.
84. Peters, U.; Leitzmann, M.F.; Chatterjee, N.; Wang, Y.; Albanes, D.; Gelmann, E.P.; Friesen, M.D.; Riboli, E.; Hayes, R.B. Serum Lycopene, Other Carotenoids, and Prostate Cancer Risk: a Nested Case-Control Study in the Prostate, Lung, Colorectal, and Ovarian Cancer Screening Trial. *Cancer Epidemiology Biomarkers & Prevention* **2007**, *16*, 962-968.
85. Rao, L.G.; Mackinnon, E.S.; Josse, R.G.; Murray, T.M.; Strauss, A.; Rao, A.V. Lycopene consumption decreases oxidative stress and bone resorption markers in postmenopausal women. *Osteoporos Int* **2006**, *18*, 109-115.
86. Ratnasinghe, D.; Forman, M.R.; Tangrea, J.A.; Qiao, Y.; Yao, S.-X.; Gunter, E.W.; Barrett, M.J.; Giffen, C.A.; Erozan, Y.; Tockman, M.S.; Taylor, P.R. Serum carotenoids are associated with increased lung cancer risk among alcohol drinkers, but not among non-drinkers in a cohort of tin miners. *Alcohol and Alcoholism* **2000**, *35*, 355 -360.
87. Reunanen, A.; Knekt, P.; Aaran, R.K.; Aromaa, A. Serum antioxidants and risk of non-insulin dependent diabetes mellitus. *Eur J Clin Nutr* **1998**, *52*, 89-93.

88. Rissanen, T.H.; Voutilainen, S.; Nyyssönen, K.; Salonen, R.; Kaplan, G.A.; Salonen, J.T. Serum lycopene concentrations and carotid atherosclerosis: the Kuopio Ischaemic Heart Disease Risk Factor Study. *The American Journal of Clinical Nutrition* **2003**, *77*, 133 -138.
89. Rock, C.L.; Natarajan, L.; Pu, M.; Thomson, C.A.; Flatt, S.W.; Caan, B.J.; Gold, E.B.; Al-Delaimy, W.K.; Newman, V.A.; Hajek, R.A.; Stefanick, M.L.; Pierce, J.P.; for the Women's Healthy Eating and Living Study Group Longitudinal Biological Exposure to Carotenoids Is Associated with Breast Cancer-Free Survival in the Women's Healthy Eating and Living Study. *Cancer Epidemiol Biomarkers Prev* **2009**, 1055-9965.EPI-08-0809.
90. Sahyoun, N.R.; Jacques, P.F.; Russell, R.M. Carotenoids, vitamins C and E, and mortality in an elderly population. *Am. J. Epidemiol* **1996**, *144*, 501-511.
91. Sakhi, A.; Russnes, K.; Thoresen, M.; Bastani, N.; Karlsen, A.; Smeland, S.; Blomhoff, R. Pre-radiotherapy plasma carotenoids and markers of oxidative stress are associated with survival in head and neck squamous cell carcinoma patients: a prospective study. *BMC Cancer* **2009**, *9*, 458.
92. Sato, R.; Helzlsouer, K.J.; Alberg, A.J.; Hoffman, S.C.; Norkus, E.P.; Comstock, G.W. Prospective Study of Carotenoids, Tocopherols, and Retinoid Concentrations and the Risk of Breast Cancer. *Cancer Epidemiology Biomarkers & Prevention* **2002**, *11*, 451 -457.
93. Schiff, M.A.; Patterson, R.E.; Baumgartner, R.N.; Masuk, M.; van Asselt-King, L.; Wheeler, C.M.; Becker, T.M. Serum Carotenoids and Risk of Cervical Intraepithelial Neoplasia in Southwestern American Indian Women. *Cancer Epidemiology Biomarkers & Prevention* **2001**, *10*, 1219-1222.
94. Semba, R.D.; Blaum, C.; Guralnik, J.M.; Moncrief, D.T.; Ricks, M.O.; Fried, L.P. Carotenoid and vitamin E status are associated with indicators of sarcopenia among older women living in the community. *Aging Clin Exp Res* **2003**, *15*, 482-487.
95. Semba, R.D.; Bartali, B.; Zhou, J.; Blaum, C.; Ko, C.-W.; Fried, L.P. Low Serum Micronutrient Concentrations Predict Frailty Among Older Women Living in the Community. *The Journals of Gerontology Series A: Biological Sciences and Medical Sciences* **2006**, *61*, 594 -599.
96. Shardell, M.D.; Alley, D.E.; Hicks, G.E.; El-Kamary, S.S.; Miller, R.R.; Semba, R.D.; Ferrucci, L. Low-serum carotenoid concentrations and carotenoid interactions predict mortality in US adults: the Third National Health and Nutrition Examination Survey. *Nutrition Research* **2011**, *31*, 178-189.
97. Simon, J.A.; Hudes, E.S.; Tice, J.A. Relation of Serum Ascorbic Acid to Mortality Among US Adults. *J Am Coll Nutr* **2001**, *20*, 255-263.
98. Steck-Scott, S.; Forman, M.R.; Sowell, A.; Borkowf, C.B.; Albert, P.S.; Slattery, M.; Brewer, B.; Caan, B.; Paskett, E.; Iber, F.; Kikendall, W.; Marshall, J.; Shike, M.; Weissfeld, J.; Snyder, K.; Schatzkin, A.; Lanza, E.; The Polyp Prevention Trial Study Group Carotenoids, vitamin A and risk of adenomatous polyp recurrence in the polyp prevention trial. *Int. J. Cancer* **2004**, *112*, 295-305.
99. Sugiura, M.; Nakamura, M.; Ogawa, K.; Ikoma, Y.; Matsumoto, H.; Ando, F.; Shimokata, H.; Yano, M. Associations of Serum Carotenoid Concentrations with the Metabolic Syndrome: Interaction with Smoking. *British Journal of Nutrition* **2008**, *100*, 1297-1306.
100. Suzuki, K.; Ito, Y.; Otani, M.; Suzuki, S.; Aoki, K. [A study on serum carotenoid levels of people with hyperglycemia who were screened among residents living in a rural area of Hokkaido, Japan]. *Nippon Eiseigaku Zasshi* **2000**, *55*, 481-488.

101. Suzuki, K.; Inoue, T.; Hashimoto, S.; Ochiai, J.; Kusuhara, Y.; Ito, Y.; Hamajima, N. Association of serum carotenoids with high molecular weight adiponectin and inflammation markers among Japanese subjects. *Clin. Chim. Acta* **2010**, *411*, 1330-1334.
102. Suzuki, K.; Ito, Y.; Inoue, T.; Hamajima, N. Inverse association of serum carotenoids with prevalence of metabolic syndrome among Japanese. *Clin Nutr* **2011**.
103. Suzuki, K.; Ito, Y.; Nakamura, S.; Ochiai, J.; Aoki, K. Relationship between serum carotenoids and hyperglycemia: a population-based cross-sectional study. *J Epidemiol* **2002**, *12*, 357-366.
104. Tamimi, R.M.; Hankinson, S.E.; Campos, H.; Spiegelman, D.; Zhang, S.; Colditz, G.A.; Willett, W.C.; Hunter, D.J. Plasma Carotenoids, Retinol, and Tocopherols and Risk of Breast Cancer. *Am. J. Epidemiol.* **2005**, *161*, 153-160.
105. The Alpha-Tocopherol Beta Carotene Cancer Prevention Study Group The Effect of Vitamin E and Beta Carotene on the Incidence of Lung Cancer and Other Cancers in Male Smokers. *N Engl J Med* **1994**, *330*, 1029-1035.
106. Toniolo, P.; Van Kappel, A.L.; Akhmedkhanov, A.; Ferrari, P.; Kato, I.; Shore, R.E.; Riboli, E. Serum Carotenoids and Breast Cancer. *American Journal of Epidemiology* **2001**, *153*, 1142 -1147.
107. Vogt, T.M.; Mayne, S.T.; Graubard, B.I.; Swanson, C.A.; Sowell, A.L.; Schoenberg, J.B.; Swanson, G.M.; Greenberg, R.S.; Hoover, R.N.; Hayes, R.B.; Ziegler, R.G. Serum Lycopene, Other Serum Carotenoids, and Risk of Prostate Cancer in US Blacks and Whites. *Am. J. Epidemiol.* **2002**, *155*, 1023-1032.
108. Wakai, K.; Suzuki, K.; Ito, Y.; Kojima, M.; Tamakoshi, K.; Watanabe, Y.; Toyoshima, H.; Hayakawa, N.; Hashimoto, S.; Tokudome, S.; Suzuki, S.; Kawado, M.; Ozasa, K.; Tamakoshi, A. Serum carotenoids, retinol, and tocopherols, and colorectal cancer risk in a Japanese cohort: effect modification by sex for carotenoids. *Nutr Cancer* **2005**, *51*, 13-24.
109. Wang, L.; Liu, S.; Pradhan, A.D.; Manson, J.E.; Buring, J.E.; Gaziano, J.M.; Sesso, H.D. Plasma Lycopene, Other Carotenoids, and the Risk of Type 2 Diabetes in Women. *American Journal of Epidemiology* **2006**, *164*, 576 -585.
110. Yanagisawa, A.; Suzuki, K.; Kimura, A.; Ito, Y.; Hamajima, N.; Inoue, T. Possible protective effect of serum beta-carotene levels on the association between interleukin-1B C-31T polymorphism and hypertension in a Japanese population. *Clin Nutr* **2009**, *28*, 198-202.
111. Yang, Z.; Zhang, Z.; Penniston, K.L.; Binkley, N.; Tanumihardjo, S.A. Serum Carotenoid Concentrations in Postmenopausal Women from the United States with and without Osteoporosis. *Int J Vitam Nutr Res* **2008**, *78*, 105-111.
112. Ylönen, K.; Alfthan, G.; Groop, L.; Saloranta, C.; Aro, A.; Virtanen, S.M.; the Botnia Research Group Dietary intakes and plasma concentrations of carotenoids and tocopherols in relation to glucose metabolism in subjects at high risk of type 2 diabetes: the Botnia Dietary Study. *The American Journal of Clinical Nutrition* **2003**, *77*, 1434 -1441.
113. Yuan, J.-M.; Ross, R.K.; Chu, X.-D.; Gao, Y.-T.; Yu, M.C. Prediagnostic Levels of Serum β-Cryptoxanthin and Retinol Predict Smoking-related Lung Cancer Risk in Shanghai, China. *Cancer Epidemiology Biomarkers & Prevention* **2001**, *10*, 767 -773.
114. Yuan, J.-M.; Ross, R.K.; Gao, Y.-T.; Qu, Y.-H.; Chu, X.-D.; Yu, M.C. Prediagnostic Levels of Serum Micronutrients in Relation to Risk of Gastric Cancer in Shanghai, China. *Cancer Epidemiology Biomarkers & Prevention* **2004**, *13*, 1772 -1780.

115. Zhang, J.; Dhakal, I.; Stone, A.; Ning, B.; Greene, G.; Lang, N.P.; Kadlubar, F.F. Plasma Carotenoids and Prostate Cancer: A Population-Based Case-Control Study in Arkansas. *Nutrition and Cancer* **2007**, *59*, 46.
